# Identification of Biomarkers Controlling Cell Fate In Blood Cell Development

**DOI:** 10.3389/fbinf.2021.653054

**Published:** 2021-07-19

**Authors:** Maryam Nazarieh, Marc Hoeppner, Volkhard Helms

**Affiliations:** ^1^ Institute of Clinical Molecular Biology, Christian-Albrecht-University of Kiel, Kiel, Germany; ^2^ Center for Bioinformatics, Saarland University, Saarbruecken, Germany

**Keywords:** developmental genes, transcription factor, gene ontology, gene expression, cell fate, master regulator, cell lineage

## Abstract

A blood cell lineage consists of several consecutive developmental stages starting from the pluri- or multipotent stem cell to a state of terminal differentiation. Despite their importance for human biology, the regulatory pathways and gene networks that govern these differentiation processes are not yet fully understood. This is in part due to challenges associated with delineating the interactions between transcription factors (TFs) and their corresponding target genes. A possible step forward in this case is provided by the increasing amount of expression data, as a basis for linking differentiation stages and gene activities. Here, we present a novel hierarchical approach to identify characteristic expression peak patterns that global regulators excert along the differentiation path of cell lineages. Based on such simple patterns, we identified cell state-specific marker genes and extracted TFs that likely drive their differentiation. Integration of the mean expression values of stage-specific “key player” genes yielded a distinct peaking pattern for each lineage that was used to identify further genes in the dataset which behave similarly. Incorporating the set of TFs that regulate these genes led to a set of stage-specific regulators that control the biological process of cell fate. As proof of concept, we considered two expression datasets covering key differentiation events in blood cell formation of mice.

## 1 Introduction

**FIGURE 1:**
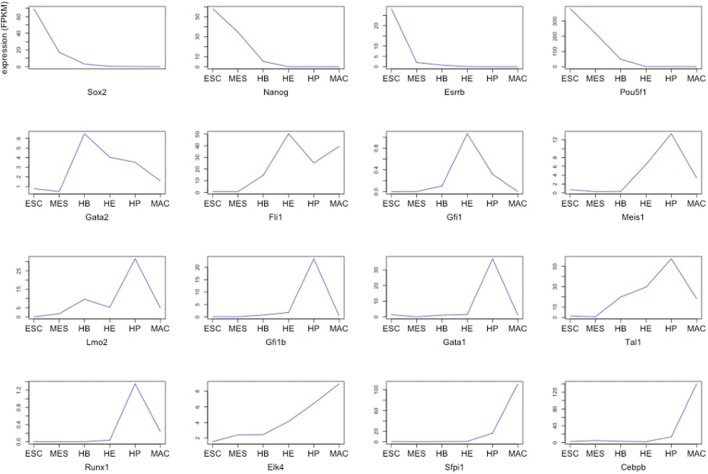
Expression of 16 global regulators driving hematopoietic specification for six stages of blood development starting from ESCs (stage 1) up to terminally differentiated macrophages (stage 6) (Goode et al., 2016).

Cell fate describes a biological program, which determines how a less specialized cell type develops into a more specialized one. For each transition out of a particular state, this involves a decision between either self-renewal or differentiation into daughter cells (Garcia-Ojalvo and Martinez Arias, 2012). It is well accepted that such processes are tightly regulated by transcriptional networks, typically centered around a discrete number of transcription factors (Moignard and Göttgens, 2014). Knowing the “key players” involved in these events may thus not only serve as a predictive marker to help determining differentiation stages of cells, but furthermore could potentially be useful for clinical purposes, for example by aiding in the search for therapeutic targets across different diseases involving aberrations in the composition of cell types or stages, respectively (An et al., 2014). One of the best-studied examples are blood cells, which are already widely used in diagnostics. Especially in complex blood-related diseases such as leukemia, understanding the manifestation of the disease and monitoring its progression and response to treatment could greatly benefit from a deeper understanding of the underlying regulatory processes and key “actors” that govern blood cell differentiation. However, delineating lineage-specific regulatory networks is a challenging task, typically requiring the costly integration of multiple data types; particularly from various “omics” technologies. Previous work using a complex multi-omics approach identified a set of 16 “global regulators” that drive the differentiation of blood cells across six discrete stages - Embryonic stem cells (ESCs), Mesoderm (MES), Hemangioblast (HB), Hemogenic endothelium (HE), Hematopoietic progenitor (HP) and Macrophages (MAC) (Goode et al., 2016). It is very plausible to assume that these global regulators stand at the top of the regulatory hierarchy and indirectly govern particular cellular identity. Interestingly, although the overall network is preserved across developmental stages, analysis of the characteristic changes in expression (Figure 1) suggests that these “global regulators” contribute differently at various stages. We have previously developed a method that reconstructs the core components of a regulatory network from gene expression data and defines a so-called “minimum dominating set” (MDS), i.e. the minimum set of TFs that control the entire network by their interactions. A modification of this concept is the “minimum connected dominating set” (MCDS), which searches for a minimum number of genes that are connected and control the underlying co-network (Nazarieh et al., 2016; Nazarieh, 2018; Nazarieh and Helms, 2019). When applied to expression data, one should expect that a key transcription factor being most strongly associated with a certain differentiation stage exhibits a peak in expression at that stage compared to the other stages of that lineage. Genes directly regulated by such a key player can be expected to somehow mimic its expression profile, thus allowing their assignment to a given regulator and cellular stage. In the present work, we introduce an approach to identify stage-specific key regulators that are likely to control cell fate in a differentiation/developmental or resistance pathway. PathDevFate is a method for identifying the set of connected influencers and connectors that play roles in cell differentiation and cell commitment. The method implements a workflow to initially identify influencers that follow a lineage-specific pattern defined by integrating cell-specific genes and TFs in a stage. Then, it identifies the set of TFs that regulate these influencers. Finally, it introduces a regulatory pathway of a connected set of influencers and connectors whereby the path length is determined by the number of influencers and connectors. We demonstrate the usefulness of this approach by the example of two expression data sets that were used to investigate blood cell differentiation in mice (Bock et al., 2012; Goode et al., 2016).

## 2 Methods

### 2.1 Overview

**FIGURE 2:**
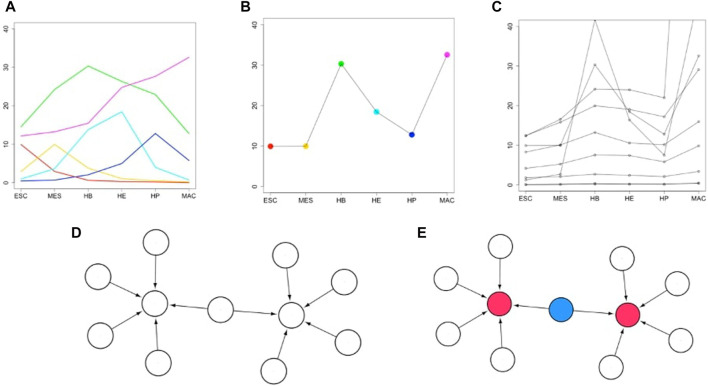
Overview of how biomarkers are identified that control or drive a developmental cell fate process. **(A)** Fictitious expression profiles (*y*-axis) of six selected transcription factors (TFs) across six developmental stages (*x*-axis). TFs are identified having peak expression in the respective stage. This step yields the stage-specific key regulators (TFs). **(B)** A lineage-specific pattern is constructed by integrating the stage-specific patterns across the lineage. **(C)** Further genes are identified having highly correlated expression profiles to one of the stage-specific key regulators of **(B)** (here, one of the TFs peaking in the terminal stage MAC). **(D)** A gene-regulatory (GRN) network is constructed including all stage-specific key regulators and their correlated target genes. This GRN includes TFs and target genes from all stages. **(E)** A regulatory pathway is identified (see methods) that connects (blue colored nodes) all “influencer” nodes (red colored nodes).

Figure 2 illustrates the workflow of the entire approach. First, we derive diagnostic expression profiles to identify genes that are likely centrally involved in a cellular differentiation path (Figure 2A). Next, we integrate the expression pattern of stage-specific developmental genes across full individual lineages (Figure 2B). From this, a set of correlated genes and associated TFs is identified (Figure 2C). This preliminary network is further refined by incorporating experimentally validated data e.g. from a TF-gene interaction database such as TRRUST (Han et al., 2017) to define a sub-regulatory network whose target genes follow the aforementioned lineage-specific expression pattern and have a well-defined TF regulator (Figure 2D). The regulatory relationships are modeled as a directed graph as is commonly done in such analyses. The source nodes are TFs and target nodes can be genes and/or TFs. The edges correspond to the regulatory interactions between them. Finally, we present an algorithm that finds the shortest regulatory path that connects the target genes that are tightly regulated by multiple TFs (Figure 2E). We suggest that the set of target genes and TFs that connect them forms an important group of driver genes for the respective cell fate process. A functional enrichment analysis is then used to investigate the biological processes these identified TFs have previously shown to be involved in. Details of the individual steps of this algorithm and the motivation behind the steps will be explained below in section 2.4.

### 2.2 Datasets

The first case study is based on genome-wide RNA-seq expression profiles (Goode et al., 2016) in form of FPKM values across six consecutive differentiation stages, namely ESC, MES, HB, EH, HP and MAC (GEO accession GSE69080). The microarray data for the second case study were published by Bock et al. (2012). As mentioned in that paper, the data were obtained as CEL files and normalized in the same order to reduce batch effects. The data includes 13 cell populations sorted by FACS analysis across 6 lineages.

### 2.3 Regulatory Relationships

Data on the relationship between TFs and their target gene(s) were taken from the TRRUST database v2 (Han et al., 2017) that was compiled based on literature curation. This release of the database includes 6552 TF-target interactions for 828 mouse TFs.

### 2.4 Workflow: Prioritization of the Candidates of the Cell Fate Process

1) As input, we use existing knowledge about a small gold-standard set of tissue-specific global regulators. All differentiation stages of the particular cell lineage under consideration are arranged in a linear sequence (see Figure 2A). First, we identify which ones of the mentioned global regulators peak in the individual stages.
2) Then, we aim at identifying an expression signature of each particular stage. For this, we now identify further genes and TFs having a closely matching expression profile to the stage-specific expression pattern of the global regulators peaking in this stage just defined. The genes identified in this manner are termed first layer candidates. (see Figure 2A).
3) After having identified sets of signature genes for particular stages, we now combine the stage-specific expression pattern across the entire lineage. The mean expression value of all identified stage-specific genes is considered as a representative for each stage. This averaging is done to capture the typical behavior of all stage-specific genes by a single profile. This then yields a lineage-specific pattern. (see Figure 2B). Alternatively, one could have normalized the expression values of all stage-specific genes to a particular interval.
4) In the next step, we find further genes and TFs having an expression pattern that closely mimics the integrated lineage-specific expression pattern. These are then termed second layer candidates. (see Figure 2C).
5) Now, we determine TFs that regulate the candidates in the second layer identified in step 4. These are then termed third layer candidates.
6) We now aggregate the regulators identified in step 5 and the target genes identified in step 4 into a regulatory subnetwork. By way of design, this network includes those genes having a particular lineage-specific expression pattern and their regulators. We consider this subnetwork of the full regulatory network of a cell as the essential part governing the fate of a particular cell lineage. (see Figure 2D).
7) Now, we determine the high-indegree nodes in the regulatory subnetwork identified in step 6. The idea behind this is that these hub genes contribute a major part of all regulatory activity in the constructed subnetwork. (see red color nodes in the Figure 2E).
8) Identify additional connector nodes that connect the nodes identified in step 7 (see blue color nodes in the Figure 2E). The idea of this step is that this connected pathway forms an equivalent of a “regulatory pathway”. This step has an analogy to the concept of an MCDS of dominating nodes that we defined in our earlier work (Nazarieh et al., 2016). The fourth layer is a regulatory subnetwork including the set of influencers and connectors.

The implemented program code for the two afore-mentioned datasets is available at: https://github.com/ikmb/KeyDevelopmentalFate in the code section: (KeyDevFate_Goode2016.R, KeydevFate_Bock2012.R)

### 2.5 Randomization Algorithm

Input: A set of correlated genes following an integrated pattern of gene expression across the stages in one lineage. Output: Overlap significance of the correlated genes based either on the original data or on the shuffled data.

1) Shuffle the data column-wise, whereby each column corresponds to the expression value of the genes in a certain stage.
2) Identify the set of correlated genes following the integrated expression pattern based on shuffled data.
3) Compute the overlap between the correlated genes in real data and the correlated genes in the shuffled data.
4) To characterize the statistical significance of the identified genes, we compared the obtained result to analogous results identified based on randomly shuffled data. If a considerable part of the originally correlated genes were also identified from shuffled data, this would suggest that the findings are insignificant. Precisely, we counted the number of times when the overlap (Jaccard index) between the correlated genes in the original data and the correlated genes in 1000 shuffled data sets is greater than 0.05. Here, the Jaccard index was computed as the ratio of the intersection between the set of correlated genes and the resampled data over the union of the two sets.

The implemented code for the first case study is available at: https://github.com/ikmb/KeyDevelopmentalFate in the code section: RandomizationAlgorithm_Goode2016.R

### 2.6 PathDevFate Algorithm: Find the Regulatory Path That Involves a Certain Set of Nodes

Input: A network that is obtained from step 6 of the above-mentioned pipeline. Output: A set of genes and TFs with assigned roles of influencers and connectors.

1) Identify the set of nodes that are regulated by at least one TF.
2) Specify a threshold (here denoted by “l”) as a measure of in-degree threshold.
3) Select the nodes whose number of incoming edges exceeds “l”. These are termed “influencers”.
4) Find a path that connects the influencer nodes by adding a minimum number of further (Steiner) nodes (“connectors”).

The implemented code for the algorithm is available at: https://github.com/ikmb/KeyDevelopmentalFate in the code section (PathDevFate) In step 2, a threshold is introduced that provides a balance between the number of influencers with respect to the number of incoming edges and the number of TFs that are supposed to connect them (which depends on the distance these influencers have from each other). This measure serves to capture the high-indegree nodes and imposes a minimum number of TFs to the regulatory pathway. The idea of this algorithm has been taken from our MCDS algorithm (Nazarieh et al., 2016). In the MCDS algorithm, after finding the dominator nodes, the next step is to find the connectors and minimise the number of dominators and connectors as long as connectivity persists and the underlying connected network is covered by the MCDS. In the new algorithm, after finding the connectors, we keep the set of influencer nodes (nodes with high in-degree) constant and then minimize the number of connector nodes. Based on the enrichment analysis, influencers take part mainly in the development and differentiation processes, whereas connectors may in addition also contribute to cell fate commitment.

### 2.7 Functional Annotation

The biological function of the genes in each stage was evaluated using the enrichment analysis tool provided by the DAVID portal of NIH (version 6.8) based on the functional categories in GO Direct (Huang et al., 2008) and all *Mus musculus* genes as background. *p*-values below the threshold of 0.05 as obtained by the hypergeometric test were adjusted for multiple testing using the Benjamini and Hochberg (BH) correction (Benjamini and Hochberg, 1995).

## 3 Results

The main goal of this study was to derive an approach that identifies a connected set of cell-fate regulating genes. For this, we implemented the hierarchical strategy illustrated in Figure 2. The first layer includes the stage-specific TFs and genes that are involved in cellular differentiation. The second layer consists of further genes and TFs following the same integrated stage-specific expression pattern. The third layer is formed by those TFs that regulate the candidates in the second layer. A regulatory network was constructed from the correlated genes following the integrated expression pattern with a set of TFs that regulate them which forms the candidates in the fourth layer. Finally, we derived a shortest regulatory path that connects the set of correlated genes that are regulated by multiple TFs (PathDevFate, see Methods). In short, the target genes that are tightly regulated by multiple transcription factors are flagged as “influencers” and the nodes that connect them as “connectors”. As proof of concept, we applied the method to two datasets of murine blood differentiation. The first case study was a lineage of six stages starting at ESC and leading to MAC (Goode et al., 2016). We then extended the concept by setting rules defined for cellular differentiation in (Artyomov et al., 2010) and applied it to expression data from across 6 murine cell lineages in blood formation (Bock et al., 2012) starting at HSC and leading to either CD4 T-cells, CD8 T-cells, B cells, erythrocytes, granulocytes or monocytes, respectively.

### 3.1 Dataset 1: Differentiation of Murine Blood Stem Cells

**FIGURE 3:**
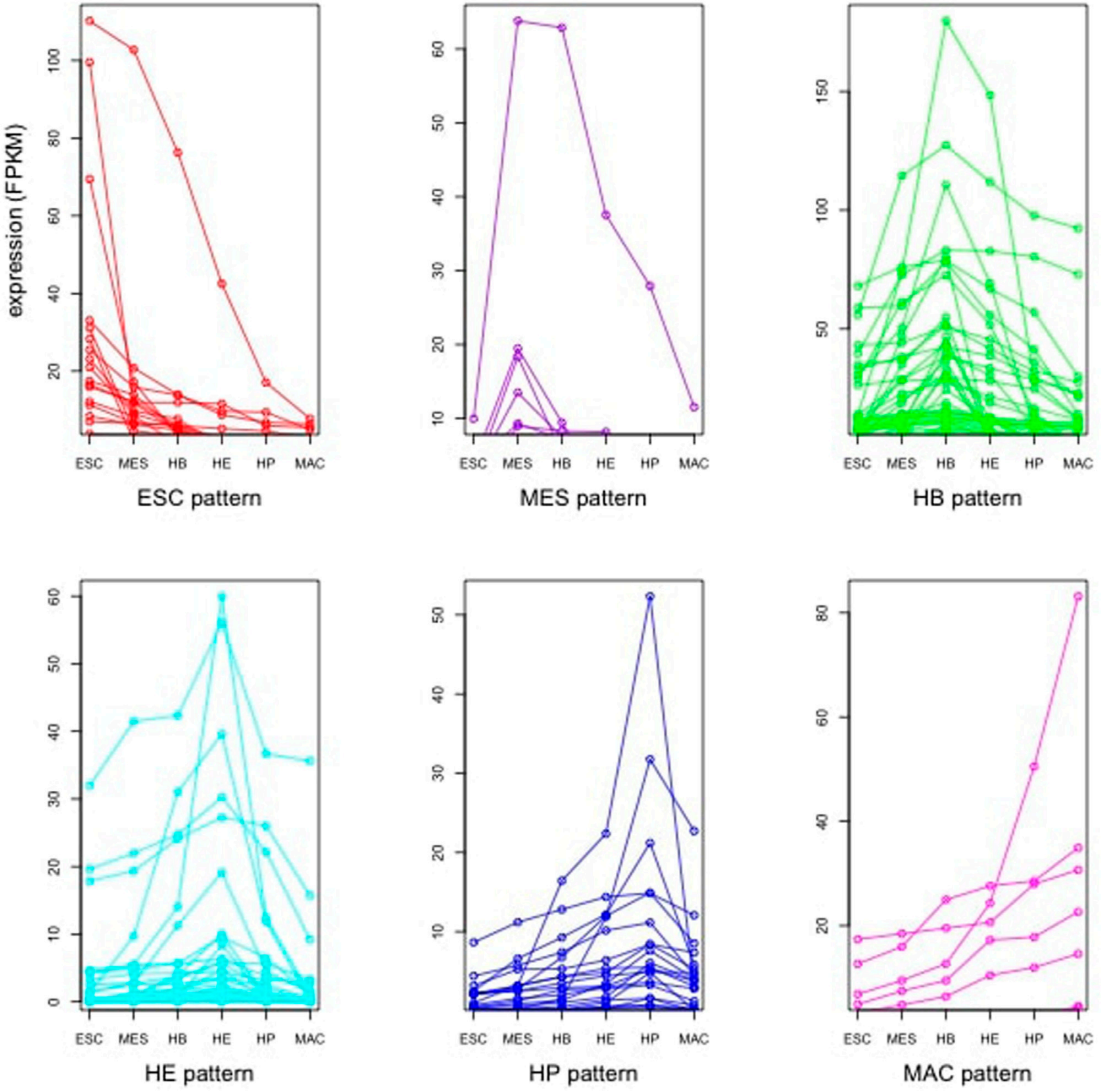
Expression pattern of identified TFs in six stages of ESC, MES, HB, HE, HP and MAC that follow the global expression pattern.

**FIGURE 4:**
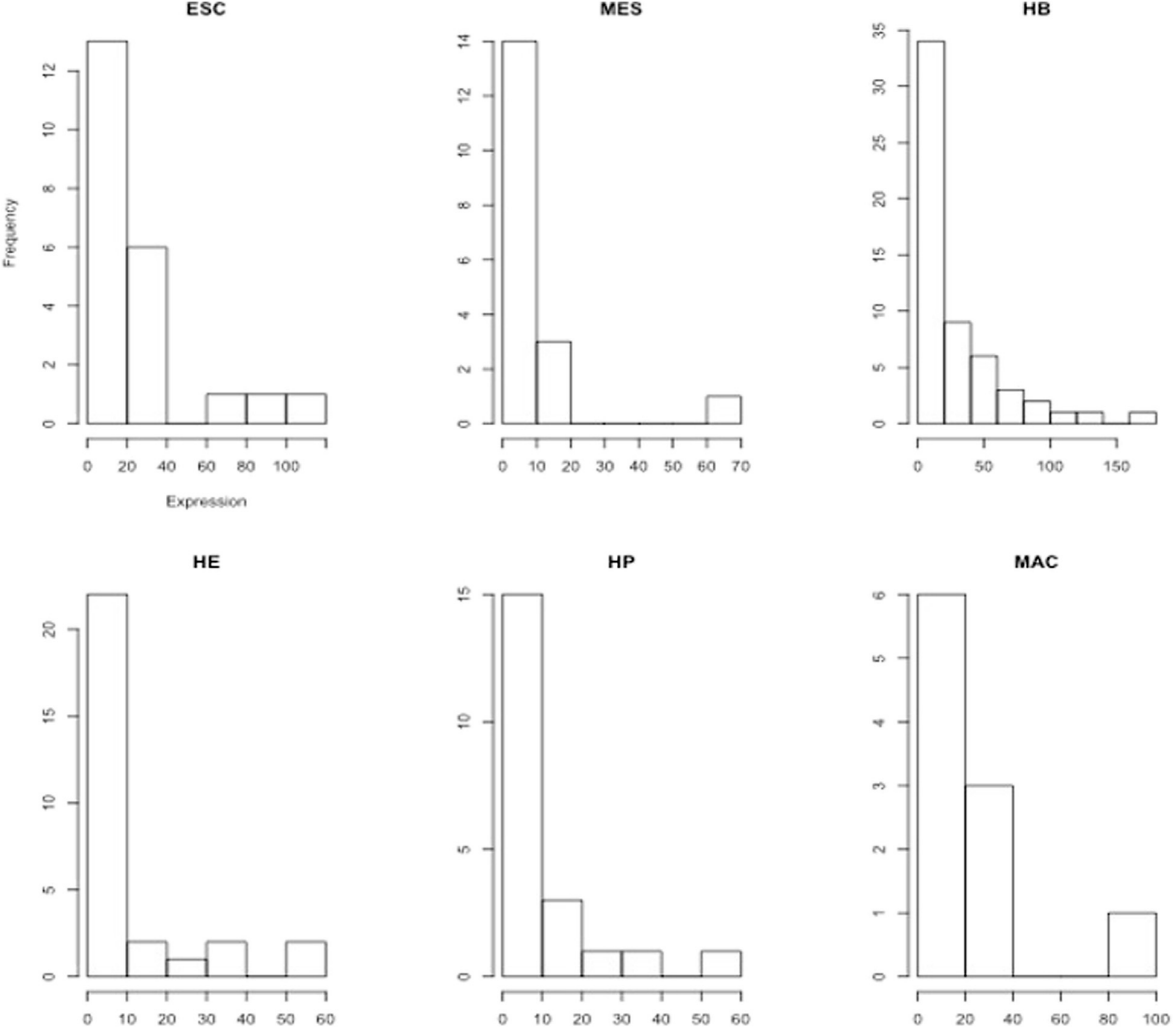
Histograms of stage-specific TF expression levels (FPKM values) in the blood cell lineage show a quasi-exponential decay. Eg. for ESC, 13 TFs have expression levels between 0 and 20, 6 TFs have expression levels between 20–40.

**FIGURE 5:**
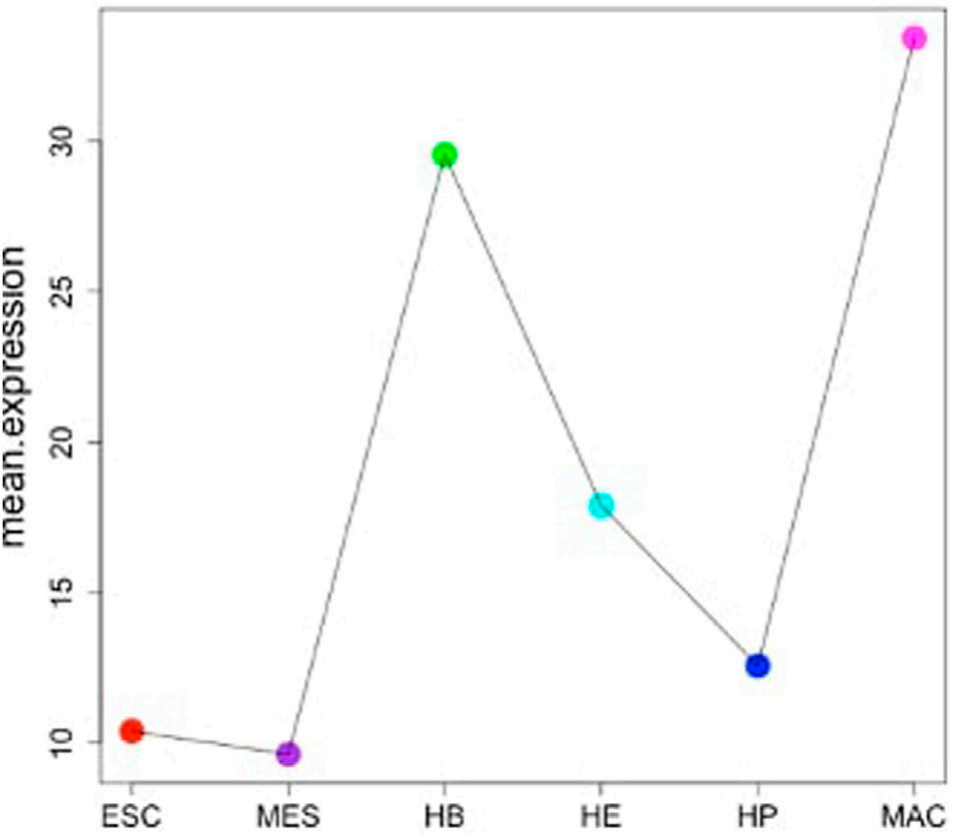
Depiction of the mean expression of stage-specific genes across six stages of blood cell differentiation (from ESC to MAC).

**FIGURE 6:**
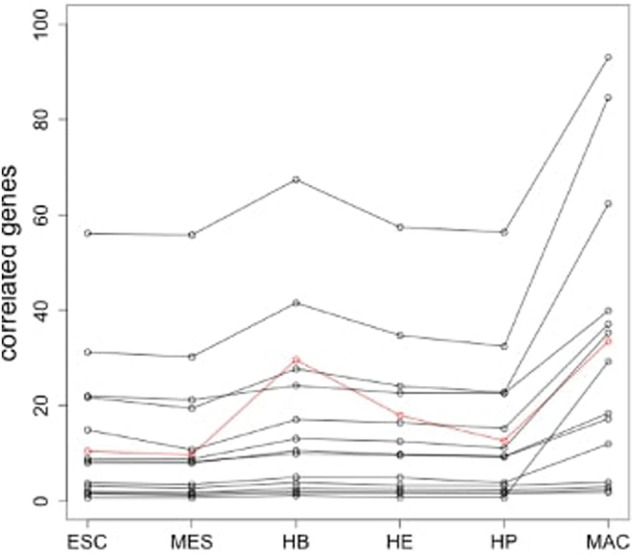
Depiction of the correlated genes. The red curve shows the pattern of integrated mean expression of all six stages in the lineage. The black curves represent correlated genes that have perfectly positive correlation based on the Spearman method (threshold = 1).

**FIGURE 7:**
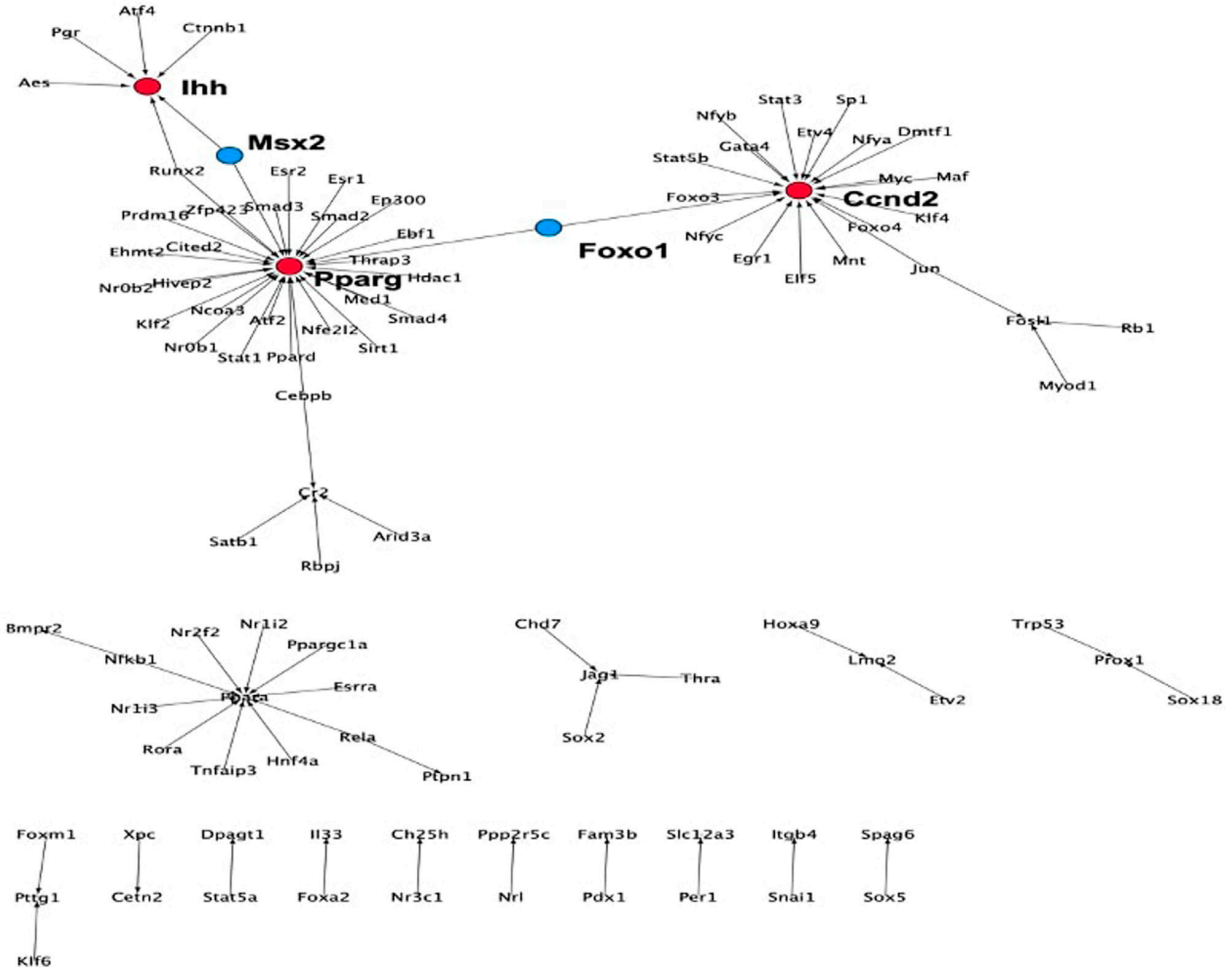
TF-target network for the set of correlated genes derived from the TRRUST database. Influencers (red nodes) are the stage-specific target genes that are regulated by more than five TFs. Connectors are TFs (blue nodes).

From published multi-omics data on murine blood stem cells (Goode et al., 2016), we retrieved the gene expression profile of 16 global regulators that were identified in the study of (Goode et al., 2016). These were averaged to yield a stage-specific expression pattern that is considered subsequently as signature pattern. We then identified further genes (and further TFs) having strongly correlated expression profiles with this stage-specific expression pattern. Figure 3 shows the expression pattern of the TFs that were among the identified correlated genes. Obviously, multiple TFs show peaks in each of the individual differentiation stages. This analysis, yielding our “first” gene layer, identified between 197 (HP) and 692 (HB) correlated gene expression profiles (Supplementary Table S1). Included in this are between 10 (MAC) and 57 (HB) TFs, such as SOX2 and ESRRB. For each stage, we considered the identified genes to reconstruct functional profiles of the correlated genes based on enriched gene ontology terms (GO) (Supplementary Tables S2–S5). In order to understand the molecular mechanisms governing each differentiation stage, we next performed a functional enrichment analysis using both gene ontology (GO) terms and KEGG pathways for the key transcription factors (Supplementary Tables S6–S11) found in each differentiation stage, as well as for their (known) target genes (Supplementary Tables S12–S17), respectively. Supplementary Table S6 for ESC lists GO terms such as stem cell differentiation (GO:0048863), multicellular organism development (GO:0007275), endoderm development (GO:0007492) and cell differentiation (GO:0030154), respectively. Moreover, the five genes Onecut1, Esrrb, Id1, Sox2, and Zic3 belong to the KEGG pathway signaling pathways regulating pluripotency of stem cells (mmu04550). Supplementary Tables S7–S11 list the enriched GO terms and KEGG pathways for the identified TFs in MES, HB, HE, HP and MAC. The lists include further specialized GO terms in addition to some of the aforementioned terms such as patterning of blood vessels (GO:0001569), cell fate commitment (GO:0045165), heart development (GO:0007507) and hemopoiesis (GO:0030097), respectively and also the KEGG pathway acute myeloid leukemia (mmu05221). Then, we inferred the set of target genes for the set of “key player” TFs at each developmental stage from the TF-gene interaction database TRRUST (Han et al., 2017). Enrichment analysis for the set of identified target genes in the ESC stage yielded the enriched biological process GO terms listed in Supplementary Tables S12. The list includes GO terms such as proliferation (GO:0042127), multicellular organism development (GO:0007275), stem cell differentiation (GO:0048863), cell differentiation (GO:0030154), cell fate commitment (GO:0045165), cell development (GO:0048468) and cell proliferation (GO:0008283), respectively. Supplementary Tables S13–S17 list the enriched GO terms for the target genes in other developmental stages. In addition to common GO terms, distinct GO terms, such as BMP signaling pathway involved in heart development (GO:0061312) and Wnt signalling pathway (GO:0016055) are added in the MES stage. More specialized GO terms appear in later stages HB, HE, and HP, such as liver development (GO:0001889), B cell lineage commitment (GO:0002326), ear development (GO:0043583) and eye development (GO:0001654), respectively. Although the TFs identified in each particular developmental stage also follow the aforementioned expression pattern, they exhibit different expression levels. The histograms in Figure 4 show the frequency of TFs based on their expression level. In general, there are many more TFs with low expression (e.g. 0–10, 0–20 etc.) than with high expression (above 50). There is an initial increase in the absolute number of patterned TFs from 13 (ESC), 14 (MES) to 34 (HB), followed by a corresponding decline over 22 (HE), 15 (HP) to 6 (MAC). Genes that act in the same biological processes are expected to (partially) share activity profiles (Huttenhower and Troyanskaya, 2008). Figure 5 shows the mean expression of the identified stage-specific genes. To the aim of identifying additional members of the candidate network, we extracted genes that mimic the same expression pattern exhibited by the stage-specific genes (Figure 5). For this, we required that their expression patterns across the six stages (from ESC to MAC) showed the same monotonic expression pattern (i.e. Spearman rank correlation larger than 0.9) as the stage-specific genes. This led to the identification of 243 genes (Supplementary Table S18) including 13 TFs. Figure 6 shows the expression pattern of those genes having perfect Spearman correlation of 1.0. The 13 TFs are considered as candidates for the second layer. To verify the statistical significance of the correlated genes, we resampled the data 1000 times, identified patterned genes in each case, and measured the overlap between the correlated genes in the original data set and those determined from the resampled data, see Supplementary Figure S1. The overlap was measured based on the Jaccard index as the ratio of intersection between the sets of correlated genes in real data and in resampled data over the union of the two sets. Only 3 out of 1,000 cases had a similarity higher than 0.05 between the correlated genes in the original data and the correlated genes in the shuffled data (*p*-value of 0.003). Thus, the stage-specific genes identified in the real data are rarely identified based on randomly shuffled data, which strengthens the biological meaningfulness of this analysis. Next, we sought to identify known regulators of this initial set of co-expressed genes using data from the TRRUST database. This analysis resulted in 83 TFs which then formed the third layer of our analysis (Supplementary Table S19). The intersection with cell-specific TFs of ESC, MES, HB, HE, HP and MAC identified in the first layer includes (Etv4, Hdac1, Prdm16, Sox2), (Foxo4), (Atf2, Etv2, Gata4, Msx2, Snail1), (Ebf1, Smad3), (Stat5a, Stat5b, Thra), (Arid3a, Stat5b), respectively. All these genes were previously reported to have specific roles in cell fate commitment (Liu et al., 1996; Avilion et al., 2003; Dunn et al., 2004; Beuling et al., 2008; Zandi et al., 2008; Ackermann et al., 2011; Rhee et al., 2014; Babajko et al., 2015; Horvay et al., 2015; Liu et al., 2015; Bourgeois and Madl, 2018; Garg et al., 2018). Supplementary Table S20 shows the functional enrichment analysis (biological process) and KEGG pathways for the 83 TFs along with *p*-values, using a hypergeometric test and adjusted for multiple testing using the Benjamini and Hochberg (BH) correction (Benjamini and Hochberg, 1995) below a threshold of ≤0.05 . Notable GO terms on this list include: GO:0008285 negative regulation of cell proliferation, GO:0008284 positive regulation of cell proliferation GO:0043066 negative regulation of apoptotic process, GO:0043065 positive regulation of apoptotic process, GO:0002360 T cell lineage commitment, GO:1902262 apoptotic process involved in patterning of blood vessels, GO:0048863 stem cell differentiation, GO:0030154 cell differentiation, GO:0007507 heart development, GO:0007275 multicellular organism development, GO:0033077 T cell differentiation in thymus, and GO:0030217 T cell differentiation. Finally, using information from the TRRUST database, a regulatory network was reconstructed whose nodes are confined to the candidates of the second and third layer. The network demonstrates the connectivity between the candidates in the second and third layer. The number of TFs in the network exceeds the number of target genes so that the network contains few genes with a high number of incoming edges. In the network having 90 interactions, the 83 regulators were taken from the third layer and 21 target genes taken from the second layer (Supplementary Table S18). This network contains the three high-indegree nodes Ccnd2, Pparg and Ihh in the largest connected component that are connected through Msx2 and Foxo1, see Figure 7. Indeed, previous experimental work established that these genes and TFs have important functions in hematopoiesis: Ccnd2 as a target of Elf5 plays an important role in development and differentiation (Escamilla-Hernández et al., 2010); Pparg is a regulator of hematopoietic stem cell homeostasis (Sertorio et al., 2017); Ihh programs developing mesoderm cells to become hematopoietic or vascular cells (Sugiyama et al., 2011), and suppression of Foxo1 exhibits myeloid lineage expansion and lymphoid developmental abnormalities (Tóthová et al., 2007).

### 3.2 Dataset 2: Blood Stem Cell Differentiation Along Multiple Lineages

**FIGURE 8:**
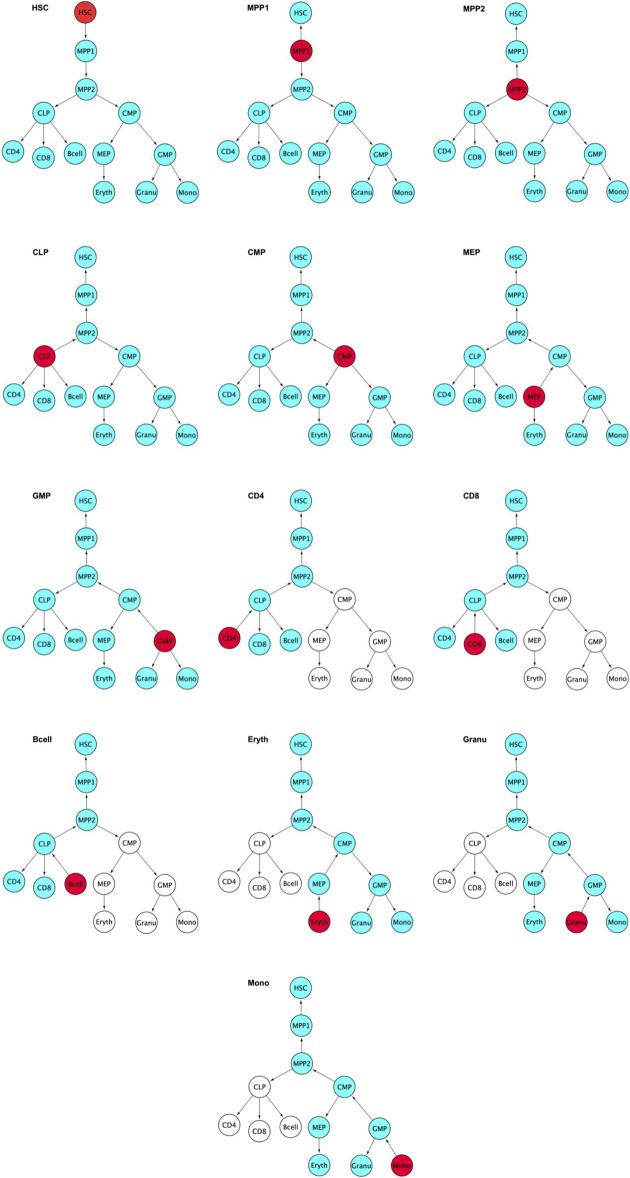
Red colored nodes denote the gene modules whose expression pattern are the highest among the stages in the blood differentiation. Blue color nodes stand for the genes whose expression pattern are lower than the red color nodes. The parent nodes above the red colored node show a gradual increase in the expression pattern and the daughter blue nodes show a gradual decrease which reaches minimal expression at the terminally differentiated cells. Arrows point in the direction of decreasing expression level.White color nodes are the cells whose expression levels are not considered for this stage. The rules are listed in detail in the Appendix:

**FIGURE 9:**
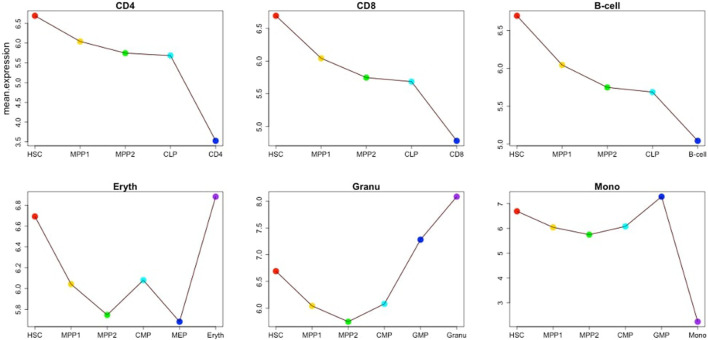
Mean expression of stage-specific genes for the cells in each lineage for the six lineages CD4 T-cell, CD8 T-cell, B cell, erythrocyte, granulocyte and monocyte.

**FIGURE 10:**
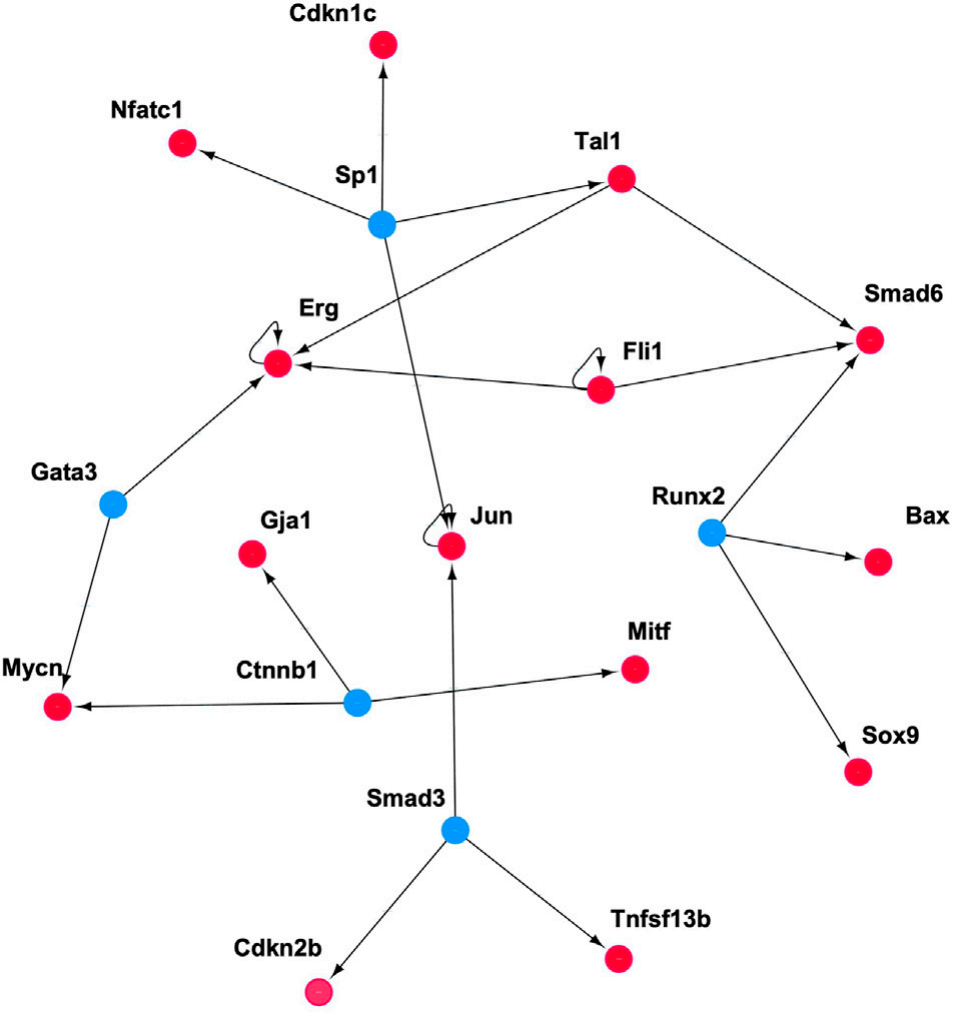
The set of genes and TFs involved in the regulatory pathway for CD8 T-cells. Influencers (red nodes) are the target genes that are regulated by more than five TFs. Connectors are TFs (blue nodes).

The previous section focused on a single example of cellular differentiation in blood formation, starting from previously characterized “key” transcription factors. Therefore, we next expanded our initial approach and applied our concept of “key” expression profiles to a more complex dataset, consisting of six differentiation lineages starting at mouse blood stem cells (Bock et al., 2012). Differentiation of these lineages was shown by the authors to follow a gradual path of changing expression profiles through up to six steps into a fully differentiated cell (Figure 8). To derive the developmental genes and TFs we not only relied on the cell-specific expression pattern as outlined above, but also exploited the computational model and the rules suggested by (Artyomov et al., 2010). Within this model, each cell is defined by two network layers representing expression and epigenetic states. A set of master regulators define the cellular identity. On the event of cellular differentiation, the activated gene module suppresses the activity of the competitor cells either in relationship of parent cell or daughter branch cells. We modified the rules to the extent that developmental regulators specific to each cell state have superiority in terms of gene expression level over neighboring stages while following the cell-specific expression pattern from the top of the hierarchy until terminally differentiated cells. The afore-mentioned patterns led to the identification of between 4 and 128 cell-stage specific genes for the different cell types under consideration (Supplementary Table S21), including several well-known TFs. Figure 9 represents the changes of mean expression value of constituent cells along the cell lineages starting from HSC until a terminally differentiated cell type (e.g. CD4 T-cell, CD8 T-cell, B cell, Erythrocyte (Eryth), Granulocyte (Granu) or Monocyte (Mono)). The stage-specific genes of erythrocytes and granulocytes have particularly high expression levels in the terminally differentiated stage. For CD4 T-cells, CD8 T-cells, B cells, and monocytes, an inverse trend is observed. Supplementary Table S22 shows the number of lineage-specific correlated genes including the involved TFs. Additionally, it depicts the number of TFs that regulate the correlated genes inferred from the TRRUST database and the number of identified correlated genes that are targets of these TFs. Supplementary Tables S23–S28 contain the GO terms and KEGG pathways for the set of TFs that regulate the correlated genes mentioned in the second layer. GO terms such as GO:0045165, GO: 0001709, GO:0001708 annotated to cell fate commitment, cell fate determination and cell fate specification have been identified in the downstream analysis of almost all the lineage-specific TFs. Supplementary Table S29 shows the network statistics for the six lineages. As mentioned before, these networks consist of the derived TFs in the third layer and the target genes of the second layer. The network size lies between 81 and 272 nodes having 66 up to 293 interactions. Figure 10 illustrates the CD8 network constructed by the TFs and their target genes that overlap with the correlated genes in the second layer. The PathDevFate program highlighted genes (influencers colored red and connectors colored blue) that reside along the path to connect the influencers. Supplementary Tables S30–S35 list these nodes for the six lineages including their roles and in-degree and out-degree. Supplementary Table S36 displays the enriched GO terms and KEGG pathways for the set of genes and TFs involved in the regulatory pathway of the CD8 T-cell lineage. Among many terms related to cell differentiation and cell fate, GO: 0030217, which is annotated to the three involved genes Gata3, Ctnnb1 and Runx2, stands for T cell differentiation. Possible validation experiments of our predictions would be CRISPR-Cas knock out of these genes or siRNA silencing. Our expectation is that this would impair differentiation. Jun, Gata3, Nfatc1 and Runx2 are known to be key TFs for memory CD8 T-cell development based on a genome-wide regulatory network (Hu and Chen, 2013). Fli1, Smad3, Sp1, Mycn, and Tal1 play important roles in CD8 T-cell differentiation and development and in forging T-lymphocyte identity (He et al., 2016; Rothenberg et al., 2016). Supplementary Tables S37–S40 represent the enriched GO terms and KEGG pathways associated with the set of genes and TFs involved in the regulatory pathways of CD4 T-cell, B cell, erythrocyte and granulocyte lineages (Bock et al., 2012).

## 4 Discussion

In this work, we devised a pipeline for inferring a set of genes and TFs that drive the blood differentiation process controlling the cell fate decisions across a lineage starting at the stem cell stage leading to a terminally differentiated stage. We started by identifying a set of genes and TFs having a particular stage-specific developmental expression pattern. PathDevFate is a new method based on the biological observations of (Bock et al., 2012). We first retrieve the expression level of a given set of global regulators across a developmental lineage. By averaging these, we define a “stage-specific pattern”. Before arriving at our pattern definition, we also experimented with a “loosened” criterion where a stage-specific gene could e.g. violate one out of six conditions. But this led to a large increase in the number of identified genes, which confused their downstream analysis. As cell fate regulators, we consider those genes that adhere exactly to the given expression pattern of stage-specific genes across the lineage and are regulated and connected by a set of TFs. Other techniques such as Spearman correlation or the method introduced by (Pavlidis and Noble, 2001) may identify genes where the expression does not peak in the specified stage, but that have optimal matches for the other stages. After determining stage-specific pattern, we identify further genes having highly correlated expression profiles with this pattern and term them “stage-specific” genes and TFs. The stage-specific genes which follow the cell-specific pattern have different expression levels. Therefore, we consider the mean expression of all stage-specific genes as a representative value of all the genes with the same pattern. “Lineage-specific genes” refer to the set of genes that follow the expression fluctuation of the stage-specific genes in the respective lineage. As described before, a TF-gene regulatory network is reconstructed in layer 4 of the workflow. That comprises of TFs (connectors) and targets (influencers). Here, we selected CD4 T-cell, CD8 T-cell, and B cell lineages to elucidate the main biological roles of the influencers and connectors. The set of TFs is analyzed in Supplementary Tables S23–S25. The enrichment analysis of the set of target genes (influencers) is presented in Supplementary File S2. Based on the analysis, influencers take part mainly in the developmental and differentiation processes, whereas connectors in addition contribute to cell fate commitments. At the top level of this pipeline, we introduce a regulatory pathway in a gene-regulatory network of TFs and target genes taking into account the identified correlated genes and the TFs that regulate them. The regulatory pathway consists of a set of influencers that are regulated by multiple TFs and a set of connector TFs that join them. The quality of this pathway depends on several points: First of all, the correlation threshold is a variable unless only perfectly correlated genes are to be considered. After that, the number of TFs that regulate these genes relies on the database(s) and the type of interaction which can be either experimentally confirmed (though likely not in the particular case investigated here) or predicted, or both. After all, the in-degree threshold for influencers is also a variable. A tighter threshold leads to a lower number of influencers but is not correlated to the size of the regulatory pathway. As shown in Supplementary Figure S2, in the lineages of CD8 T-cells and granulocyte the number of connectors dramatically increases after a certain threshold. This observation indicates that those high-indegree influencers are very distant from each other and the algorithm needs to inject many connectors to join them. In principle, this work divides the identified genes and TFs into two groups. The first group describes the set of TFs that show the stage-specific developmental patterns and have a tendency to reach the terminally differentiated state. The second group contains the set of TFs that regulate the set of genes and TFs which correlate with the lineage-specific expression pattern. The regulatory pathway demonstrates a path that encompasses those correlated genes that are targeted by several TFs. This signifies the necessity of the genes to be involved in the process. Moreover, this pathway introduces a set of TFs to synchronize the activities of these influences in the lineage. At this point, it is not very straight-forward to highlight the most important TFs as the number of TFs that are induced for connectivity highly depends on the number of influencers and the distance that these influencers have from each other in the network.

## 5 Conclusion

In this work, we identified a set of genes and, from within this set, TFs that can be considered as potential biomarkers for the cell fate process during blood formation. To infer these candidates, we took as starting point the expression pattern of previously described global regulators in a blood lineage. Using this data, we identified stage-specific genes that are likely associated with the cellular differentiation based on correlated activity profiles. By combining the cell-specific expression pattern we obtained an integrated pattern specific to each lineage. Inferring the set of correlated genes and TFs that follow the lineage-specific expression pattern and incorporating the TFs that regulate the genes that have high correlation with the integrated pattern led to the identification of a regulatory subnetwork of TFs and their target genes. Nodes in these networks were finally prioritized using a newly developed “regulatory pathway” algorithm to identify high-indegree genes and TFs by adding additional connector TFs. All the nodes that reside along this path are suggested to be of a high priority for network function. Here, the set of TFs is prioritized in four layers. In the first layer, there are TFs that are mainly involved in the cellular differentiation process. The second layer consists of TFs that follow the integrated pattern of stage-specific expression pattern. TFs that regulate the correlated genes and TFs in the second layer constitute the candidate TFs in the third layer. Finally, the TFs that cooperatively regulate targets genes and connect high-indegree nodes (influencers) in the network of TFs in the third layer and the correlated genes and TFs in the second layer make up the candidates in the fourth layer. Enrichment analysis demonstrates that these biomarkers are not only involved in determining cell fate but also in other developmental processes such as multicellular organism development etc. KEGG pathway analysis shows that these biomarkers can be potential targets for disease-related biomarkers, such as leukaemia In addition to the computational approach to identify a regulatory pathway driving blood differentiation and also a set of genes and TFs that are introduced in four layers as potential biomarkers, the PathDevFate code can be used as a software to find the shortest path between a set of influencer nodes in the largest connected component where a user can set a threshold for the number of incoming edges. Also, users who want to apply a different ranking scheme can easily modify the provided R scripts and study the data sets of their choice.

## Data Availability

Only publicly available expression datasets were analyzed in this study. The source codes developed for this work are available here: https://github.com/ikmb/KeyDevelopmentalFate.

## Appendix

HSC = (HSC > MPP1) & (MPP1 > MPP2) & (MPP2 > CLP) & (CLP > CD4) & (CLP > CD8) & (CLP > B cell) & (MPP2 > CMP) & (CMP > GMP) & (CMP > MEP) & (MEP > Eryth) & (GMP > Granu) & (GMP > Mono) MPP1 = (HSC < MPP1) & (MPP1 > MPP2) & (MPP2 > CLP) & (CLP > CD4) & (CLP > CD8) & (CLP > B cell) & (MPP2 > CMP) & (CMP > GMP) & (CMP > MEP) & (MEP > Eryth) & (GMP > Granu) & (GMP > Mono)MPP2 = (HSC < MPP1) & (MPP1 < MPP2) & (MPP2 > CLP) & (CLP > CD4) & (CLP > CD8) & (CLP > B cell) & (MPP2 > CMP) & (CMP > GMP) & (CMP > MEP) & (MEP > Eryth) & (GMP > Granu) & (GMP > Mono)CLP = (HSC < MPP1) & (MPP1 < MPP2) & (MPP2 < CLP) & (CLP > CD4) & (CLP > CD8) & (CLP > B cell) & (MPP2 > CMP) & (CMP > GMP) & (CMP > MEP) & (MEP > Eryth) & (GMP > Granu) & (GMP > Mono)CMP = (HSC < MPP1) & (MPP1 < MPP2) & (MPP2 > CLP) & (CLP > CD4) & (CLP > CD8) & (CLP > B cell) & (MPP2 < CMP) & (CMP > GMP) & (CMP > MEP) & (MEP > Eryth) & (GMP > Granu) & (GMP > Mono)MEP = (HSC < MPP1) & (MPP1 < MPP2) & (MPP2 > CLP) & (CLP > CD4) & (CLP > CD8) & (CLP > B cell) & (MPP2 < CMP) & (CMP > GMP) & (CMP < MEP) & (MEP > Eryth) & (GMP > Granu) & (GMP > Mono)GMP = (HSC < MPP1) & (MPP1 < MPP2) & (MPP2 > CLP) & (CLP > CD4) & (CLP > CD8) & (CLP > B cell) & (MPP2 < CMP) & (CMP < GMP) & (CMP > MEP) & (MEP > Eryth) & (GMP > Granu) & (GMP > Mono)CD4 = (HSC < MPP1) & (MPP1 < MPP2) & (MPP2 < CLP) & (CLP < CD4) & (CLP > CD8) & (CLP > B cell) CD8 = (HSC < MPP1) & (MPP1 < MPP2) & (MPP2 < CLP) & (CLP > CD4) & (CLP < CD8) & (CLP > B cell)B cell = (HSC < MPP1) & (MPP1 < MPP2) & (MPP2 < CLP) & (CLP > CD4) & (CLP > CD8) & (CLP < B cell)Eryth = (HSC < MPP1) & (MPP1 < MPP2) & (MPP2 < CMP) & (CMP > GMP) & (CMP < MEP) & (MEP < Eryth) & (GMP > Granu) & (GMP > Mono)Granu = (HSC < MPP1) & (MPP1 < MPP2) & (MPP2 < CMP) & (CMP < GMP) & (CMP > MEP) & (MEP > Eryth) & (GMP < Granu) & (GMP > Mono)Mono = (HSC < MPP1) & (MPP1 < MPP2) & (MPP2 < CMP) & (CMP < GMP) & (CMP > MEP) & (MEP > Eryth) & (GMP > Granu) & (GMP < Mono)
