# Supplementary material for: Identification of Biomarkers Controlling Cell Fate In Blood Cell Development
Source: Front Bioinform. 2021 Jul 19;1:653054. doi: 10.3389/fbinf.2021.653054 (PMC9581055; doi:10.3389/fbinf.2021.653054)
Supplement: Supplementary file 1 [file DataSheet1.PDF]

Identification of Biomarkers Driving Blood Cell Development Controlling Cell Fate

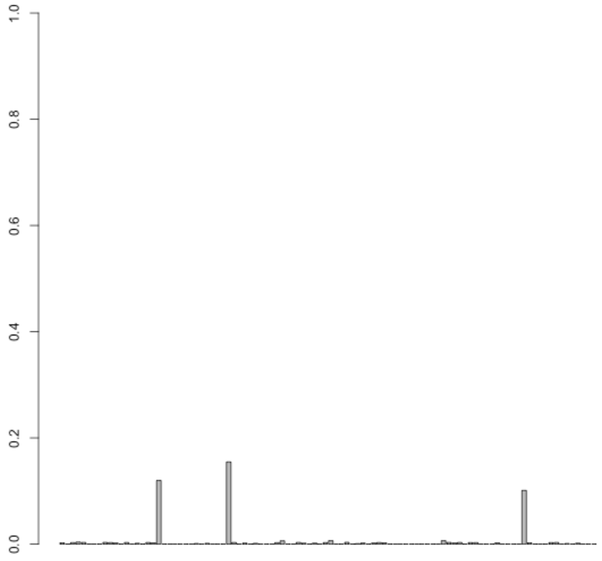

Figure S1. The barplot displays 1000 resampled data on the x-axis and the y-axis demonstrates the percentage of overlapped genes between the correlated genes (mentioned in Table S18) and resampled data.

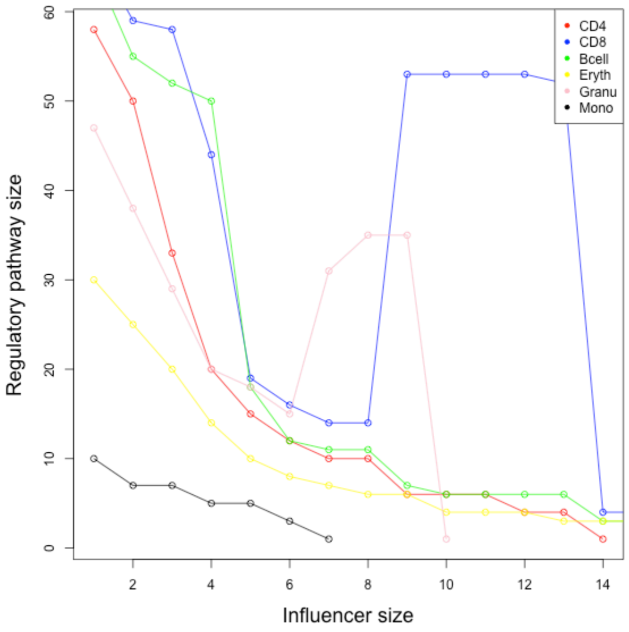

Figure S2. Size of the regulatory pathway for the six lineages based on the threshold for the in-degree of influencers in the network. The regulatory pathway size represents the number of genes and TFs in the connected regulatory pathway that PathDevFate constructs, where the influencer size stands for the lower threshold of in-degree of the influencers, which indicates that all the influencers in the regulatory pathway have in-degree higher than the specified threshold.

Table S1. Number of cell-specific correlated genes including the number of TFs.

Cell	# Correlated genes (# included TFs)
ESC	332 (22)
MES	330 (18)
HB	692 (57)
HE	500 (29)
HP	197 (21)
MAC	308 (10)

Table S2. Enriched GO terms with adjusted p-values < 0.05 for the identified genes in the ESC stage. P-values were adjusted for multiple testing using the BH procedure.

Enriched terms	count	adj. p-values
GO:0007275 multicellular organism development	41	0
GO:0030154 cell differentiation	35	0

Table S3. Enriched GO terms with adjusted p-values < 0.05 for the identified genes in the MES stage. P-values were adjusted for multiple testing using the BH procedure.

Enriched terms	count	adj. p-values
GO:0016055 Wnt signaling pathway	14	0.032

Table S4. Enriched GO terms with adjusted p-values < 0.05 for the identified genes in the HB stage. P-values were adjusted for multiple testing using the BH procedure.

Enriched terms	count	adj. p-values
GO:0006351 transcription, DNA-templated	129	0
GO:0006355 regulation of transcription, DNA-templated	145	0
GO:0008380 RNA splicing	31	0
GO:0007275 multicellular organism development	74	0
GO:0045944 positive regulation of transcription from RNA polymerase II promoter	72	0
GO:0006397 mRNA processing	35	0
GO:0000122 negative regulation of transcription from RNA polymerase II promoter	57	0
GO:0006334 nucleosome assembly	16	0.001
GO:0007411 axon guidance	19	0.001
GO:0009887 organ morphogenesis	16	0.001
GO:0060021 palate development	14	0.001
GO:0007049 cell cycle	44	0.001
GO:0042475 odontogenesis of dentin-containing tooth	12	0.002
GO:0090090 negative regulation of canonical Wnt signaling pathway	15	0.002
GO:0007507 heart development	25	0.002
GO:0051301 cell division	31	0.002
GO:0045892 negative regulation of transcription, DNA-templated	41	0.002
GO:0001707 mesoderm formation	9	0.003
GO:0030513 positive regulation of BMP signaling pathway	9	0.003
GO:0042476 odontogenesis	8	0.003
GO:0002053 positive regulation of mesenchymal cell proliferation	9	0.004
GO:0008285 negative regulation of cell proliferation	30	0.006
GO:0030154 cell differentiation	49	0.006
GO:0048557 embryonic digestive tract morphogenesis	7	0.006
GO:0060325 face morphogenesis	9	0.006
GO:0042733 embryonic digit morphogenesis	11	0.007
GO:0006366 transcription from RNA polymerase II promoter	16	0.007
GO:0045893 positive regulation of transcription, DNA-templated	39	0.007
GO:0003151 outflow tract morphogenesis	10	0.007
GO:0060430 lung saccule development	5	0.008
GO:0045669 positive regulation of osteoblast differentiation	11	0.008
GO:0030509 BMP signaling pathway	12	0.014
GO:0007067 mitotic nuclear division	23	0.016
GO:0006357 regulation of transcription from RNA polymerase II promoter	29	0.017
GO:0001947 heart looping	10	0.018
GO:0050679 positive regulation of epithelial cell proliferation	11	0.018
GO:0030326 embryonic limb morphogenesis	10	0.018
GO:0003281 ventricular septum development	8	0.019
GO:0016569 covalent chromatin modification	22	0.02
GO:0003007 heart morphogenesis	10	0.02
GO:0030901 midbrain development	8	0.023
GO:0060512 prostate gland morphogenesis	4	0.024
GO:0007368 determination of left/right symmetry	11	0.025
GO:0001649 osteoblast differentiation	13	0.027
GO:0001843 neural tube closure	12	0.027
GO:0007399 nervous system development	27	0.029
GO:0010628 positive regulation of gene expression	28	0.029
GO:0001837 epithelial to mesenchymal transition	7	0.03
GO:0050680 negative regulation of epithelial cell proliferation	10	0.039
GO:0021637 trigeminal nerve structural organization	4	0.04
GO:0007059 chromosome segregation	11	0.045
GO:0010718 positive regulation of epithelial to mesenchymal transition	7	0.046

Table S5. Enriched GO terms with adjusted p-values < 0.05 for the identified genes in the HE stage. P-values were adjusted for multiple testing using the BH procedure.

Enriched terms	count	adj. p-values
GO:0007275 multicellular organism development	59	0
GO:0048704 embryonic skeletal system morphogenesis	11	0.001
GO:0006355 regulation of transcription, DNA-templated	90	0.001
GO:0007156 homophilic cell adhesion via plasma membrane adhesion molecules	16	0.005
GO:0006351 transcription, DNA-templated	72	0.034
GO:0035556 intracellular signal transduction	24	0.037
GO:0045893 positive regulation of transcription, DNA-templated	30	0.044
GO:0001525 angiogenesis	17	0.046
GO:0016055 Wnt signaling pathway	16	0.046

Table S6. Enriched GO terms (top) and KEGG pathways (bottom lines starting with mmu...) with adjusted p-values < 0.05 for the identified TFs in the ESC stage. P-values were adjusted for multiple testing using the BH procedure.

Enriched terms	count	adj. p-values
GO:0006355 regulation of transcription, DNA-templated	20	4.188E-13
GO:0006351 transcription, DNA-templated	18	1.292E-11
GO:0045893 positive regulation of transcription, DNA-templated	11	2.927E-8
GO:0045944 positive regulation of transcription from RNA polymerase II promoter	12	2.410E-7
GO:0045892 negative regulation of transcription, DNA-templated	9	1.017E-5
GO:0043586 tongue development	3	0.006
GO:0000122 negative regulation of transcription from RNA polymerase II promoter	7	0.006
GO:0048863 stem cell differentiation	3	0.029
GO:0006357 regulation of transcription from RNA polymerase II promoter	5	0.031
GO:0007275 multicellular organism development	7	0.032
GO:0007492 endoderm development	3	0.032
GO:0030154 cell differentiation	6	0.047
mmu04550:Signaling pathways regulating pluripotency of stem cells	5	3.449E-4

Table S7. Enriched GO terms with adjusted p-values < 0.05 for the identified TFs in the MES stage.

Enriched terms	count	adj. p-values
GO:0006355 regulation of transcription, DNA-templated	17	1.227E-12
GO:0006351 transcription, DNA-templated	15	2.652E-10
GO:0045944 positive regulation of transcription from RNA polymerase II promoter	12	4.734E-9
GO:0045893 positive regulation of transcription, DNA-templated	8	2.258E-5
GO:0006357 regulation of transcription from RNA polymerase II promoter	7	4.531E-5
GO:0030154 cell differentiation	7	0.001
GO:0010468 regulation of gene expression	5	0.005
GO:0000122 negative regulation of transcription from RNA polymerase II promoter	6	0.012
GO:0001569 patterning of blood vessels	3	0.019
GO:0008284 positive regulation of cell proliferation	5	0.033
GO:0042475 odontogenesis of dentin-containing tooth	3	0.039
GO:0007275 multicellular organism development	6	0.039

Table S8: Enriched GO terms (top) and KEGG pathways (bottom lines starting with mmu...) with adjusted p-values < 0.05 for the identified TFs in the HB stage.

Enriched terms	count	adj. p-values
GO:0006351 transcription, DNA-templated	46	1.681E-33
GO:0006355 regulation of transcription, DNA-templated	48	2.008E-33
GO:0000122 negative regulation of transcription from RNA polymerase II promoter	31	7.593E-26
GO:0045944 positive regulation of transcription from RNA polymerase II promoter	31	5.307E-22
GO:0045892 negative regulation of transcription, DNA-templated	26	2.177E-21
GO:0045893 positive regulation of transcription, DNA-templated	21	2.053E-14
GO:0007275 multicellular organism development	21	5.637E-10
GO:0042733 embryonic digit morphogenesis	9	2.194E-9
GO:0006357 regulation of transcription from RNA polymerase II promoter	14	1.061E-8
GO:0003151 outflow tract morphogenesis	8	2.178E-8
GO:0030513 positive regulation of BMP signaling pathway	7	8.700E-8
GO:0010629 negative regulation of gene expression	11	3.997E-7
GO:0030326 embryonic limb morphogenesis	7	2.417E-6
GO:0060021 palate development	7	1.375E-5
GO:0010628 positive regulation of gene expression	11	1.513E-5
GO:0030154 cell differentiation	14	1.910E-5
GO:0042476 odontogenesis	5	7.168E-5
GO:0035116 embryonic hindlimb morphogenesis	5	1.558E-4
GO:0006366 transcription from RNA polymerase II promoter	7	1.769E-4
GO:0008284 positive regulation of cell proliferation	11	1.828E-4
GO:0035115 embryonic forelimb morphogenesis	5	1.910E-4
GO:0051216 cartilage development	6	2.069E-4
GO:0043066 negative regulation of apoptotic process	11	2.334E-4
GO:0030509 BMP signaling pathway	6	2.540E-4
GO:0003198 epithelial to mesenchymal transition involved in endocardial cushion formation	4	2.957E-4
GO:0001843 neural tube closure	6	3.999E-4
GO:0045599 negative regulation of fat cell differentiation	5	4.780E-4
GO:0008584 male gonad development	6	7.478E-4
GO:0001701 in utero embryonic development	8	9.687E-4
GO:0071560 cellular response to transforming growth factor beta stimulus	5	0.001
GO:0007219 Notch signaling pathway	6	0.001
GO:0001947 heart looping	5	0.001
GO:0003007 heart morphogenesis	5	0.001
GO:0023019 signal transduction involved in regulation of gene expression	4	0.001
GO:0003148 outflow tract septum morphogenesis	4	0.001
GO:0043433 negative regulation of sequence-specific DNA binding transcription factor activity	5	0.001
GO:0045662 negative regulation of myoblast differentiation	4	0.001
GO:0060512 prostate gland morphogenesis	3	0.002
GO:0021983 pituitary gland development	4	0.002
GO:0001837 epithelial to mesenchymal transition	4	0.002
GO:0061312 BMP signaling pathway involved in heart development	3	0.003
GO:0010718 positive regulation of epithelial to mesenchymal transition	4	0.003
GO:0001707 mesoderm formation	4	0.003
GO:0055007 cardiac muscle cell differentiation	4	0.004
GO:0003180 aortic valve morphogenesis	3	0.004
GO:0030879 mammary gland development	4	0.004
GO:0001656 metanephros development	4	0.004
GO:0060412 ventricular septum morphogenesis	4	0.004
GO:0003281 ventricular septum development	4	0.004
GO:0030901 midbrain development	4	0.005
GO:0060430 lung saccule development	3	0.005
GO:0031018 endocrine pancreas development	4	0.005

GO:0001501 skeletal system development	5	0.006
GO:0045597 positive regulation of cell differentiation	4	0.007
GO:0001649 osteoblast differentiation	5	0.007
GO:0045843 negative regulation of striated muscle tissue development	3	0.007
GO:0048511 rhythmic process	5	0.010
GO:1902894 negative regulation of pri-miRNA transcription from RNA polymerase II promoter	3	0.010
GO:0048704 embryonic skeletal system morphogenesis	4	0.011
GO:2000678 negative regulation of transcription regulatory region DNA binding	3	0.012
GO:0030903 notochord development	3	0.012
GO:0003215 cardiac right ventricle morphogenesis	3	0.012
GO:0048715 negative regulation of oligodendrocyte differentiation	3	0.014
GO:0007399 nervous system development	7	0.014
GO:0042475 odontogenesis of dentin-containing tooth	4	0.015
GO:0061029 eyelid development in camera-type eye	3	0.015
GO:0008285 negative regulation of cell proliferation	7	0.015
GO:0071363 cellular response to growth factor stimulus	4	0.015
GO:0007507 heart development	6	0.016
GO:2000679 positive regulation of transcription regulatory region DNA binding	3	0.016
GO:0003323 type B pancreatic cell development	3	0.016
GO:0001706 endoderm formation	3	0.016
GO:0061036 positive regulation of cartilage development	3	0.016
GO:0045669 positive regulation of osteoblast differentiation	4	0.017
GO:0016569 covalent chromatin modification	6	0.017
GO:0003203 endocardial cushion morphogenesis	3	0.018
GO:0050680 negative regulation of epithelial cell proliferation	4	0.019
GO:0045165 cell fate commitment	4	0.019
GO:0007179 transforming growth factor beta receptor signaling pathway	4	0.021
GO:0060065 uterus development	3	0.021
GO:0050679 positive regulation of epithelial cell proliferation	4	0.021
GO:0006338 chromatin remodeling	4	0.024
GO:0048557 embryonic digestive tract morphogenesis	3	0.025
GO:0010468 regulation of gene expression	6	0.029
GO:0030097 hemopoiesis	4	0.029
GO:0060045 positive regulation of cardiac muscle cell proliferation	3	0.029
GO:0035050 embryonic heart tube development	3	0.029
GO:0045778 positive regulation of ossification	3	0.029
GO:0060425 lung morphogenesis	3	0.031
GO:0043044 ATP-dependent chromatin remodeling	3	0.033
GO:0048844 artery morphogenesis	3	0.033
GO:0001503 ossification	4	0.038
GO:0043065 positive regulation of apoptotic process	6	0.039
GO:0042493 response to drug	6	0.041
GO:0048738 cardiac muscle tissue development	3	0.042
GO:0043392 negative regulation of DNA binding	3	0.042
GO:0090090 negative regulation of canonical Wnt signaling pathway	4	0.043
GO:0045666 positive regulation of neuron differentiation	4	0.043
GO:0007623 circadian rhythm	4	0.049
GO:0043967 histone H4 acetylation	3	0.049
GO:0009887 organ morphogenesis	4	0.049
mmu04350:TGF-beta signaling pathway	6	6.052E-4
mmu04550:Signaling pathways regulating pluripotency of stem cells	6	0.003
mmu05217:Basal cell carcinoma	4	0.017

Table S9. Enriched GO terms with adjusted p-values < 0.05 for the identified TFs in the HE stage.

Enriched terms	count	adj. p-values
GO:0006351 transcription, DNA-templated	24	2.020E-17
GO:0006355 regulation of transcription, DNA-templated	25	1.349E-17
GO:0045944 positive regulation of transcription from RNA polymerase II promoter	15	1.724E-9
GO:0007275 multicellular organism development	14	3.698E-8
GO:0000122 negative regulation of transcription from RNA polymerase II promoter	11	2.945E-6
GO:0051216 cartilage development	6	8.053E-6
GO:0045893 positive regulation of transcription, DNA-templated	9	6.338E-5
GO:0006357 regulation of transcription from RNA polymerase II promoter	7	9.984E-4
GO:0060948 cardiac vascular smooth muscle cell development	3	0.001
GO:0003151 outflow tract morphogenesis	4	0.002
GO:0048704 embryonic skeletal system morphogenesis	4	0.002
GO:0001947 heart looping	4	0.003
GO:0003184 pulmonary valve morphogenesis	3	0.003
GO:1902895 positive regulation of pri-miRNA transcription from RNA polymerase II promoter	3	0.014
GO:0090103 cochlea morphogenesis	3	0.015
GO:0009952 anterior/posterior pattern specification	4	0.015

Table S10. Enriched GO terms (top) and KEGG pathways (bottom lines starting with mmu...) with adjusted p-values < 0.05 for the identified TFs in the HP stage.

Enriched terms	count	adj. p-values
GO:0006351 transcription, DNA-templated	16	1.171E-9
GO:0006355 regulation of transcription, DNA-templated	16	9.302E-9
GO:0045944 positive regulation of transcription from RNA polymerase II promoter	12	5.694E-8
GO:0006357 regulation of transcription from RNA polymerase II promoter	7	1.470E-4
GO:0000122 negative regulation of transcription from RNA polymerase II promoter	7	0.003
GO:0016569 covalent chromatin modification	5	0.006
GO:0007548 sex differentiation	3	0.008
GO:0019915 lipid storage	3	0.011
GO:0040014 regulation of multicellular organism growth	3	0.020
GO:0045599 negative regulation of fat cell differentiation	3	0.028
GO:0032922 circadian regulation of gene expression	3	0.042
mmu05202:Transcriptional misregulation in cancer	4	0.011
mmu05221:Acute myeloid leukemia	3	0.013
mmu04919:Thyroid hormone signaling pathway	3	0.036

Table S11. Enriched GO terms with adjusted p-values < 0.05 for the identified TFs in the MAC stage.

Enriched terms	count	adj. p-values
GO:0006351 transcription, DNA-templated	10	2.529E-7
GO:0006355 regulation of transcription, DNA-templated	10	7.005E-7
GO:0000122 negative regulation of transcription from RNA polymerase II promoter	5	0.016

Table S12: Enriched GO terms (top) with adjusted p-values < 0.05 for the identified targets in the ESC stage.

Enriched terms	count	adj. p-values
GO:0045944 positive regulation of transcription from RNA polymerase II promoter	48	1.2e-25
GO:0045893 positive regulation of transcription, DNA-templated	32	2.32e-17
GO:0008284 positive regulation of cell proliferation	28	6.76e-14
GO:0000122 negative regulation of transcription from RNA polymerase II promoter	30	8.11e-13
GO:0006355 regulation of transcription, DNA-templated	50	8.11e-13
GO:0042127 regulation of cell proliferation	19	1.77e-12
GO:0006351 transcription, DNA-templated	45	1.86e-12
GO:0010628 positive regulation of gene expression	22	2.52e-11
GO:0043066 negative regulation of apoptotic process	25	3.59e-11
GO:0007275 multicellular organism development	30	1.91e-09
GO:0010468 regulation of gene expression	18	2.36e-09
GO:0042493 response to drug	18	9.75e-09
GO:0048661 positive regulation of smooth muscle cell proliferation	11	1.38e-08
GO:0045892 negative regulation of transcription, DNA-templated	22	1.67e-08
GO:0010629 negative regulation of gene expression	16	2.11e-08
GO:0045666 positive regulation of neuron differentiation	11	1.42e-07
GO:0030324 lung development	11	7.77e-07
GO:0031018 endocrine pancreas development	8	7.98e-07
GO:0010942 positive regulation of cell death	8	2.25e-06
GO:0071407 cellular response to organic cyclic compound	9	2.59e-06
GO:0048863 stem cell differentiation	7	6.51e-06
GO:0030154 cell differentiation	21	1.01e-05
GO:0002053 positive regulation of mesenchymal cell proliferation	7	1.06e-05
GO:0008285 negative regulation of cell proliferation	15	1.44e-05
GO:0050679 positive regulation of epithelial cell proliferation	8	4.38e-05
GO:0009749 response to glucose	8	5.03e-05
GO:0007050 cell cycle arrest	8	6.28e-05
GO:0045766 positive regulation of angiogenesis	9	7.71e-05
GO:0043586 tongue development	5	9.98e-05
GO:0043065 positive regulation of apoptotic process	13	0.00011
GO:0001934 positive regulation of protein phosphorylation	10	0.000182
GO:0048839 inner ear development	7	0.000244
GO:0032496 response to lipopolysaccharide	10	0.000299
GO:0051091 positive regulation of sequence-specific DNA binding transcription factor activity	8	0.000325
GO:0045165 cell fate commitment	7	0.000371
GO:0051726 regulation of cell cycle	8	0.000416
GO:0045596 negative regulation of cell differentiation	8	0.000455
GO:0045665 negative regulation of neuron differentiation	7	0.000468
GO:0045597 positive regulation of cell differentiation	6	0.000527
GO:0043010 camera-type eye development	7	0.000531
GO:0060441 epithelial tube branching involved in lung morphogenesis	5	0.000713
GO:0048646 anatomical structure formation involved in morphogenesis	5	0.000713
GO:0045785 positive regulation of cell adhesion	6	0.0013
GO:0031100 organ regeneration	6	0.0013
GO:0070374 positive regulation of ERK1 and ERK2 cascade	9	0.00133
GO:0042060 wound healing	7	0.00137
GO:0097421 liver regeneration	5	0.00141
GO:0051897 positive regulation of protein kinase B signaling	7	0.00157
GO:0000082 G1/S transition of mitotic cell cycle	6	0.00195
GO:0030335 positive regulation of cell migration	9	0.00196
GO:0006357 regulation of transcription from RNA polymerase II promoter	12	0.00196
GO:0000060 protein import into nucleus, translocation	5	0.00198
GO:0033327 Leydig cell differentiation	4	0.00199
GO:0048146 positive regulation of fibroblast proliferation	6	0.00221

GO:0010033 response to organic substance	6	0.00221
GO:0006919 activation of cysteine-type endopeptidase activity involved in apoptotic process	6	0.00247
GO:0007623 circadian rhythm	7	0.00248
GO:0045669 positive regulation of osteoblast differentiation	6	0.0026
GO:0007420 brain development	9	0.00265
GO:0030198 extracellular matrix organization	7	0.00306
GO:0007224 smoothened signaling pathway	6	0.00308
GO:0007263 nitric oxide mediated signal transduction	4	0.00351
GO:0051384 response to glucocorticoid	6	0.0038
GO:0033280 response to vitamin D	4	0.00415
GO:0048565 digestive tract development	5	0.00453
GO:0071285 cellular response to lithium ion	4	0.00486
GO:0034097 response to cytokine	6	0.00513
GO:0048511 rhythmic process	7	0.00516
GO:0045429 positive regulation of nitric oxide biosynthetic process	5	0.00549
GO:0051781 positive regulation of cell division	5	0.00549
GO:2000648 positive regulation of stem cell proliferation	4	0.00554
GO:0035019 somatic stem cell population maintenance	5	0.0059
GO:0042593 glucose homeostasis	7	0.00599
GO:0050796 regulation of insulin secretion	5	0.00614
GO:0044849 estrous cycle	4	0.00617
GO:0001666 response to hypoxia	8	0.00639
GO:0006366 transcription from RNA polymerase II promoter	7	0.0069
GO:0048468 cell development	5	0.00693
GO:0042981 regulation of apoptotic process	8	0.00763
GO:0043401 steroid hormone mediated signaling pathway	5	0.00913
GO:0043406 positive regulation of MAP kinase activity	5	0.00932
GO:0006954 inflammatory response	10	0.00933
GO:0001503 ossification	6	0.0094
GO:0060850 regulation of transcription involved in cell fate commitment	3	0.00943
GO:0090131 mesenchyme migration	3	0.00943
GO:0001714 endodermal cell fate specification	3	0.00943
GO:0001708 cell fate specification	4	0.00943
GO:0071222 cellular response to lipopolysaccharide	8	0.00948
GO:0032526 response to retinoic acid	5	0.011
GO:0045672 positive regulation of osteoclast differentiation	4	0.0118
GO:0043410 positive regulation of MAPK cascade	6	0.0123
GO:0002052 positive regulation of neuroblast proliferation	4	0.013
GO:0009887 organ morphogenesis	6	0.0148
GO:0042475 odontogenesis of dentin-containing tooth	5	0.0151
GO:0030326 embryonic limb morphogenesis	5	0.0151
GO:0001701 in utero embryonic development	9	0.0151
GO:0042102 positive regulation of T cell proliferation	5	0.0158
GO:0060754 positive regulation of mast cell chemotaxis	3	0.0166
GO:0021798 forebrain dorsal/ventral pattern formation	3	0.0166
GO:0031622 positive regulation of fever generation	3	0.0166
GO:0060664 epithelial cell proliferation involved in salivary gland morphogenesis	3	0.0166
GO:0001649 osteoblast differentiation	6	0.0167
GO:0002040 sprouting angiogenesis	4	0.0168
GO:0001525 angiogenesis	8	0.0176
GO:0042327 positive regulation of phosphorylation	4	0.0176
GO:0007568 aging	7	0.0176
GO:0043154 negative regulation of cysteine-type endopeptidase activity involved in apoptotic process	5	0.0178
GO:0071346 cellular response to interferon-gamma	5	0.018
GO:0045471 response to ethanol	6	0.0181
GO:2000379 positive regulation of reactive oxygen species metabolic process	4	0.0188
GO:0048663 neuron fate commitment	4	0.0202
GO:0060463 lung lobe morphogenesis	3	0.0203

GO:0030823 regulation of cGMP metabolic process	3	0.0203
GO:0019827 stem cell population maintenance	5	0.0203
GO:0071345 cellular response to cytokine stimulus	4	0.0217
GO:0031016 pancreas development	4	0.0217
GO:0048709 oligodendrocyte differentiation	4	0.0235
GO:0043627 response to estrogen	5	0.0242
GO:0045080 positive regulation of chemokine biosynthetic process	3	0.0245
GO:0034393 positive regulation of smooth muscle cell apoptotic process	3	0.0245
GO:0003417 growth plate cartilage development	3	0.0245
GO:0007507 heart development	8	0.0259
GO:0035115 embryonic forelimb morphogenesis	4	0.0267
GO:0048754 branching morphogenesis of an epithelial tube	4	0.0267
GO:0010469 regulation of receptor activity	3	0.0293
GO:0090399 replicative senescence	3	0.0293
GO:0071347 cellular response to interleukin-1	5	0.0295
GO:0014911 positive regulation of smooth muscle cell migration	4	0.0327
GO:0045595 regulation of cell differentiation	4	0.0327
GO:0030728 ovulation	3	0.0346
GO:0001569 patterning of blood vessels	4	0.0348
GO:0060021 palate development	5	0.0352
GO:0032869 cellular response to insulin stimulus	5	0.0395
GO:0021879 forebrain neuron differentiation	3	0.0402
GO:0042753 positive regulation of circadian rhythm	3	0.0402
GO:0045945 positive regulation of transcription from RNA polymerase III promoter	3	0.0402
GO:0045740 positive regulation of DNA replication	4	0.041
GO:0007411 axon guidance	6	0.0427
GO:0008283 cell proliferation	7	0.0456
GO:0043923 positive regulation by host of viral transcription	3	0.046
GO:0007399 nervous system development	9	0.0461

Table S13: Enriched GO terms (top) with adjusted p-values < 0.05 for the identified targets in the MES stage.

Enriched terms	count	adj. p-values
GO:0045944 positive regulation of transcription from RNA polymerase II promoter	45	6.34e-22
GO:0000122 negative regulation of transcription from RNA polymerase II promoter	36	5.42e-18
GO:0007275 multicellular organism development	40	5.22e-17
GO:0045893 positive regulation of transcription, DNA-templated	31	2.99e-16
GO:0008284 positive regulation of cell proliferation	28	4.22e-14
GO:0006355 regulation of transcription, DNA-templated	52	6.03e-14
GO:0042475 odontogenesis of dentin-containing tooth	14	7.04e-14
GO:0006351 transcription, DNA-templated	46	8.18e-13
GO:0030326 embryonic limb morphogenesis	13	1.76e-12
GO:0045892 negative regulation of transcription, DNA-templated	24	7.69e-10
GO:0030154 cell differentiation	27	1.19e-09
GO:0010628 positive regulation of gene expression	20	2.67e-09
GO:0010468 regulation of gene expression	18	3.15e-09
GO:0035116 embryonic hindlimb morphogenesis	9	4.88e-09
GO:0009952 anterior/posterior pattern specification	12	2.61e-08
GO:0042493 response to drug	17	1.03e-07
GO:0006357 regulation of transcription from RNA polymerase II promoter	18	1.23e-07
GO:0001708 cell fate specification	7	7.32e-07
GO:0030182 neuron differentiation	11	1.24e-06
GO:0021983 pituitary gland development	7	3.55e-06
GO:0008285 negative regulation of cell proliferation	16	3.64e-06
GO:0008584 male gonad development	10	4.94e-06
GO:0055007 cardiac muscle cell differentiation	7	1.22e-05
GO:0007507 heart development	13	1.33e-05

GO:0003007 heart morphogenesis	8	1.64e-05
GO:0042733 embryonic digit morphogenesis	8	1.96e-05
GO:0042127 regulation of cell proliferation	12	2.36e-05
GO:0060045 positive regulation of cardiac muscle cell proliferation	6	2.46e-05
GO:0045165 cell fate commitment	8	3.21e-05
GO:0048536 spleen development	7	3.48e-05
GO:0051091 positive regulation of sequence-specific DNA binding transcription factor activity	9	3.55e-05
GO:0051216 cartilage development	8	7.11e-05
GO:0003151 outflow tract morphogenesis	7	8.39e-05
GO:0001958 endochondral ossification	6	8.51e-05
GO:0030900 forebrain development	8	8.99e-05
GO:0030324 lung development	9	9.1e-05
GO:0060070 canonical Wnt signaling pathway	8	0.000107
GO:0048863 stem cell differentiation	6	0.000141
GO:0001947 heart looping	7	0.000167
GO:0035115 embryonic forelimb morphogenesis	6	0.00018
GO:0045747 positive regulation of Notch signaling pathway	6	0.000202
GO:0001569 patterning of blood vessels	6	0.000293
GO:0042472 inner ear morphogenesis	7	0.000364
GO:0050680 negative regulation of epithelial cell proliferation	7	0.000364
GO:0030890 positive regulation of B cell proliferation	6	0.000402
GO:0045596 negative regulation of cell differentiation	8	0.00045
GO:0045665 negative regulation of neuron differentiation	7	0.000456
GO:0010629 negative regulation of gene expression	11	0.00046
GO:0035019 somatic stem cell population maintenance	6	0.000517
GO:0023019 signal transduction involved in regulation of gene expression	5	0.000575
GO:0003139 secondary heart field specification	4	0.000592
GO:0014807 regulation of somitogenesis	4	0.000592
GO:0007368 determination of left/right symmetry	7	0.000595
GO:0010942 positive regulation of cell death	6	0.000601
GO:0007283 spermatogenesis	13	0.000609
GO:0003148 outflow tract septum morphogenesis	5	0.000623
GO:0007219 Notch signaling pathway	8	0.000686
GO:0030097 hemopoiesis	7	0.00078
GO:0001944 vasculature development	5	0.000807
GO:0009954 proximal/distal pattern formation	5	0.000807
GO:0030509 BMP signaling pathway	7	0.000818
GO:0042476 odontogenesis	5	0.000927
GO:0048538 thymus development	6	0.001
GO:0001541 ovarian follicle development	6	0.00115
GO:0032526 response to retinoic acid	6	0.00115
GO:0006366 transcription from RNA polymerase II promoter	8	0.00117
GO:0001503 ossification	7	0.00129
GO:0048663 neuron fate commitment	5	0.00168
GO:0060349 bone morphogenesis	5	0.00168
GO:0032355 response to estradiol	7	0.0017
GO:0090090 negative regulation of canonical Wnt signaling pathway	7	0.00171
GO:0042640 anagen	4	0.00173
GO:0045666 positive regulation of neuron differentiation	7	0.00178
GO:0071392 cellular response to estradiol stimulus	5	0.00179
GO:0043065 positive regulation of apoptotic process	11	0.0021
GO:0003197 endocardial cushion development	4	0.00211
GO:0003198 epithelial to mesenchymal transition involved in endocardial cushion formation	4	0.00211
GO:0001501 skeletal system development	7	0.00211
GO:0031069 hair follicle morphogenesis	5	0.00214
GO:0030513 positive regulation of BMP signaling pathway	5	0.00237
GO:0043066 negative regulation of apoptotic process	14	0.00247
GO:0048715 negative regulation of oligodendrocyte differentiation	4	0.00249

GO:0019827 stem cell population maintenance	6	0.00268
GO:0048665 neuron fate specification	4	0.00298
GO:0048701 embryonic cranial skeleton morphogenesis	5	0.00305
GO:0045595 regulation of cell differentiation	5	0.00305
GO:0033077 T cell differentiation in thymus	5	0.00305
GO:0045787 positive regulation of cell cycle	5	0.00333
GO:0007281 germ cell development	5	0.00333
GO:0050679 positive regulation of epithelial cell proliferation	6	0.00352
GO:0001701 in utero embryonic development	10	0.00399
GO:0001764 neuron migration	7	0.00407
GO:0051145 smooth muscle cell differentiation	4	0.00488
GO:0002076 osteoblast development	4	0.00488
GO:0002063 chondrocyte development	4	0.00488
GO:0002062 chondrocyte differentiation	5	0.00541
GO:0060021 palate development	6	0.00561
GO:0001502 cartilage condensation	4	0.00652
GO:0001755 neural crest cell migration	5	0.00668
GO:0007417 central nervous system development	6	0.00671
GO:0048557 embryonic digestive tract morphogenesis	4	0.00731
GO:0035162 embryonic hemopoiesis	4	0.00731
GO:0009948 anterior/posterior axis specification	4	0.00833
GO:0010463 mesenchymal cell proliferation	3	0.00887
GO:0007411 axon guidance	7	0.00973
GO:0035914 skeletal muscle cell differentiation	5	0.00975
GO:0048704 embryonic skeletal system morphogenesis	5	0.00975
GO:0060441 epithelial tube branching involved in lung morphogenesis	4	0.0103
GO:0060425 lung morphogenesis	4	0.0103
GO:0001756 somitogenesis	5	0.0103
GO:0007389 pattern specification process	5	0.0116
GO:0007267 cell-cell signaling	6	0.0119
GO:0061312 BMP signaling pathway involved in heart development	3	0.0123
GO:0001782 B cell homeostasis	4	0.0125
GO:0046427 positive regulation of JAK-STAT cascade	4	0.0125
GO:0035108 limb morphogenesis	4	0.0155
GO:0071363 cellular response to growth factor stimulus	5	0.0159
GO:0051726 regulation of cell cycle	6	0.0164
GO:0071300 cellular response to retinoic acid	5	0.0164
GO:0002320 lymphoid progenitor cell differentiation	3	0.0164
GO:0048645 organ formation	3	0.0164
GO:0007399 nervous system development	10	0.0165
GO:0001649 osteoblast differentiation	6	0.0166
GO:0001525 angiogenesis	8	0.0187
GO:0045669 positive regulation of osteoblast differentiation	5	0.0189
GO:0050767 regulation of neurogenesis	4	0.0192
GO:0030856 regulation of epithelial cell differentiation	3	0.0202
GO:0060428 lung epithelium development	3	0.0202
GO:0001568 blood vessel development	5	0.0214
GO:0031016 pancreas development	4	0.0224
GO:0001934 positive regulation of protein phosphorylation	7	0.0236
GO:0060982 coronary artery morphogenesis	3	0.0249
GO:0060290 transdifferentiation	3	0.0249
GO:0070374 positive regulation of ERK1 and ERK2 cascade	7	0.0257
GO:0034504 protein localization to nucleus	4	0.0259
GO:0071773 cellular response to BMP stimulus	4	0.0259
GO:0045786 negative regulation of cell cycle	4	0.0276
GO:0048754 branching morphogenesis of an epithelial tube	4	0.0276
GO:0071260 cellular response to mechanical stimulus	5	0.0284
GO:0043010 camera-type eye development	5	0.0284
GO:0030177 positive regulation of Wnt signaling pathway	4	0.0294
GO:0002053 positive regulation of mesenchymal cell proliferation	4	0.0294

GO:0048661 positive regulation of smooth muscle cell proliferation	5	0.0306
GO:0032496 response to lipopolysaccharide	7	0.0311
GO:0034097 response to cytokine	5	0.0315
GO:0007605 sensory perception of sound	6	0.0343
GO:0048617 embryonic foregut morphogenesis	3	0.0346
GO:0060174 limb bud formation	3	0.0346
GO:0048643 positive regulation of skeletal muscle tissue development	3	0.0346
GO:0045603 positive regulation of endothelial cell differentiation	3	0.0346
GO:0060325 face morphogenesis	4	0.0349
GO:0030217 T cell differentiation	4	0.0372
GO:0009636 response to toxic substance	5	0.0376
GO:0097192 extrinsic apoptotic signaling pathway in absence of ligand	4	0.0392
GO:0021879 forebrain neuron differentiation	3	0.0396
GO:0001823 mesonephros development	3	0.0396
GO:0001974 blood vessel remodeling	4	0.0413
GO:0016055 Wnt signaling pathway	7	0.0422
GO:0048706 embryonic skeletal system development	4	0.0434
GO:0003215 cardiac right ventricle morphogenesis	3	0.0451
GO:0048469 cell maturation	4	0.0455
GO:0030514 negative regulation of BMP signaling pathway	4	0.0477
GO:0008283 cell proliferation	7	0.0479

Table S14: Enriched GO terms (top) with adjusted p-values < 0.05 for the identified targets in the HB stage.

Enriched terms	co unt	adj. p-values
GO:0045944 positive regulation of transcription from RNA polymerase II promoter	103	1.56e-50
GO:0045893 positive regulation of transcription, DNA-templated	77	4.53e-44
GO:0000122 negative regulation of transcription from RNA polymerase II promoter	81	1.01e-40
GO:0008284 positive regulation of cell proliferation	61	9.21e-30
GO:0006355 regulation of transcription, DNA-templated	107	3.4e-22
GO:0010628 positive regulation of gene expression	46	4.12e-22
GO:0006351 transcription, DNA-templated	95	2.39e-21
GO:0008285 negative regulation of cell proliferation	43	4.28e-20
GO:0007275 multicellular organism development	67	1.18e-19
GO:0045892 negative regulation of transcription, DNA-templated	49	3.57e-18
GO:0007507 heart development	33	1.67e-16
GO:0042493 response to drug	36	8.21e-16
GO:0043066 negative regulation of apoptotic process	45	1.5e-15
GO:0030154 cell differentiation	52	3.73e-15
GO:0030324 lung development	23	2.39e-14
GO:0010468 regulation of gene expression	33	2.54e-14
GO:0006357 regulation of transcription from RNA polymerase II promoter	36	1.12e-13
GO:0030326 embryonic limb morphogenesis	17	6.57e-13
GO:0010629 negative regulation of gene expression	29	9.04e-13
GO:0001501 skeletal system development	20	1.77e-12
GO:0001666 response to hypoxia	25	1.84e-12
GO:0042127 regulation of cell proliferation	26	9.28e-12
GO:0050679 positive regulation of epithelial cell proliferation	17	1.25e-11
GO:0001525 angiogenesis	26	2.79e-11
GO:0048469 cell maturation	14	3.06e-11
GO:0045669 positive regulation of osteoblast differentiation	16	3.22e-11
GO:0048754 branching morphogenesis of an epithelial tube	13	3.46e-11
GO:0002062 chondrocyte differentiation	14	3.75e-11
GO:0045165 cell fate commitment	16	7.09e-11
GO:1902895 positive regulation of pri-miRNA transcription from RNA polymerase II promoter	11	7.65e-11
GO:0045666 positive regulation of neuron differentiation	18	1.04e-10
GO:0032496 response to lipopolysaccharide	23	1.63e-10

GO:0051091 positive regulation of sequence-specific DNA binding transcription factor activity	18	1.87e-10
GO:0042733 embryonic digit morphogenesis	15	2.79e-10
GO:0001649 osteoblast differentiation	18	4.39e-10
GO:0045597 positive regulation of cell differentiation	13	6.97e-10
GO:0032355 response to estradiol	17	7.94e-10
GO:0009887 organ morphogenesis	17	2.56e-09
GO:0032332 positive regulation of chondrocyte differentiation	10	2.8e-09
GO:0001503 ossification	16	4.09e-09
GO:0034097 response to cytokine	15	4.37e-09
GO:0051216 cartilage development	15	5.07e-09
GO:0042593 glucose homeostasis	18	5.18e-09
GO:0035116 embryonic hindlimb morphogenesis	11	6.22e-09
GO:0048646 anatomical structure formation involved in morphogenesis	10	6.35e-09
GO:0007219 Notch signaling pathway	17	1.75e-08
GO:0001658 branching involved in ureteric bud morphogenesis	12	1.88e-08
GO:0070374 positive regulation of ERK1 and ERK2 cascade	20	2.15e-08
GO:0001889 liver development	15	3.33e-08
GO:0048661 positive regulation of smooth muscle cell proliferation	14	3.95e-08
GO:0007492 endoderm development	11	4.54e-08
GO:0001958 endochondral ossification	10	5.39e-08
GO:0048704 embryonic skeletal system morphogenesis	12	7.97e-08
GO:0030900 forebrain development	14	9.24e-08
GO:0030308 negative regulation of cell growth	16	1.34e-07
GO:0060441 epithelial tube branching involved in lung morphogenesis	9	1.73e-07
GO:0071407 cellular response to organic cyclic compound	13	1.93e-07
GO:0048589 developmental growth	10	2.22e-07
GO:0001947 heart looping	12	2.75e-07
GO:0071260 cellular response to mechanical stimulus	13	2.88e-07
GO:0071285 cellular response to lithium ion	8	2.91e-07
GO:0042475 odontogenesis of dentin-containing tooth	12	3.12e-07
GO:0030878 thyroid gland development	9	3.18e-07
GO:0002053 positive regulation of mesenchymal cell proliferation	10	3.44e-07
GO:0030335 positive regulation of cell migration	19	3.84e-07
GO:0043065 positive regulation of apoptotic process	24	4.19e-07
GO:0048662 negative regulation of smooth muscle cell proliferation	10	4.25e-07
GO:0001656 metanephros development	10	4.25e-07
GO:0032331 negative regulation of chondrocyte differentiation	8	4.27e-07
GO:0043524 negative regulation of neuron apoptotic process	17	4.53e-07
GO:0006954 inflammatory response	24	6.7e-07
GO:0030857 negative regulation of epithelial cell differentiation	7	7.69e-07
GO:0031018 endocrine pancreas development	10	8.13e-07
GO:0045668 negative regulation of osteoblast differentiation	11	1.06e-06
GO:0050680 negative regulation of epithelial cell proliferation	12	1.13e-06
GO:0007623 circadian rhythm	14	1.14e-06
GO:0001701 in utero embryonic development	22	1.15e-06
GO:0030890 positive regulation of B cell proliferation	10	1.19e-06
GO:0008543 fibroblast growth factor receptor signaling pathway	10	1.19e-06
GO:0048557 embryonic digestive tract morphogenesis	8	1.26e-06
GO:0001822 kidney development	15	1.32e-06
GO:0007389 pattern specification process	11	1.37e-06
GO:0048663 neuron fate commitment	9	1.53e-06
GO:0045736 negative regulation of cyclin-dependent protein serine/threonine kinase activity	8	1.74e-06
GO:0042060 wound healing	13	1.84e-06
GO:0031016 pancreas development	9	1.92e-06
GO:0030949 positive regulation of vascular endothelial growth factor receptor signaling pathway	7	2.01e-06
GO:0048715 negative regulation of oligodendrocyte differentiation	7	2.01e-06
GO:0035019 somatic stem cell population maintenance	10	2.02e-06

GO:0071560 cellular response to transforming growth factor beta stimulus	11	2.09e-06
GO:0048709 oligodendrocyte differentiation	9	2.36e-06
GO:0048863 stem cell differentiation	9	2.36e-06
GO:0001934 positive regulation of protein phosphorylation	17	2.56e-06
GO:0010942 positive regulation of cell death	10	2.82e-06
GO:0031069 hair follicle morphogenesis	9	2.92e-06
GO:0001759 organ induction	7	3.05e-06
GO:0060425 lung morphogenesis	8	3.06e-06
GO:0035115 embryonic forelimb morphogenesis	9	3.58e-06
GO:0050731 positive regulation of peptidyl-tyrosine phosphorylation	13	3.62e-06
GO:0030916 otic vesicle formation	6	4.94e-06
GO:0008283 cell proliferation	18	5.1e-06
GO:0009954 proximal/distal pattern formation	8	5.31e-06
GO:0001944 vasculature development	8	5.31e-06
GO:0002052 positive regulation of neuroblast proliferation	8	5.31e-06
GO:0007568 aging	16	6.19e-06
GO:0033077 T cell differentiation in thymus	9	6.55e-06
GO:0003151 outflow tract morphogenesis	10	7.3e-06
GO:0001569 patterning of blood vessels	9	7.92e-06
GO:0042472 inner ear morphogenesis	11	8.95e-06
GO:0051145 smooth muscle cell differentiation	7	9.7e-06
GO:0002063 chondrocyte development	7	9.7e-06
GO:0002076 osteoblast development	7	9.7e-06
GO:0042326 negative regulation of phosphorylation	7	9.7e-06
GO:0032526 response to retinoic acid	10	1.14e-05
GO:0030318 melanocyte differentiation	7	1.93e-05
GO:0008217 regulation of blood pressure	10	2.34e-05
GO:0001823 mesonephros development	6	2.65e-05
GO:0090090 negative regulation of canonical Wnt signaling pathway	12	2.91e-05
GO:0007267 cell-cell signaling	12	3.18e-05
GO:0001657 ureteric bud development	9	3.18e-05
GO:0048146 positive regulation of fibroblast proliferation	10	3.38e-05
GO:0007399 nervous system development	22	3.71e-05
GO:0030182 neuron differentiation	13	3.72e-05
GO:0048839 inner ear development	10	3.75e-05
GO:0048511 rhythmic process	13	3.98e-05
GO:0030855 epithelial cell differentiation	10	4.2e-05
GO:0030097 hemopoiesis	11	4.21e-05
GO:0001708 cell fate specification	7	4.3e-05
GO:0035050 embryonic heart tube development	7	4.3e-05
GO:0060045 positive regulation of cardiac muscle cell proliferation	7	4.3e-05
GO:0023019 signal transduction involved in regulation of gene expression	7	4.3e-05
GO:0030509 BMP signaling pathway	11	4.61e-05
GO:0001570 vasculogenesis	10	4.62e-05
GO:0045786 negative regulation of cell cycle	8	4.7e-05
GO:0030902 hindbrain development	7	5.47e-05
GO:0009880 embryonic pattern specification	7	5.47e-05
GO:0007417 central nervous system development	11	5.51e-05
GO:0045747 positive regulation of Notch signaling pathway	8	5.58e-05
GO:0030501 positive regulation of bone mineralization	8	5.58e-05
GO:0008584 male gonad development	12	6.42e-05
GO:0009952 anterior/posterior pattern specification	12	6.42e-05
GO:0002320 lymphoid progenitor cell differentiation	5	6.5e-05
GO:0045596 negative regulation of cell differentiation	12	7.52e-05
GO:0030198 extracellular matrix organization	12	7.52e-05
GO:0048701 embryonic cranial skeleton morphogenesis	8	7.85e-05
GO:0045595 regulation of cell differentiation	8	7.85e-05
GO:0046427 positive regulation of JAK-STAT cascade	7	8.62e-05
GO:0048568 embryonic organ development	8	9.24e-05
GO:0045471 response to ethanol	12	9.42e-05

GO:0042476 odontogenesis	7	0.000107
GO:0030217 T cell differentiation	8	0.000107
GO:0014807 regulation of somitogenesis	5	0.000121
GO:0035108 limb morphogenesis	7	0.000131
GO:2000352 negative regulation of endothelial cell apoptotic process	7	0.000131
GO:0014902 myotube differentiation	6	0.000153
GO:0050678 regulation of epithelial cell proliferation	6	0.000153
GO:0035994 response to muscle stretch	6	0.000153
GO:0030224 monocyte differentiation	6	0.000153
GO:0045930 negative regulation of mitotic cell cycle	7	0.00016
GO:0071347 cellular response to interleukin-1	10	0.00016
GO:0007050 cell cycle arrest	10	0.000175
GO:0051781 positive regulation of cell division	8	0.000193
GO:0021983 pituitary gland development	7	0.000194
GO:0021537 telencephalon development	7	0.000194
GO:0043410 positive regulation of MAPK cascade	11	0.000198
GO:0009612 response to mechanical stimulus	9	0.000204
GO:0003007 heart morphogenesis	9	0.000228
GO:0060021 palate development	10	0.00025
GO:0071300 cellular response to retinoic acid	9	0.000253
GO:0048598 embryonic morphogenesis	6	0.000258
GO:0021904 dorsal/ventral neural tube patterning	6	0.000258
GO:0048286 lung alveolus development	8	0.000287
GO:0090263 positive regulation of canonical Wnt signaling pathway	9	0.000309
GO:0001938 positive regulation of endothelial cell proliferation	9	0.000309
GO:0071356 cellular response to tumor necrosis factor	11	0.00031
GO:0071392 cellular response to estradiol stimulus	7	0.000322
GO:0001502 cartilage condensation	6	0.000329
GO:0060070 canonical Wnt signaling pathway	10	0.000346
GO:0001890 placenta development	8	0.000363
GO:0000165 MAPK cascade	9	0.000372
GO:0043433 negative regulation of sequence-specific DNA binding transcription factor activity	9	0.000372
GO:0021915 neural tube development	8	0.00041
GO:0030279 negative regulation of ossification	6	0.000412
GO:0001707 mesoderm formation	7	0.000439
GO:0071773 cellular response to BMP stimulus	7	0.000439
GO:0060484 lung-associated mesenchyme development	5	0.000462
GO:0060174 limb bud formation	5	0.000462
GO:0043401 steroid hormone mediated signaling pathway	8	0.000518
GO:0043406 positive regulation of MAP kinase activity	8	0.000583
GO:0033138 positive regulation of peptidyl-serine phosphorylation	9	0.000595
GO:0055007 cardiac muscle cell differentiation	7	0.000596
GO:0071549 cellular response to dexamethasone stimulus	7	0.000596
GO:0090190 positive regulation of branching involved in ureteric bud morphogenesis	6	0.000635
GO:0035690 cellular response to drug	9	0.000649
GO:0007179 transforming growth factor beta receptor signaling pathway	9	0.000649
GO:0043627 response to estrogen	9	0.000649
GO:0051384 response to glucocorticoid	9	0.000649
GO:0021879 forebrain neuron differentiation	5	0.000655
GO:0030879 mammary gland development	7	0.000677
GO:0045665 negative regulation of neuron differentiation	9	0.000702
GO:0045216 cell-cell junction organization	6	0.000764
GO:0003148 outflow tract septum morphogenesis	6	0.000764
GO:0060395 SMAD protein signal transduction	9	0.000768
GO:0007154 cell communication	7	0.000771
GO:0043010 camera-type eye development	9	0.000829
GO:0009749 response to glucose	9	0.000829
GO:0007160 cell-matrix adhesion	9	0.000829

GO:0001714 endodermal cell fate specification	4	0.000845
GO:0051150 regulation of smooth muscle cell differentiation	4	0.000845
GO:0002326 B cell lineage commitment	4	0.000845
GO:0060512 prostate gland morphogenesis	4	0.000845
GO:0009611 response to wounding	8	0.000866
GO:0003198 epithelial to mesenchymal transition involved in endocardial cushion formation	5	0.000886
GO:0048599 oocyte development	5	0.000886
GO:0008406 gonad development	5	0.000886
GO:0006955 immune response	16	0.000905
GO:0071456 cellular response to hypoxia	10	0.000968
GO:0010595 positive regulation of endothelial cell migration	7	0.000993
GO:0045662 negative regulation of myoblast differentiation	6	0.00109
GO:0048565 digestive tract development	7	0.00113
GO:0007420 brain development	14	0.00116
GO:0030336 negative regulation of cell migration	10	0.00117
GO:0060979 vasculogenesis involved in coronary vascular morphogenesis	5	0.00118
GO:0016337 single organismal cell-cell adhesion	10	0.00135
GO:0048706 embryonic skeletal system development	7	0.00144
GO:0071363 cellular response to growth factor stimulus	8	0.00154
GO:0050918 positive chemotaxis	5	0.00154
GO:0060445 branching involved in salivary gland morphogenesis	5	0.00154
GO:0051726 regulation of cell cycle	10	0.00175
GO:0045840 positive regulation of mitotic nuclear division	6	0.0018
GO:0031214 biomineral tissue development	6	0.0018
GO:0055008 cardiac muscle tissue morphogenesis	5	0.002
GO:0033280 response to vitamin D	5	0.002
GO:0050796 regulation of insulin secretion	7	0.00204
GO:0006006 glucose metabolic process	8	0.0022
GO:0045599 negative regulation of fat cell differentiation	7	0.00227
GO:0043525 positive regulation of neuron apoptotic process	8	0.00239
GO:0001837 epithelial to mesenchymal transition	6	0.00242
GO:0002042 cell migration involved in sprouting angiogenesis	5	0.0025
GO:0042789 mRNA transcription from RNA polymerase II promoter	5	0.0025
GO:0009953 dorsal/ventral pattern formation	7	0.0025
GO:0032808 lacrimal gland development	4	0.00252
GO:2000144 positive regulation of DNA-templated transcription, initiation	4	0.00252
GO:0003180 aortic valve morphogenesis	4	0.00252
GO:0060664 epithelial cell proliferation involved in salivary gland morphogenesis	4	0.00252
GO:0002674 negative regulation of acute inflammatory response	4	0.00252
GO:0060687 regulation of branching involved in prostate gland morphogenesis	4	0.00252
GO:0060349 bone morphogenesis	6	0.00278
GO:0050776 regulation of immune response	6	0.00278
GO:0001568 blood vessel development	8	0.00278
GO:0007224 smoothened signaling pathway	8	0.00278
GO:0071333 cellular response to glucose stimulus	8	0.00278
GO:0051591 response to cAMP	7	0.00307
GO:0060037 pharyngeal system development	5	0.00308
GO:0048566 embryonic digestive tract development	5	0.00308
GO:0043523 regulation of neuron apoptotic process	6	0.00316
GO:0043434 response to peptide hormone	8	0.00324
GO:0001764 neuron migration	10	0.0035
GO:0010718 positive regulation of epithelial to mesenchymal transition	6	0.00361
GO:0050873 brown fat cell differentiation	6	0.00361
GO:0060428 lung epithelium development	4	0.00381
GO:0060603 mammary gland duct morphogenesis	4	0.00381
GO:0030282 bone mineralization	6	0.00411
GO:0034504 protein localization to nucleus	6	0.00411
GO:0035914 skeletal muscle cell differentiation	7	0.0045
GO:0030539 male genitalia development	5	0.00456

GO:0009653 anatomical structure morphogenesis	5	0.00456
GO:0090398 cellular senescence	5	0.00456
GO:0030513 positive regulation of BMP signaling pathway	6	0.00464
GO:0040007 growth	6	0.00464
GO:1901215 negative regulation of neuron death	7	0.0049
GO:0030177 positive regulation of Wnt signaling pathway	6	0.00525
GO:0016477 cell migration	12	0.00526
GO:0001541 ovarian follicle development	7	0.00532
GO:0008354 germ cell migration	4	0.00539
GO:0060290 transdifferentiation	4	0.00539
GO:0061045 negative regulation of wound healing	4	0.00539
GO:0030500 regulation of bone mineralization	5	0.00541
GO:0051894 positive regulation of focal adhesion assembly	5	0.00541
GO:0007519 skeletal muscle tissue development	7	0.00577
GO:0048666 neuron development	7	0.00577
GO:0042246 tissue regeneration	5	0.00643
GO:0042981 regulation of apoptotic process	12	0.00728
GO:0045787 positive regulation of cell cycle	6	0.00733
GO:0045839 negative regulation of mitotic nuclear division	4	0.00742
GO:0007184 SMAD protein import into nucleus	4	0.00742
GO:2000727 positive regulation of cardiac muscle cell differentiation	4	0.00742
GO:0090399 replicative senescence	4	0.00742
GO:0032922 circadian regulation of gene expression	7	0.00742
GO:0000082 G1/S transition of mitotic cell cycle	7	0.00807
GO:0050728 negative regulation of inflammatory response	8	0.00866
GO:0030574 collagen catabolic process	5	0.00882
GO:0042102 positive regulation of T cell proliferation	7	0.00944
GO:0045603 positive regulation of endothelial cell differentiation	4	0.00981
GO:0048103 somatic stem cell division	4	0.00981
GO:0048853 forebrain morphogenesis	4	0.00981
GO:0032495 response to muramyl dipeptide	4	0.00981
GO:0045740 positive regulation of DNA replication	6	0.00993
GO:0018108 peptidyl-tyrosine phosphorylation	7	0.0101
GO:0010033 response to organic substance	7	0.0101
GO:0022008 neurogenesis	7	0.0101
GO:0048593 camera-type eye morphogenesis	5	0.0101
GO:0045672 positive regulation of osteoclast differentiation	5	0.0101
GO:0071222 cellular response to lipopolysaccharide	12	0.0104
GO:0006919 activation of cysteine-type endopeptidase activity involved in apoptotic process	7	0.0116
GO:0046639 negative regulation of alpha-beta T cell differentiation	3	0.0117
GO:0060594 mammary gland specification	3	0.0117
GO:0060523 prostate epithelial cord elongation	3	0.0117
GO:0060916 mesenchymal cell proliferation involved in lung development	3	0.0117
GO:0061047 positive regulation of branching involved in lung morphogenesis	3	0.0117
GO:0072125 negative regulation of glomerular mesangial cell proliferation	3	0.0117
GO:0032967 positive regulation of collagen biosynthetic process	5	0.0117
GO:0007411 axon guidance	10	0.0117
GO:0045429 positive regulation of nitric oxide biosynthetic process	6	0.0118
GO:0035094 response to nicotine	6	0.0118
GO:0042640 anagen	4	0.0124
GO:0033089 positive regulation of T cell differentiation in thymus	4	0.0124
GO:0060272 embryonic skeletal joint morphogenesis	4	0.0124
GO:0014003 oligodendrocyte development	5	0.0132
GO:0030325 adrenal gland development	5	0.0132
GO:0035987 endodermal cell differentiation	5	0.0132
GO:0043966 histone H3 acetylation	6	0.0143
GO:0055010 ventricular cardiac muscle tissue morphogenesis	5	0.0151
GO:0051897 positive regulation of protein kinase B signaling	8	0.0153
GO:0019827 stem cell population maintenance	7	0.0153

GO:0010832 negative regulation of myotube differentiation	4	0.0155
GO:0043583 ear development	4	0.0155
GO:0048484 enteric nervous system development	4	0.0155
GO:0007155 cell adhesion	19	0.0158
GO:0045600 positive regulation of fat cell differentiation	6	0.0168
GO:0001755 neural crest cell migration	6	0.0168
GO:0043586 tongue development	4	0.0191
GO:2000811 negative regulation of anoikis	4	0.0191
GO:0030225 macrophage differentiation	4	0.0191
GO:2000279 negative regulation of DNA biosynthetic process	4	0.0191
GO:0042149 cellular response to glucose starvation	5	0.0192
GO:0042517 positive regulation of tyrosine phosphorylation of Stat3 protein	5	0.0192
GO:0032570 response to progesterone	5	0.0192
GO:0071479 cellular response to ionizing radiation	5	0.0214
GO:0050767 regulation of neurogenesis	5	0.0214
GO:0031663 lipopolysaccharide-mediated signaling pathway	5	0.0214
GO:0043552 positive regulation of phosphatidylinositol 3-kinase activity	5	0.0214
GO:0003257 positive regulation of transcription from RNA polymerase II promoter involved in myocardial precursor cell differentiation	3	0.0215
GO:0003338 metanephros morphogenesis	3	0.0215
GO:0033092 positive regulation of immature T cell proliferation in thymus	3	0.0215
GO:0021892 cerebral cortex GABAergic interneuron differentiation	3	0.0215
GO:0060129 thyroid-stimulating hormone-secreting cell differentiation	3	0.0215
GO:0002677 negative regulation of chronic inflammatory response	3	0.0215
GO:0007442 hindgut morphogenesis	3	0.0215
GO:0060688 regulation of morphogenesis of a branching structure	3	0.0215
GO:0060364 frontal suture morphogenesis	3	0.0215
GO:0006959 humoral immune response	6	0.0216
GO:0050673 epithelial cell proliferation	4	0.0232
GO:0045019 negative regulation of nitric oxide biosynthetic process	4	0.0232
GO:0060347 heart trabecula formation	4	0.0232
GO:0008593 regulation of Notch signaling pathway	4	0.0232
GO:0000060 protein import into nucleus, translocation	5	0.0239
GO:0043388 positive regulation of DNA binding	5	0.0239
GO:0048705 skeletal system morphogenesis	6	0.0252
GO:0030512 negative regulation of transforming growth factor beta receptor signaling pathway	6	0.0252
GO:0019216 regulation of lipid metabolic process	5	0.0266
GO:0071345 cellular response to cytokine stimulus	5	0.0266
GO:0046326 positive regulation of glucose import	5	0.0266
GO:2001240 negative regulation of extrinsic apoptotic signaling pathway in absence of ligand	5	0.0266
GO:0006366 transcription from RNA polymerase II promoter	9	0.0267
GO:0014070 response to organic cyclic compound	7	0.0268
GO:0060548 negative regulation of cell death	7	0.0268
GO:0048538 thymus development	6	0.0269
GO:0035265 organ growth	4	0.0274
GO:0032270 positive regulation of cellular protein metabolic process	4	0.0274
GO:0061036 positive regulation of cartilage development	4	0.0274
GO:0050995 negative regulation of lipid catabolic process	4	0.0274
GO:0050872 white fat cell differentiation	4	0.0274
GO:0007368 determination of left/right symmetry	7	0.0281
GO:0007565 female pregnancy	7	0.0281
GO:0030178 negative regulation of Wnt signaling pathway	6	0.0288
GO:0031100 organ regeneration	6	0.0288
GO:0060612 adipose tissue development	5	0.0289
GO:0050900 leukocyte migration	5	0.0289
GO:0042177 negative regulation of protein catabolic process	5	0.0321
GO:0060038 cardiac muscle cell proliferation	4	0.0321
GO:0003203 endocardial cushion morphogenesis	4	0.0321

GO:0035137 hindlimb morphogenesis	4	0.0321
GO:0003158 endothelium development	3	0.0331
GO:0060513 prostatic bud formation	3	0.0331
GO:0061419 positive regulation of transcription from RNA polymerase II promoter in response to hypoxia	3	0.0331
GO:0002573 myeloid leukocyte differentiation	3	0.0331
GO:0033088 negative regulation of immature T cell proliferation in thymus	3	0.0331
GO:0033085 negative regulation of T cell differentiation in thymus	3	0.0331
GO:0003211 cardiac ventricle formation	3	0.0331
GO:0021544 subpallium development	3	0.0331
GO:0002520 immune system development	3	0.0331
GO:2000987 positive regulation of behavioral fear response	3	0.0331
GO:0060033 anatomical structure regression	3	0.0331
GO:0002931 response to ischemia	5	0.0352
GO:0010634 positive regulation of epithelial cell migration	5	0.0352
GO:0043200 response to amino acid	5	0.0352
GO:0007369 gastrulation	5	0.0352
GO:0043408 regulation of MAPK cascade	6	0.0354
GO:0006915 apoptotic process	20	0.0359
GO:0048856 anatomical structure development	4	0.0373
GO:0060173 limb development	5	0.0383
GO:0050727 regulation of inflammatory response	6	0.0402
GO:0032869 cellular response to insulin stimulus	7	0.0405
GO:0043491 protein kinase B signaling	5	0.0417
GO:0045909 positive regulation of vasodilation	5	0.0417
GO:0048255 mRNA stabilization	4	0.0428
GO:0060749 mammary gland alveolus development	4	0.0428
GO:0060065 uterus development	4	0.0428
GO:2000045 regulation of G1/S transition of mitotic cell cycle	4	0.0428
GO:0042307 positive regulation of protein import into nucleus	4	0.0428
GO:0035264 multicellular organism growth	8	0.0449
GO:0043124 negative regulation of I-kappaB kinase/NF-kappaB signaling	5	0.0453
GO:0030199 collagen fibril organization	5	0.0453
GO:0060669 embryonic placenta morphogenesis	3	0.0465
GO:0002360 T cell lineage commitment	3	0.0465
GO:1902262 apoptotic process involved in patterning of blood vessels	3	0.0465
GO:0006029 proteoglycan metabolic process	3	0.0465
GO:2001054 negative regulation of mesenchymal cell apoptotic process	3	0.0465
GO:0001936 regulation of endothelial cell proliferation	3	0.0465
GO:0045766 positive regulation of angiogenesis	8	0.0467
GO:0044130 negative regulation of growth of symbiont in host	4	0.0488
GO:0048066 developmental pigmentation	4	0.0488
GO:0043403 skeletal muscle tissue regeneration	4	0.0488
GO:0001654 eye development	5	0.0489
GO:0060325 face morphogenesis	5	0.0489

Table S15: Enriched GO terms (top) with adjusted p-values < 0.05 for the identified targets in the HE stage.

Enriched terms	count	adj. p-values
GO:0045893 positive regulation of transcription, DNA-templated	52	1.32e-27
GO:0045944 positive regulation of transcription from RNA polymerase II promoter	61	2.04e-24
GO:0000122 negative regulation of transcription from RNA polymerase II promoter	52	2.25e-23
GO:0010628 positive regulation of gene expression	41	2.25e-23
GO:0001525 angiogenesis	28	1.39e-16
GO:0010629 negative regulation of gene expression	28	1.69e-15
GO:0008285 negative regulation of cell proliferation	32	3.39e-15
GO:0007275 multicellular organism development	48	3.73e-14
GO:0007507 heart development	25	1.03e-12

GO:0006355 regulation of transcription, DNA-templated	70	1.67e-12
GO:0006351 transcription, DNA-templated	62	4.98e-12
GO:0008284 positive regulation of cell proliferation	33	5.32e-12
GO:0006357 regulation of transcription from RNA polymerase II promoter	27	1.46e-10
GO:0045892 negative regulation of transcription, DNA-templated	32	1.49e-10
GO:0007179 transforming growth factor beta receptor signaling pathway	14	4.84e-10
GO:0045766 positive regulation of angiogenesis	16	1.43e-09
GO:0003151 outflow tract morphogenesis	12	3.34e-09
GO:0030217 T cell differentiation	11	3.82e-09
GO:0043627 response to estrogen	13	7.23e-09
GO:0007155 cell adhesion	27	8.04e-09
GO:0010468 regulation of gene expression	22	8.37e-09
GO:0001934 positive regulation of protein phosphorylation	17	4.43e-08
GO:0042493 response to drug	22	4.46e-08
GO:0045165 cell fate commitment	12	6.24e-08
GO:0001666 response to hypoxia	17	7.65e-08
GO:0030154 cell differentiation	32	1.44e-07
GO:0048661 positive regulation of smooth muscle cell proliferation	12	1.71e-07
GO:0043066 negative regulation of apoptotic process	27	1.72e-07
GO:0043065 positive regulation of apoptotic process	21	1.72e-07
GO:0030900 forebrain development	12	3.51e-07
GO:0030509 BMP signaling pathway	12	3.85e-07
GO:0071773 cellular response to BMP stimulus	9	3.91e-07
GO:0045786 negative regulation of cell cycle	9	4.83e-07
GO:0003148 outflow tract septum morphogenesis	8	5.4e-07
GO:0001568 blood vessel development	11	6.01e-07
GO:0001701 in utero embryonic development	19	8.19e-07
GO:0035914 skeletal muscle cell differentiation	10	8.32e-07
GO:0071560 cellular response to transforming growth factor beta stimulus	10	2.08e-06
GO:0070374 positive regulation of ERK1 and ERK2 cascade	15	2.47e-06
GO:0051091 positive regulation of sequence-specific DNA binding transcription factor activity	12	2.64e-06
GO:0042475 odontogenesis of dentin-containing tooth	10	2.65e-06
GO:0016477 cell migration	15	2.8e-06
GO:0002062 chondrocyte differentiation	9	2.9e-06
GO:0042127 regulation of cell proliferation	16	3.47e-06
GO:0045669 positive regulation of osteoblast differentiation	10	4.66e-06
GO:0001570 vasculogenesis	10	4.66e-06
GO:0030335 positive regulation of cell migration	15	5.48e-06
GO:1902895 positive regulation of pri-miRNA transcription from RNA polymerase II promoter	7	7.67e-06
GO:0001503 ossification	11	8.35e-06
GO:0030324 lung development	12	1.02e-05
GO:0007219 Notch signaling pathway	12	1.08e-05
GO:0071456 cellular response to hypoxia	11	1.53e-05
GO:0042523 positive regulation of tyrosine phosphorylation of Stat5 protein	6	1.78e-05
GO:0051216 cartilage development	10	2.05e-05
GO:0008217 regulation of blood pressure	9	2.55e-05
GO:0030890 positive regulation of B cell proliferation	8	2.59e-05
GO:0009612 response to mechanical stimulus	9	2.79e-05
GO:0030097 hemopoiesis	10	2.87e-05
GO:0048469 cell maturation	8	3.39e-05
GO:0071542 dopaminergic neuron differentiation	7	3.64e-05
GO:0030855 epithelial cell differentiation	9	4.22e-05
GO:0001938 positive regulation of endothelial cell proliferation	9	4.22e-05
GO:0001958 endochondral ossification	7	4.36e-05
GO:0030224 monocyte differentiation	6	4.5e-05
GO:0007411 axon guidance	12	5.04e-05
GO:0051145 smooth muscle cell differentiation	6	5.98e-05
GO:0032355 response to estradiol	10	9.72e-05

GO:0050731 positive regulation of peptidyl-tyrosine phosphorylation	10	9.72e-05
GO:0043524 negative regulation of neuron apoptotic process	12	9.83e-05
GO:0048514 blood vessel morphogenesis	7	0.000103
GO:0045666 positive regulation of neuron differentiation	10	0.000111
GO:0006955 immune response	15	0.000124
GO:0055007 cardiac muscle cell differentiation	7	0.000139
GO:0001501 skeletal system development	10	0.000146
GO:0006366 transcription from RNA polymerase II promoter	11	0.000157
GO:0048662 negative regulation of smooth muscle cell proliferation	7	0.000157
GO:0002687 positive regulation of leukocyte migration	6	0.000157
GO:0043011 myeloid dendritic cell differentiation	6	0.000157
GO:0009948 anterior/posterior axis specification	6	0.000157
GO:0007568 aging	12	0.000182
GO:0009952 anterior/posterior pattern specification	10	0.000199
GO:0001569 patterning of blood vessels	7	0.000203
GO:0001947 heart looping	8	0.000229
GO:0030574 collagen catabolic process	6	0.00023
GO:0042104 positive regulation of activated T cell proliferation	6	0.00023
GO:0048646 anatomical structure formation involved in morphogenesis	6	0.00023
GO:0001974 blood vessel remodeling	7	0.000301
GO:0001944 vasculature development	6	0.000336
GO:0006954 inflammatory response	16	0.000346
GO:0060411 cardiac septum morphogenesis	5	0.000357
GO:0035987 endodermal cell differentiation	6	0.000394
GO:0001764 neuron migration	10	0.000409
GO:0045597 positive regulation of cell differentiation	7	0.000419
GO:0043433 negative regulation of sequence-specific DNA binding transcription factor activity	8	0.000426
GO:2000352 negative regulation of endothelial cell apoptotic process	6	0.000454
GO:0030182 neuron differentiation	10	0.000474
GO:0001657 ureteric bud development	7	0.000514
GO:0043392 negative regulation of DNA binding	6	0.000526
GO:0050680 negative regulation of epithelial cell proliferation	8	0.000534
GO:0048468 cell development	7	0.000562
GO:0007267 cell-cell signaling	9	0.000684
GO:0035690 cellular response to drug	8	0.000684
GO:0045746 negative regulation of Notch signaling pathway	6	7e-04
GO:0050679 positive regulation of epithelial cell proliferation	8	0.000723
GO:0045665 negative regulation of neuron differentiation	8	0.000723
GO:0033280 response to vitamin D	5	0.000748
GO:0060395 SMAD protein signal transduction	8	0.000772
GO:0071260 cellular response to mechanical stimulus	8	0.000823
GO:0043401 steroid hormone mediated signaling pathway	7	0.000825
GO:0048596 embryonic camera-type eye morphogenesis	5	0.000931
GO:0009887 organ morphogenesis	9	0.00095
GO:0010718 positive regulation of epithelial to mesenchymal transition	6	0.00103
GO:0051726 regulation of cell cycle	9	0.00113
GO:0042326 negative regulation of phosphorylation	5	0.00114
GO:0014898 cardiac muscle hypertrophy in response to stress	5	0.00114
GO:0001649 osteoblast differentiation	9	0.00118
GO:0030198 extracellular matrix organization	9	0.00125
GO:0048754 branching morphogenesis of an epithelial tube	6	0.0013
GO:0060021 palate development	8	0.00132
GO:0002053 positive regulation of mesenchymal cell proliferation	6	0.00145
GO:0045471 response to ethanol	9	0.00146
GO:0050728 negative regulation of inflammatory response	8	0.00149
GO:0060412 ventricular septum morphogenesis	6	0.00163
GO:0007417 central nervous system development	8	0.00169
GO:0060070 canonical Wnt signaling pathway	8	0.00169
GO:0048701 embryonic cranial skeleton morphogenesis	6	0.00182

GO:0061314 Notch signaling involved in heart development	4	0.00182
GO:0060485 mesenchyme development	4	0.00182
GO:0021978 telencephalon regionalization	4	0.00182
GO:0045348 positive regulation of MHC class II biosynthetic process	4	0.00182
GO:0045591 positive regulation of regulatory T cell differentiation	4	0.00182
GO:0045787 positive regulation of cell cycle	6	0.00203
GO:0003007 heart morphogenesis	7	0.00204
GO:0042102 positive regulation of T cell proliferation	7	0.00204
GO:0030308 negative regulation of cell growth	9	0.00216
GO:0048146 positive regulation of fibroblast proliferation	7	0.00219
GO:0001889 liver development	8	0.00242
GO:0007399 nervous system development	15	0.00256
GO:0003208 cardiac ventricle morphogenesis	4	0.00258
GO:0060045 positive regulation of cardiac muscle cell proliferation	5	0.00268
GO:0023019 signal transduction involved in regulation of gene expression	5	0.00268
GO:0060425 lung morphogenesis	5	0.00316
GO:0071158 positive regulation of cell cycle arrest	5	0.00316
GO:0071222 cellular response to lipopolysaccharide	11	0.00319
GO:0007169 transmembrane receptor protein tyrosine kinase signaling pathway	8	0.0032
GO:0060710 chorio-allantoic fusion	4	0.00348
GO:0048304 positive regulation of isotype switching to IgG isotypes	4	0.00348
GO:2000727 positive regulation of cardiac muscle cell differentiation	4	0.00348
GO:0021772 olfactory bulb development	5	0.00361
GO:0045672 positive regulation of osteoclast differentiation	5	0.00361
GO:0010667 negative regulation of cardiac muscle cell apoptotic process	5	0.00419
GO:0045599 negative regulation of fat cell differentiation	6	0.00437
GO:0001658 branching involved in ureteric bud morphogenesis	6	0.00437
GO:0008283 cell proliferation	11	0.00459
GO:0003184 pulmonary valve morphogenesis	4	0.0046
GO:0008347 glial cell migration	4	0.0046
GO:0032689 negative regulation of interferon-gamma production	5	0.0047
GO:0009953 dorsal/ventral pattern formation	6	0.00472
GO:0001890 placenta development	6	0.00512
GO:0007160 cell-matrix adhesion	7	0.00527
GO:0032700 negative regulation of interleukin-17 production	4	0.00582
GO:0032740 positive regulation of interleukin-17 production	4	0.00582
GO:0060548 negative regulation of cell death	7	0.00596
GO:0048738 cardiac muscle tissue development	5	0.00602
GO:0097421 liver regeneration	5	0.00602
GO:0030316 osteoclast differentiation	5	0.00602
GO:0034097 response to cytokine	7	0.00629
GO:0007050 cell cycle arrest	7	0.00629
GO:0044344 cellular response to fibroblast growth factor stimulus	5	0.00679
GO:0048535 lymph node development	5	0.00679
GO:0003197 endocardial cushion development	4	0.00718
GO:0003215 cardiac right ventricle morphogenesis	4	0.00718
GO:0060389 pathway-restricted SMAD protein phosphorylation	4	0.00718
GO:2000111 positive regulation of macrophage apoptotic process	3	0.00723
GO:1901215 negative regulation of neuron death	6	0.00817
GO:0031100 organ regeneration	6	0.00817
GO:0050829 defense response to Gram-negative bacterium	6	0.00817
GO:0032496 response to lipopolysaccharide	10	0.00829
GO:0048663 neuron fate commitment	5	0.00845
GO:0043388 positive regulation of DNA binding	5	0.00845
GO:0001541 ovarian follicle development	6	0.0087
GO:0001775 cell activation	4	0.0087
GO:0030949 positive regulation of vascular endothelial growth factor receptor signaling pathway	4	0.0087
GO:0002376 immune system process	14	0.00873
GO:2001240 negative regulation of extrinsic apoptotic signaling pathway in absence of ligand	5	0.00921

GO:0009611 response to wounding	6	0.00924
GO:0007389 pattern specification process	6	0.00924
GO:0032760 positive regulation of tumor necrosis factor production	6	0.00987
GO:0010332 response to gamma radiation	5	0.0102
GO:0071498 cellular response to fluid shear stress	4	0.0104
GO:0003181 atrioventricular valve morphogenesis	4	0.0104
GO:0007566 embryo implantation	6	0.0105
GO:0048511 rhythmic process	8	0.0116
GO:2000679 positive regulation of transcription regulatory region DNA binding	4	0.0123
GO:0042060 wound healing	7	0.0123
GO:0030513 positive regulation of BMP signaling pathway	5	0.0124
GO:0040007 growth	5	0.0124
GO:0071288 cellular response to mercury ion	3	0.0126
GO:0003199 endocardial cushion to mesenchymal transition involved in heart valve formation	3	0.0126
GO:0030326 embryonic limb morphogenesis	6	0.0127
GO:0030501 positive regulation of bone mineralization	5	0.0134
GO:0071363 cellular response to growth factor stimulus	6	0.0134
GO:0006979 response to oxidative stress	8	0.0138
GO:0071300 cellular response to retinoic acid	6	0.0142
GO:0003222 ventricular trabecula myocardium morphogenesis	4	0.0142
GO:1900745 positive regulation of p38MAPK cascade	4	0.0142
GO:0003281 ventricular septum development	5	0.0144
GO:0006468 protein phosphorylation	17	0.0157
GO:0014911 positive regulation of smooth muscle cell migration	5	0.0157
GO:0060037 pharyngeal system development	4	0.0165
GO:0021542 dentate gyrus development	4	0.0165
GO:0001774 microglial cell activation	4	0.0165
GO:0007492 endoderm development	5	0.017
GO:0090090 negative regulation of canonical Wnt signaling pathway	7	0.0174
GO:0010595 positive regulation of endothelial cell migration	5	0.0185
GO:0043410 positive regulation of MAPK cascade	7	0.0188
GO:0046330 positive regulation of JNK cascade	6	0.0188
GO:0050880 regulation of blood vessel size	4	0.0189
GO:0021904 dorsal/ventral neural tube patterning	4	0.0189
GO:0051150 regulation of smooth muscle cell differentiation	3	0.019
GO:0060948 cardiac vascular smooth muscle cell development	3	0.019
GO:0051891 positive regulation of cardioblast differentiation	3	0.019
GO:0042482 positive regulation of odontogenesis	3	0.019
GO:0048565 digestive tract development	5	0.0197
GO:0042472 inner ear morphogenesis	6	0.0207
GO:0045740 positive regulation of DNA replication	5	0.0212
GO:0022409 positive regulation of cell-cell adhesion	4	0.0212
GO:0045780 positive regulation of bone resorption	4	0.0212
GO:0016337 single organismal cell-cell adhesion	7	0.022
GO:0007623 circadian rhythm	7	0.022
GO:0033138 positive regulation of peptidyl-serine phosphorylation	6	0.0229
GO:0071356 cellular response to tumor necrosis factor	7	0.0239
GO:0030279 negative regulation of ossification	4	0.024
GO:0035162 embryonic hemopoiesis	4	0.024
GO:0010460 positive regulation of heart rate	4	0.024
GO:0010165 response to X-ray	4	0.024
GO:0000187 activation of MAPK activity	6	0.0253
GO:0030334 regulation of cell migration	6	0.0265
GO:0055012 ventricular cardiac muscle cell differentiation	3	0.0267
GO:0044336 canonical Wnt signaling pathway involved in negative regulation of apoptotic process	3	0.0267
GO:0036006 cellular response to macrophage colony-stimulating factor stimulus	3	0.0267
GO:0050672 negative regulation of lymphocyte proliferation	3	0.0267
GO:0007182 common-partner SMAD protein phosphorylation	3	0.0267

GO:0045582 positive regulation of T cell differentiation	4	0.027
GO:0009749 response to glucose	6	0.0278
GO:0042981 regulation of apoptotic process	9	0.0291
GO:0010942 positive regulation of cell death	5	0.0299
GO:0043280 positive regulation of cysteine-type endopeptidase activity involved in apoptotic process	5	0.0299
GO:0010862 positive regulation of pathway-restricted SMAD protein phosphorylation	5	0.0299
GO:0032733 positive regulation of interleukin-10 production	4	0.0301
GO:0001708 cell fate specification	4	0.0301
GO:0035050 embryonic heart tube development	4	0.0301
GO:0071347 cellular response to interleukin-1	6	0.0304
GO:0007565 female pregnancy	6	0.0318
GO:0014823 response to activity	5	0.0318
GO:0001755 neural crest cell migration	5	0.0318
GO:0006915 apoptotic process	16	0.0319
GO:0060441 epithelial tube branching involved in lung morphogenesis	4	0.0332
GO:0042474 middle ear morphogenesis	4	0.0332
GO:0046697 decidualization	4	0.0332
GO:0045216 cell-cell junction organization	4	0.0332
GO:0060575 intestinal epithelial cell differentiation	3	0.0347
GO:0034616 response to laminar fluid shear stress	3	0.0347
GO:1901166 neural crest cell migration involved in autonomic nervous system development	3	0.0347
GO:2000144 positive regulation of DNA-templated transcription, initiation	3	0.0347
GO:0072205 metanephric collecting duct development	3	0.0347
GO:0060440 trachea formation	3	0.0347
GO:0002043 blood vessel endothelial cell proliferation involved in sprouting angiogenesis	3	0.0347
GO:0060041 retina development in camera-type eye	6	0.0364
GO:0009615 response to virus	6	0.0364
GO:0048844 artery morphogenesis	4	0.0368
GO:0032729 positive regulation of interferon-gamma production	5	0.0381
GO:0032967 positive regulation of collagen biosynthetic process	4	0.0406
GO:0002052 positive regulation of neuroblast proliferation	4	0.0406
GO:0007498 mesoderm development	4	0.0406
GO:0071277 cellular response to calcium ion	5	0.0406
GO:0016055 Wnt signaling pathway	9	0.041
GO:0043406 positive regulation of MAP kinase activity	5	0.0428
GO:0030512 negative regulation of transforming growth factor beta receptor signaling pathway	5	0.0428
GO:0035912 dorsal aorta morphogenesis	3	0.044
GO:0042487 regulation of odontogenesis of dentin-containing tooth	3	0.044
GO:0035239 tube morphogenesis	3	0.044
GO:0014909 smooth muscle cell migration	3	0.044
GO:0048704 embryonic skeletal system morphogenesis	5	0.0452
GO:0048538 thymus development	5	0.0452
GO:0050853 B cell receptor signaling pathway	5	0.0452
GO:0001822 kidney development	7	0.0478
GO:0030178 negative regulation of Wnt signaling pathway	5	0.048
GO:0090023 positive regulation of neutrophil chemotaxis	4	0.0484
GO:0045765 regulation of angiogenesis	4	0.0484

Table S16. Enriched GO terms (top) with adjusted p-values < 0.05 for the identified targets in the HP stage.

Enriched terms	count	adj. p-values
GO:0045944 positive regulation of transcription from RNA polymerase II promoter	33	9.833186004837043E-16
GO:0045944 positive regulation of transcription from RNA polymerase II promoter	33	9.83e-16
GO:0045893 positive regulation of transcription, DNA-templated	23	1.55e-11
GO:0010628 positive regulation of gene expression	15	1.22e-06
GO:0006357 regulation of transcription from RNA polymerase II promoter	15	1.34e-06
GO:0006351 transcription, DNA-templated	30	1.49e-06
GO:0008285 negative regulation of cell proliferation	15	1.75e-06
GO:0008284 positive regulation of cell proliferation	17	1.83e-06
GO:0010468 regulation of gene expression	13	4.61e-06
GO:0042127 regulation of cell proliferation	11	2.1e-05
GO:0000122 negative regulation of transcription from RNA polymerase II promoter	17	4.42e-05
GO:0006355 regulation of transcription, DNA-templated	30	4.54e-05
GO:0048538 thymus development	6	0.000802
GO:0050731 positive regulation of peptidyl-tyrosine phosphorylation	7	0.00102
GO:0009887 organ morphogenesis	7	0.00146
GO:0050680 negative regulation of epithelial cell proliferation	6	0.00242
GO:0010629 negative regulation of gene expression	9	0.00364
GO:0034097 response to cytokine	6	0.00378
GO:0050728 negative regulation of inflammatory response	6	0.00503
GO:0045597 positive regulation of cell differentiation	5	0.00541
GO:0043066 negative regulation of apoptotic process	12	0.00576
GO:0007275 multicellular organism development	16	0.00622
GO:0045892 negative regulation of transcription, DNA-templated	12	0.00641
GO:0001701 in utero embryonic development	9	0.00647
GO:0071456 cellular response to hypoxia	6	0.00807
GO:0001829 trophectodermal cell differentiation	4	0.00831
GO:0008584 male gonad development	6	0.0115
GO:0007507 heart development	8	0.0134
GO:0001934 positive regulation of protein phosphorylation	7	0.0139
GO:0006461 protein complex assembly	5	0.0145
GO:0006955 immune response	8	0.0161
GO:0090102 cochlea development	4	0.0171
GO:0021983 pituitary gland development	4	0.0178
GO:0045165 cell fate commitment	5	0.0183
GO:0032808 lacrimal gland development	3	0.0208
GO:0071345 cellular response to cytokine stimulus	4	0.0223
GO:2001240 negative regulation of extrinsic apoptotic signaling pathway in absence of ligand	4	0.0223
GO:0048589 developmental growth	4	0.0258
GO:0008283 cell proliferation	7	0.0261
GO:0033077 T cell differentiation in thymus	4	0.0335
GO:0007435 salivary gland morphogenesis	3	0.042
GO:0051897 positive regulation of protein kinase B signaling	5	0.0424
GO:0042493 response to drug	8	0.0426
GO:0001503 ossification	5	0.0429
GO:0032720 negative regulation of tumor necrosis factor production	4	0.0432
GO:0048536 spleen development	4	0.0466
GO:0035019 somatic stem cell population maintenance	4	0.0466

Table S17. Enriched GO terms (top) with adjusted p-values < 0.05 for the identified targets in the MAC stage.

Enriched terms	count	adj. p-values
GO:0045944 positive regulation of transcription from RNA polymerase II promoter	17	1.32e-07
GO:0045893 positive regulation of transcription, DNA-templated	12	1.04e-05
GO:0006351 transcription, DNA-templated	19	1.41e-05
GO:0006355 regulation of transcription, DNA-templated	17	0.00332
GO:0010628 positive regulation of gene expression	8	0.00427
GO:0045892 negative regulation of transcription, DNA-templated	9	0.00495
GO:0007623 circadian rhythm	5	0.011
GO:0006366 transcription from RNA polymerase II promoter	5	0.0245
GO:0070932 histone H3 deacetylation	3	0.0317

Table S18: List of 243 correlated genes that have Spearman correlation above 0.9 or below -0.9 with the mean expression values of stage-specific genes.

Correlated genes	Correlation value
Ptpn1	1
Dnajc5	1
2810407C02Rik	1
Fam212b	1
Trmt13	1
Aplf	1
Eepd1	1
Pcsk7	1
Slirp	1
Nsa2	1
Mdp1	1
Commd5	1
Abcf3	1
Gtf2f1	1
Ly9	0.986
Ch25h	0.943
Chuk	0.943
Cstad	0.943
Dusp19	0.943
Lmo2	0.943
Emc7	0.943
Prpf18	0.943
Trdmt1	0.943
Rabl6	0.943
Zdhhc5	0.943
Dgkz	0.943
Opn3	0.943
Srp14	0.943
Snx5	0.943
Psma7	0.943
Prox1	0.943
Ndufb5	0.943
Ensa	0.943
Gdap2	0.943
Alg14	0.943
Commd2	0.943
Siah2	0.943
Atp5f1	0.943
Hs2st1	0.943
Prkacb	0.943
Mical1	0.943
Lepre1	0.943
Tmem234	0.943
Sdc3	0.943
Lzic	0.943
Pank4	0.943
Cdk11b	0.943
Aptx	0.943
Tstd2	0.943
Xpa	0.943
Bmpr2	0.943
Fam117b	0.943
Wdr78	0.943
Zmpste24	0.943
Stx12	0.943
Mtfr1l	0.943

Klhl17	0.943
Slc4a1ap	0.943
Tbc1d1	0.943
Arl6ip4	0.943
Bri3	0.943
Bud31	0.943
Preb	0.943
Stk11	0.943
Bloc1s4	0.943
1600002K03Rik	0.943
Sgcb	0.943
Srrd	0.943
Hilpda	0.943
Nt5dc3	0.943
Brk1	0.943
Pparg	0.943
Hnrnpf	0.943
Ndufa5	0.943
Chchd3	0.943
Kbtbd12	0.943
Zfyve20	0.943
Ccnd2	0.943
Lgi4	0.943
Rpl18	0.943
Orai3	0.943
Pex3	0.943
Zfp61	0.943
Rrp8	0.943
Rab8a	0.943
Ap1m1	0.943
Thap11	0.943
Afg3l1	0.943
Zdhhc7	0.943
Dpagt1	0.943
Faim	0.943
Usp4	0.943
Fdx1l	0.943
Usp3	0.943
Tmod3	0.943
Rasa2	0.943
1110059G10Rik	0.943
Iqsec2	0.943
Ccdc22	0.943
Mmgt1	0.943
Cetn2	0.943
Dnase1l1	0.943
Esyt1	0.943
Nxf7	0.943
Hccs	0.943
Rhot1	0.943
Atg4b	0.943
Pgs1	0.943
Erlec1	0.943
Ttc1	0.943
Cox10	0.943
Ccdc93	0.943
Glod4	0.943
Nme2	0.943
Gosr2	0.943
Fam104a	0.943

Cygb	0.943
Cant1	0.943
Isca2	0.943
Dlst	0.943
Gpatch2l	0.943
Tmem251	0.943
Ppp2r5c	0.943
Dld	0.943
Bag5	0.943
Gadd45g	0.943
Ptcd2	0.943
Bcl2l2	0.943
Ccdc25	0.943
Pibf1	0.943
Gmfb	0.943
Tbc1d4	0.943
Lmbrd2	0.943
Tef	0.943
Parvb	0.943
Slc25a32	0.943
Fam168b	0.943
Kansl3	0.943
Nfam1	0.943
Cdpf1	0.943
Nckap5l	0.943
Nudt16l1	0.943
Ece2	0.943
Cblb	0.943
Anks3	0.943
Gtf2h5	0.943
Clic1	0.943
Foxn2	0.943
Psmb1	0.943
D17Wsu104e	0.943
Ndufa10	0.943
Memo1	0.943
Myeov2	0.943
Napg	0.943
Tmx3	0.943
Kif5b	0.943
Slc39a6	0.943
4933408B17Rik	0.943
Ndufa2	0.943
Ndst1	0.943
Srgap2	0.943
Stambpl1	0.943
Tbc1d12	0.943
Zdhhc16	0.943
Sdhaf2	0.943
Endou	0.941
4930402F06Rik	0.928
Slc12a3	0.928
Gm3383	0.928
Zfp385b	-0.928
Pi15	-0.928
Gm6086	-0.928
Krt15	-0.928
Ribc2	-0.928
Dlgap1	-0.928
Zp3r	-0.928

Lrrc43	-0.941
Slc26a5	-0.941
Gpr101	-0.941
Serpina7	-0.941
Psd	-0.943
2310002J15Rik	-0.943
Prrg4	-0.943
Jag1	-0.943
Kcnk2	-0.943
Cr2	-0.943
Dennd2c	-0.943
Gipc2	-0.943
Ahdc1	-0.943
Rnf207	-0.943
Dnahc10	-0.943
Cux2	-0.943
Zfp956	-0.943
Prr19	-0.943
C030039L03Rik	-0.943
Rps16	-0.943
Slc7a10	-0.943
Fut1	-0.943
Me3	-0.943
Vwa3a	-0.943
Plekhb1	-0.943
Nfatc2ip	-0.943
Syce2	-0.943
Got1l1	-0.943
1700102P08Rik	-0.943
Lrrc1	-0.943
Sema3b	-0.943
Pim2	-0.943
Ccdc160	-0.943
Sox3	-0.943
Coro6	-0.943
Rpl19	-0.943
Itgb4	-0.943
Ranbp17	-0.943
Pttg1	-0.943
Pcgf2	-0.943
Mybph	-0.943
Nkx2-9	-0.943
Ccdc88c	-0.943
Srd5a1	-0.943
Ttc18	-0.943
Dlgap5	-0.943
Ndrg2	-0.943
Krt7	-0.943
Lynx1	-0.943
Eppk1	-0.943
Ahsg	-0.943
Fam3b	-0.943
Slc44a4	-0.943
Ihh	-0.943
Baiap3	-0.943
Ddr1	-0.943
Dtna	-0.943
Fosl1	-0.943
Il33	-0.943
Fam229a	-1

Rltpr	-1
Slc25a14	-1
Lrrc48	-1
Nacad	-1
Rps6ka5	-1
Ppara	-1
Spag6	-1

Table S19. 83 TFs which regulate 243 correlated genes.

TFs
Aes, Arid3a, Atf2, Atf4, Cebpb, Chd7, Cited2, Ctnnb1, Dmtf1, Ebf1, Egr1, Ehmt2, Elf5, Ep300, Esr1, Esr2, Esrra, Etv2, Etv4, Foxa2, Foxm1, Foxo1, Foxo3, Foxo4, Gata4, Hdac1, Hivep2, Hnf4a, Hoxa9, Jun, Klf2, Klf4, Klf6, Maf, Med1, Mnt, Msx2, Myc, Myod1, Ncoa3, Nfe2l2, Nfkb1, Nfya, Nfyb, Nfyc, Nr0b1, Nr0b2, Nr1i2, Nr1i3, Nr2f2, Nr3c1, Nrl, Pdx1, Per1, Pgr, Ppard, Ppargc1a, Prdm16, Rb1, Rbpj, Rela, Rora, Runx2, Satb1, Sirt1, Smad2, Smad3, Smad4, Snai1, Sox18, Sox2, Sox5, Sp1, Stat1, Stat3, Stat5a, Stat5b, Thra, Thrap3, Tnfaip3, Trp53, Xpc, Zfp423

Table S20: Enriched GO terms and KEGG pathways with adjusted p-values < 0.05 for the TFs involved in the cell fate process.

Enriched terms	count	adj. p-values
GO:0006351 transcription, DNA-templated	78	0
GO:0006355 regulation of transcription, DNA-templated	79	0
GO:0045944 positive regulation of transcription from RNA polymerase II promoter	65	0
GO:0045893 positive regulation of transcription, DNA-templated	53	0
GO:0000122 negative regulation of transcription from RNA polymerase II promoter	40	0
GO:0006357 regulation of transcription from RNA polymerase II promoter	29	0
GO:0045892 negative regulation of transcription, DNA-templated	31	0
GO:0043401 steroid hormone mediated signaling pathway	14	0
GO:0010628 positive regulation of gene expression	23	0
GO:0006366 transcription from RNA polymerase II promoter	16	0
GO:0001701 in utero embryonic development	17	0
GO:0010629 negative regulation of gene expression	15	0
GO:0008285 negative regulation of cell proliferation	16	0
GO:0048511 rhythmic process	11	0
GO:0042127 regulation of cell proliferation	13	0
GO:0007623 circadian rhythm	10	0
GO:0008284 positive regulation of cell proliferation	17	0
GO:0043066 negative regulation of apoptotic process	17	0
GO:0001889 liver development	9	0
GO:0048662 negative regulation of smooth muscle cell proliferation	7	0
GO:0043124 negative regulation of I-kappaB kinase/NF-kappaB signaling	7	0
GO:0043433 negative regulation of sequence-specific DNA binding transcription factor activity	8	0
GO:0043525 positive regulation of neuron apoptotic process	8	0
GO:0023019 signal transduction involved in regulation of gene expression	6	0
GO:0060548 negative regulation of cell death	8	0
GO:0034097 response to cytokine	8	0
GO:0070301 cellular response to hydrogen peroxide	7	0
GO:0048863 stem cell differentiation	6	0
GO:0030513 positive regulation of BMP signaling pathway	6	0
GO:0042789 mRNA transcription from RNA polymerase II promoter	5	0
GO:0019218 regulation of steroid metabolic process	4	0
GO:0009749 response to glucose	7	0
GO:0048661 positive regulation of smooth muscle cell proliferation	7	0
GO:0030154 cell differentiation	16	0
GO:0007507 heart development	10	0
GO:1902895 positive regulation of pri-miRNA transcription from RNA polymerase II promoter	5	0
GO:0042493 response to drug	11	0
GO:0003151 outflow tract morphogenesis	6	0
GO:0036302 atrioventricular canal development	4	0
GO:0035914 skeletal muscle cell differentiation	6	0
GO:1901215 negative regulation of neuron death	6	0
GO:0032355 response to estradiol	7	0
GO:0071456 cellular response to hypoxia	7	0
GO:0032922 circadian regulation of gene expression	6	0
GO:0051091 positive regulation of sequence-specific DNA binding transcription factor activity	7	0
GO:0071356 cellular response to tumor necrosis factor	7	0
GO:0071392 cellular response to estradiol stimulus	5	0
GO:0048617 embryonic foregut morphogenesis	4	0
GO:0010718 positive regulation of epithelial to mesenchymal transition	5	0.001
GO:0007179 transforming growth factor beta receptor signaling pathway	6	0.001
GO:0003198 epithelial to mesenchymal transition involved in endocardial cushion formation	4	0.001

GO:0060397 JAK-STAT cascade involved in growth hormone signaling pathway	4	0.001
GO:0060395 SMAD protein signal transduction	6	0.001
GO:0071347 cellular response to interleukin-1	6	0.001
GO:0007050 cell cycle arrest	6	0.001
GO:0007275 multicellular organism development	16	0.002
GO:0030518 intracellular steroid hormone receptor signaling pathway	4	0.002
GO:0071409 cellular response to cycloheximide	3	0.002
GO:0045599 negative regulation of fat cell differentiation	5	0.002
GO:0010468 regulation of gene expression	9	0.002
GO:0060065 uterus development	4	0.002
GO:0090090 negative regulation of canonical Wnt signaling pathway	6	0.003
GO:0032332 positive regulation of chondrocyte differentiation	4	0.003
GO:0007568 aging	7	0.003
GO:0035050 embryonic heart tube development	4	0.004
GO:0009952 anterior/posterior pattern specification	6	0.004
GO:0008584 male gonad development	6	0.004
GO:0007259 JAK-STAT cascade	4	0.004
GO:0009612 response to mechanical stimulus	5	0.004
GO:0034599 cellular response to oxidative stress	5	0.004
GO:0051098 regulation of binding	3	0.005
GO:0007219 Notch signaling pathway	6	0.006
GO:0030308 negative regulation of cell growth	6	0.006
GO:0001892 embryonic placenta development	4	0.006
GO:0030182 neuron differentiation	6	0.006
GO:0019827 stem cell population maintenance	5	0.006
GO:0045930 negative regulation of mitotic cell cycle	4	0.006
GO:0071499 cellular response to laminar fluid shear stress	3	0.007
GO:0030522 intracellular receptor signaling pathway	3	0.007
GO:2001171 positive regulation of ATP biosynthetic process	3	0.007
GO:0050680 negative regulation of epithelial cell proliferation	5	0.007
GO:0071222 cellular response to lipopolysaccharide	7	0.008
GO:0071407 cellular response to organic cyclic compound	5	0.008
GO:0043627 response to estrogen	5	0.008
GO:0035116 embryonic hindlimb morphogenesis	4	0.009
GO:0071345 cellular response to cytokine stimulus	4	0.009
GO:0045444 fat cell differentiation	5	0.009
GO:0019221 cytokine-mediated signaling pathway	6	0.01
GO:0001707 mesoderm formation	4	0.01
GO:0048589 developmental growth	4	0.01
GO:0030856 regulation of epithelial cell differentiation	3	0.011
GO:0007183 SMAD protein complex assembly	3	0.011
GO:0035115 embryonic forelimb morphogenesis	4	0.011
GO:0007369 gastrulation	4	0.011
GO:0060021 palate development	5	0.011
GO:0030509 BMP signaling pathway	5	0.012
GO:0050728 negative regulation of inflammatory response	5	0.012
GO:0060290 transdifferentiation	3	0.013
GO:0010224 response to UV-B	3	0.013
GO:0033077 T cell differentiation in thymus	4	0.013
GO:0014911 positive regulation of smooth muscle cell migration	4	0.013
GO:0060070 canonical Wnt signaling pathway	5	0.013
GO:0007492 endoderm development	4	0.014
GO:0043065 positive regulation of apoptotic process	8	0.014
GO:0030217 T cell differentiation	4	0.015
GO:0006915 apoptotic process	10	0.018
GO:0045429 positive regulation of nitric oxide biosynthetic process	4	0.018
GO:0060068 vagina development	3	0.018
GO:1990440 positive regulation of transcription from RNA polymerase II promoter in response to endoplasmic reticulum stress	3	0.018
GO:0035019 somatic stem cell population maintenance	4	0.019

GO:0032870 cellular response to hormone stimulus	4	0.02
GO:0045722 positive regulation of gluconeogenesis	3	0.021
GO:0001542 ovulation from ovarian follicle	3	0.021
GO:0016569 covalent chromatin modification	7	0.021
GO:0010942 positive regulation of cell death	4	0.021
GO:0001658 branching involved in ureteric bud morphogenesis	4	0.021
GO:0001501 skeletal system development	5	0.022
GO:0043923 positive regulation by host of viral transcription	3	0.024
GO:0060411 cardiac septum morphogenesis	3	0.024
GO:0070932 histone H3 deacetylation	3	0.024
GO:0051591 response to cAMP	4	0.024
GO:0051726 regulation of cell cycle	5	0.025
GO:0001649 osteoblast differentiation	5	0.026
GO:0045596 negative regulation of cell differentiation	5	0.026
GO:1901653 cellular response to peptide	3	0.026
GO:0060766 negative regulation of androgen receptor signaling pathway	3	0.026
GO:0045471 response to ethanol	5	0.029
GO:0045667 regulation of osteoblast differentiation	3	0.029
GO:0042981 regulation of apoptotic process	6	0.03
GO:0001756 somitogenesis	4	0.03
GO:0001541 ovarian follicle development	4	0.031
GO:0032526 response to retinoic acid	4	0.031
GO:0001706 endoderm formation	3	0.032
GO:0014912 negative regulation of smooth muscle cell migration	3	0.032
GO:0030324 lung development	5	0.033
GO:0009267 cellular response to starvation	4	0.035
GO:0071354 cellular response to interleukin-6	3	0.035
GO:0045670 regulation of osteoclast differentiation	3	0.035
GO:0035994 response to muscle stretch	3	0.035
GO:0003222 ventricular trabecula myocardium morphogenesis	3	0.035
GO:0001947 heart looping	4	0.037
GO:0002076 osteoblast development	3	0.039
GO:0071363 cellular response to growth factor stimulus	4	0.04
GO:0042593 glucose homeostasis	5	0.041
GO:0008283 cell proliferation	6	0.042
GO:0017015 regulation of transforming growth factor beta receptor signaling pathway	3	0.042
GO:0042733 embryonic digit morphogenesis	4	0.042
GO:0045669 positive regulation of osteoblast differentiation	4	0.045
GO:0001570 vasculogenesis	4	0.045
mmu05166:HTLV-I infection	18	0
mmu05161:Hepatitis B	14	0
mmu04919:Thyroid hormone signaling pathway	12	0
mmu05200:Pathways in cancer	18	0
mmu04917:Prolactin signaling pathway	10	0
mmu05321:Inflammatory bowel disease (IBD)	9	0
mmu05212:Pancreatic cancer	9	0
mmu05220:Chronic myeloid leukemia	9	0
mmu05203:Viral carcinogenesis	13	0
mmu05202:Transcriptional misregulation in cancer	11	0
mmu04068:FoxO signaling pathway	10	0
mmu05169:Epstein-Barr virus infection	10	0
mmu05221:Acute myeloid leukemia	7	0
mmu05215:Prostate cancer	8	0
mmu05210:Colorectal cancer	7	0
mmu04110:Cell cycle	8	0
mmu05162:Measles	8	0
mmu04550:Signaling pathways regulating pluripotency of stem cells	8	0
mmu04668:TNF signaling pathway	7	0.001
mmu04520:Adherens junction	6	0.001

mmu05152:Tuberculosis	8	0.002
mmu04350:TGF-beta signaling pathway	6	0.002
mmu05030:Cocaine addiction	5	0.002
mmu04310:Wnt signaling pathway	7	0.003
mmu04915:Estrogen signaling pathway	6	0.003
mmu04922:Glucagon signaling pathway	6	0.004
mmu04722:Neurotrophin signaling pathway	6	0.008
mmu05222:Small cell lung cancer	5	0.014
mmu04630:Jak-STAT signaling pathway	6	0.016
mmu05168:Herpes simplex infection	7	0.016
mmu04390:Hippo signaling pathway	6	0.018
mmu05213:Endometrial cancer	4	0.023
mmu05142:Chagas disease (American trypanosomiasis)	5	0.024
mmu05164:Influenza A	6	0.028
mmu04931:Insulin resistance	5	0.028
mmu04010:MAPK signaling pathway	7	0.032
mmu05140:Leishmaniasis	4	0.035
mmu05031:Amphetamine addiction	4	0.039
mmu04152:AMPK signaling pathway	5	0.041
mmu04062:Chemokine signaling pathway	6	0.042
mmu04920:Adipocytokine signaling pathway	4	0.044
mmu04950:Maturity onset diabetes of the young	3	0.045
mmu05160:Hepatitis C	5	0.046
mmu05216:Thyroid cancer	3	0.049

Table S21. Cell-specific genes governing blood cell differentiation.

HSC	Gmpr, Atp6v1f, Prkacb, Eif1b, Gkap1, Rhobtb3, Arsk, 2700060E02Rik, Angpt1, Rpl36-ps2, Sirt3, Stau2, Smyd2, Nudt2, Hint2, Pomgnt1, Gpr125, Fkbp9, Prdm5, Sh2b1, Smad1, Armcx1, Pkd2, Tle6, Tmtc2, Mycn, Dsel, Fnbp1l, Spns2, Ttc3, Pla2g6, Osbpl1a, Megf8, Cxxc5, H1f0, Bex1, Insl6, Parp4, Fbxo6, Rpl36, Gm10036, Pstk, Gm13611, Rpl36-ps3, Gm5614, Zfp820, Gm10288, Gm10297, Gm10704, Snord65, Ipo11, Zfp664, 2410006H16Rik, C920006O11Rik, Unannotated, Gm4604
MPP1	Gmpr, Atp6v1f, Prkacb, Eif1b, Gkap1, Rhobtb3, Arsk, 2700060E02Rik, Angpt1, Rpl36-ps2, Sirt3, Stau2, Smyd2, Nudt2, Hint2, Pomgnt1, Gpr125, Fkbp9, Prdm5, Sh2b1, Smad1, Armcx1, Pkd2, Tle6, Tmtc2, Mycn, Dsel, Fnbp1l, Spns2, Ttc3, Pla2g6, Osbpl1a, Megf8, Cxxc5, H1f0, Bex1, Insl6, Parp4, Fbxo6, Rpl36, Gm10036, Pstk, Gm13611, Rpl36-ps3, Gm5614, Zfp820, Gm10288, Gm10297, Gm10704, Snord65, Ipo11, Zfp664, 2410006H16Rik, C920006O11Rik, Unannotated, Gm4604
MPP2	Smarcb1, Puf60, Pex6, Btbd2, Homer3, Rcn1, Vars, Zfp775, Gatad1, Arid1a, Vps72, Git1, Cd34, Nt5c3l, Pfn1, Mtap1s, Zfp687, Hmg20b, Zkscan17, Sumo2, Tsen54, Kat2a, Txndc15, Btd, Ebpl, Dpysl2, Slc39a14, Fbxl6, Smarcd1, Nfya, Mllt1, Zfp523, Tap2, Rbm22, Frmd8, Drap1, Fbxl15, Obfc2b, Slco3a1, Zfp282, Stk11ip, Rbck1, Spaca1, Pum1, Rnf6, Anxa4, Etv6, Zmym3, Dctd, Rbl2, Ntpcr, Fbxo21, BC016579, Specc1l, 2610301G19Rik, 2410018M08Rik, Eef2k, Fam69b, Zfp113, Atg9b, Sgsm2, AW209491, Bahcc1, Cul9, Crocc, Pdcd7, Slc35f2, Gm5867, Otos, Cep68, Chtf8, Spn, Rasal3, Opa3, Tmem106c, Suclg1, Adnp2, Pcnxl3, Nfic, Pard6g, Grlf1, Fiz1, Ncs1, Zfp512, Hspbp1, Gpn1, Wdr6, Ssbp4, Spint2, Samd1, Gm9836, Smt3h2-ps4, Gm13077, Smt3h2-ps, Gm13430, Gm10241, Gm12335, Gm13690, Sco2
CLP	Mcts1, Tgfb1, Nudt14, Med29, Pafah1b3, Trim28, Saal1, Tjap1, ORF61, 2810408A11Rik, Fdxr, Dalrd3, Ube2d1, Mgat1, Acaca, Prkar1a, Map2k6, Mta1, Slc4a7, Zhx1, Srl, Tbc1d5, Anks1, Pdgfrb, Fasn, Mrpl43, Chmp6, Gpsm1, Arhgef2, Zfp362, Ncdn, Mtap7d1, Ociad2, G3bp2, Ints1, Cux1, Gnb2, 2010007H12Rik, Casp2, 2400001E08Rik, 2400003C14Rik, Katnb1, Gtl3, Pgls, Csk, Nt5m, Ttyh2, Plekhj1, Cnot3, Apex1, Gatad2a, H2-DMa, Ankrd13b, Armc10, Tbc1d19, Azi1, Klhdc5, Mapk6, A030009H04Rik, Irf2bp1, Zfp688, Gm5455, Fam105b, Zfp84, Zfp740, Gm7204, Pdp2, 8430408G22Rik, Zfp219, Gm9844, Zfp191, Ppp6r1, Cfl1, Zbtb25, Txnl4a, Hist1h3f, Ube2nl, Akap17b, Zfp74, Snrpa, Btbd3, Jakmip1, Hist1h3e, Runx3, Gm5145, Apcdd1, Sox4, Tmsb10, Hist2h3c2-ps, Gm5510, Gm3788
CMP	Akt1, Keap1, Exosc10, Dync1h1, Fkbp8, Ttc15, Csnk1e, 2310016M24Rik, Rrp36, Nudt3, Slc29a2, Acadl, Abca2, Pcna, Arfgap1, Nudc, Cops7a, Psmb10, Ubash3b, Bud13, Grinl1a, Pebp1, Mif, Vps18, Sigmar1, Rreb1, Dag1, Gm9769, Ascc1, Ctdspl, Akap4, Gm16379, Impdh2, Med18, Pcna-ps2, Gm15210, Gm10819, Gm10169, Purb, Gcom1, Grinl1a
MEP	Gna12, Uck1, Khsrp, Psmd3, Snrpd3, 1300001I01Rik, Mccc2, Slc25a30, Parn, Psmg1, Twsg1, Rcl1, Acsl5, Arpc1a, Ino80e, Bag3, St3gal4, Dhcr24, Josd2, Wdr34, BC048355, Cpsf2, Bag2, Unannotated, Ptges3, Kti12, Gm4482
GMP	Top1mt, Mfsd10, Calr, Gpr108, Slc5a6, Kdsr, Poc1b, Slc36a1, Pfkl, Sel1l, Cdca7l, Mbnl2, Pycrl, Psat1, Tmem2, Rtn3, P4hb, Tagln2, Lamtor2, Pex11b, Tmub1, Emilin1, Pcyox1, Msn, Spg21, Prkar2a, Smyd5, G6pc3, Bbs9, Cyb561d2, 9430023L20Rik, Igsf8, Ccbl2, Hlcs, Thtpa, Arf1, Mlec, Gapdh, Gm5506, Taf1b, Eno1, Gm13699, Cenpb, Gm12070, Gm10284, Gm10290, Gm10358, Gm10359, Gm10481, Gm10566, Grk6, Unannotated, Gm2574, Gm5559, Gm2606, Gm4609, Gm12537, Gm12033, Gm3272, Gm3839, Gm5138, Gm12671, Gm13464, Gm14760, Gm12286, Gm13882, Gm14078, Gm13394
CD4	Hbs1l, Wdr37, Ippk, Vegfa, Uhmk1, Lass6, Eri2, Gla, Ppp2r1b, Zdhhc15, Zfp418, Pmch, Rnf38, Hace1, Skiv2l, Mis12, 2700046A07Rik, Gm5424, Pigm, Ostb, Mcm9, Lrrc40, Ttc9c, Wipf1, Alg10b, Ass1, Rad54b, Gm6743
CD8	37135, Myg1, Smg5, Dyrk1b, Atp5d, Bax, Ulk2, Ndufs3, Fbxw9, Psmb1, Ttll12, Trpv2, Ncln, Cenpo, Utp23, L3mbtl2, Atp13a3, Lnpep, Chd1, Spsb3, Cep192, Pcyt2, Imp4, Olfm1, Nat10, Usp50, Gpatch3, Slc25a33, Ccr9, Pycard, Piga, Fam192a, Acad8, Arcn1, Car12, Ddx19b, Ndufa3, Tbcel, Bbs7, Polr2l, Pigk, Hlx, Prpf18, Ankrd6, Tmtc4, Cox6a1, BC003266, Baiap3, Selplg, Rasl11b, Gfod1, Sphk2, Bcas3, Gm8355, Atp8b4, Gm7099, Nsl1, Snrnp70, Pepd, Gmppb, Capza1, Gm10356, Dda1, Gm10774, Serf2, Snord14c, Gm15459, Gm12251, Zfp712
BCell	Narf, Haao, Spg7, Tcirg1, Fam98a, Senp3, Cnp, Dynll1, Nuak2, Tnfrsf13b, 5930434B04Rik, Ctsz, Ttpal, Myo1c, Cyb5r3, Mtmr4, Gipc1, Rtn4ip1, Rel, Rufy1, Fbxl20, Ccnk, Ddx41, Lcp1, Fam49b, Txndc11, B3gnt5, Son, Fmnl3, Med20, Dcp2, Nedd4l, Pik3ap1, Btbd1, Entpd7, Cwf19l1, Hspd1, Il18rap, Nhej1, Abl1, Strbp, Slc12a6, Ubox5, Ivd, Ptpn1, Spata5, Serp1, Tmem144, Snx7, Kcnq5, Dclk2, Nfx1, Dcaf12, Pex10, Slc15a4, Bcl7a, Kdm2b, Ankle2, Cd37, Fbxl19, Agpat6, Eif2ak3, Lactb, Cntrob, Chst2, Tesk2, Setd5, Wdr5b, Morc2a, Bmp2k, Map4k5, Fam167a, Uvrag, 2210018M11Rik, 5031439G07Rik, Jmjd4, Map4k1, Depdc5, Ints7, Mavs, Ccdc50, Socs7, Mesdc2, Jarid2, Foxk2, BC017643, Rere, 9130011E15Rik, Rcsd1, Rfwd2, Phf8, H2-Ob, 1110018G07Rik, Agps, Ube2q1, Rnf214, Tmem194b, Tlr7, Gemin7, Setd2, Cdk5r1, Gm6206, Dcaf7, Nup160, Kctd14, Tubgcp6, Kdm2a, Mettl4, Ldoc1l, Atp5sl, Yipf1, Gm12141, Grb2, 9430015G10Rik, 1700052N19Rik, Zfyve19, Pde7a, Fgfr1op, Fam82a2, Ncf4, 4933437F05Rik, Ranbp6, Ighm, 4930420K17Rik, Gm12397, Shkbp1, Gm16199, Gm6788
Eryth	Fbxo7, Odc1, Ogdh, Tmem107, Ank, Srbd1, Rhot2, Pask, Ankrd54, 2510012J08Rik, Fbxo45, Tubb2c, Mcart1, Ankle1, Zfp280b, Nrf1, Ext1, Dazap1, Gm9115, Gm14928, 2810453I06Rik
Granu	Dhrs1, Nampt, Nin, Serp1, Ugdh, Srd5a3, Gnai2, 2410016O06Rik, Flot2, Pgk1, Ethe1, Pgk1-rs7, Gm5537, 3000002C10Rik, Gm17510
Mono	Elmo2, Poc1a, Trak1, Fam113a

Table S22. Lineage-specific correlated genes including the TFs (second column). Number of TFs that regulate the correlated genes inferred from TRRUST database (third column). The number of correlated genes that are the targets of TFs in the third column (fourth column).

Cell line	# Correlated genes (# included TFs)	# TF	# Correlated targets of TFs
CD4	1102 (47)	163	93
CD8	1155 (54)	182	101
Bcell	1248 (52)	173	107
Eryth	517 (25)	128	32
Granu	533 (33)	138	67
Mono	232 (14)	56	26

Table S23: Enriched GO terms and KEGG pathways with adjusted p-values < 0.05 for the TFs involved in the CD4 T-cell fate process.

Enriched terms	count	adj. p-values
GO:0006351 transcription, DNA-templated	148	0
GO:0006355 regulation of transcription, DNA-templated	153	0
GO:0045944 positive regulation of transcription from RNA polymerase II promoter	125	0
GO:0045893 positive regulation of transcription, DNA-templated	94	0
GO:0000122 negative regulation of transcription from RNA polymerase II promoter	88	0
GO:0045892 negative regulation of transcription, DNA-templated	55	0
GO:0006357 regulation of transcription from RNA polymerase II promoter	44	0
GO:0010628 positive regulation of gene expression	38	0
GO:0008285 negative regulation of cell proliferation	33	0
GO:0007275 multicellular organism development	47	0
GO:0030154 cell differentiation	41	0
GO:0006366 transcription from RNA polymerase II promoter	20	0
GO:0043066 negative regulation of apoptotic process	33	0
GO:0010468 regulation of gene expression	26	0
GO:0008284 positive regulation of cell proliferation	32	0
GO:0042127 regulation of cell proliferation	23	0
GO:1902895 positive regulation of pri-miRNA transcription from RNA polymerase II promoter	11	0
GO:0030182 neuron differentiation	18	0
GO:0007507 heart development	22	0
GO:0001701 in utero embryonic development	23	0
GO:0048511 rhythmic process	17	0
GO:0035914 skeletal muscle cell differentiation	13	0
GO:0045165 cell fate commitment	14	0
GO:0003151 outflow tract morphogenesis	12	0
GO:0010629 negative regulation of gene expression	20	0
GO:0042493 response to drug	22	0
GO:0007623 circadian rhythm	14	0
GO:0045669 positive regulation of osteoblast differentiation	12	0
GO:0043401 steroid hormone mediated signaling pathway	11	0
GO:0007179 transforming growth factor beta receptor signaling pathway	12	0
GO:0034097 response to cytokine	12	0
GO:0021983 pituitary gland development	9	0
GO:0030097 hemopoiesis	12	0
GO:0048663 neuron fate commitment	9	0
GO:0001889 liver development	12	0
GO:0045665 negative regulation of neuron differentiation	11	0
GO:0090090 negative regulation of canonical Wnt signaling pathway	12	0
GO:0007492 endoderm development	9	0
GO:0002052 positive regulation of neuroblast proliferation	8	0
GO:0009887 organ morphogenesis	12	0
GO:0060021 palate development	11	0
GO:0009952 anterior/posterior pattern specification	12	0
GO:0048469 cell maturation	9	0
GO:0035019 somatic stem cell population maintenance	9	0
GO:0060216 definitive hemopoiesis	7	0
GO:0035116 embryonic hindlimb morphogenesis	8	0
GO:0001503 ossification	11	0
GO:0050680 negative regulation of epithelial cell proliferation	10	0
GO:0007219 Notch signaling pathway	12	0
GO:0010718 positive regulation of epithelial to mesenchymal transition	8	0
GO:0035162 embryonic hemopoiesis	7	0
GO:0030279 negative regulation of ossification	7	0
GO:0071407 cellular response to organic cyclic compound	10	0
GO:0035115 embryonic forelimb morphogenesis	8	0

GO:0030513 positive regulation of BMP signaling pathway	8	0
GO:0045666 positive regulation of neuron differentiation	11	0
GO:0002051 osteoblast fate commitment	5	0
GO:0060412 ventricular septum morphogenesis	8	0
GO:0001501 skeletal system development	11	0
GO:0051216 cartilage development	10	0
GO:0043065 positive regulation of apoptotic process	17	0
GO:0030217 T cell differentiation	8	0
GO:0030900 forebrain development	10	0
GO:0030509 BMP signaling pathway	10	0
GO:0009612 response to mechanical stimulus	9	0
GO:0042733 embryonic digit morphogenesis	9	0
GO:0001892 embryonic placenta development	7	0
GO:0001666 response to hypoxia	13	0
GO:0030324 lung development	11	0
GO:0043524 negative regulation of neuron apoptotic process	12	0
GO:2000144 positive regulation of DNA-templated transcription, initiation	5	0
GO:0045599 negative regulation of fat cell differentiation	8	0
GO:0030218 erythrocyte differentiation	8	0
GO:0001657 ureteric bud development	8	0
GO:0001837 epithelial to mesenchymal transition	7	0
GO:0050767 regulation of neurogenesis	7	0
GO:0042789 mRNA transcription from RNA polymerase II promoter	6	0
GO:0007183 SMAD protein complex assembly	5	0
GO:0060395 SMAD protein signal transduction	9	0
GO:0007568 aging	12	0
GO:0051726 regulation of cell cycle	10	0
GO:0032526 response to retinoic acid	8	0
GO:0030879 mammary gland development	7	0
GO:0048701 embryonic cranial skeleton morphogenesis	7	0
GO:0045595 regulation of cell differentiation	7	0
GO:0032332 positive regulation of chondrocyte differentiation	6	0
GO:0001947 heart looping	8	0
GO:0042981 regulation of apoptotic process	12	0
GO:0003148 outflow tract septum morphogenesis	6	0
GO:0009880 embryonic pattern specification	6	0
GO:0001822 kidney development	10	0
GO:0043525 positive regulation of neuron apoptotic process	8	0
GO:0009954 proximal/distal pattern formation	6	0
GO:0042472 inner ear morphogenesis	8	0
GO:0003198 epithelial to mesenchymal transition involved in endocardial cushion formation	5	0
GO:0001658 branching involved in ureteric bud morphogenesis	7	0
GO:0051591 response to cAMP	7	0
GO:0006338 chromatin remodeling	8	0
GO:0061419 positive regulation of transcription from RNA polymerase II promoter in response to hypoxia	4	0
GO:0051098 regulation of binding	4	0
GO:0007050 cell cycle arrest	8	0
GO:0001649 osteoblast differentiation	9	0
GO:0045596 negative regulation of cell differentiation	9	0
GO:0043388 positive regulation of DNA binding	6	0
GO:2000679 positive regulation of transcription regulatory region DNA binding	5	0
GO:0043523 regulation of neuron apoptotic process	6	0
GO:0070301 cellular response to hydrogen peroxide	7	0
GO:0010332 response to gamma radiation	6	0
GO:0050728 negative regulation of inflammatory response	8	0
GO:0007519 skeletal muscle tissue development	7	0
GO:0048485 sympathetic nervous system development	5	0
GO:0003203 endocardial cushion morphogenesis	5	0

GO:0048596 embryonic camera-type eye morphogenesis	5	0
GO:0048589 developmental growth	6	0
GO:0071773 cellular response to BMP stimulus	6	0
GO:0060070 canonical Wnt signaling pathway	8	0
GO:0001764 neuron migration	9	0
GO:0030308 negative regulation of cell growth	9	0
GO:0002076 osteoblast development	5	0
GO:0032922 circadian regulation of gene expression	7	0
GO:0030326 embryonic limb morphogenesis	7	0
GO:0048662 negative regulation of smooth muscle cell proliferation	6	0
GO:0017015 regulation of transforming growth factor beta receptor signaling pathway	5	0
GO:0071363 cellular response to growth factor stimulus	7	0
GO:0030219 megakaryocyte differentiation	4	0
GO:0003180 aortic valve morphogenesis	4	0
GO:0033077 T cell differentiation in thymus	6	0
GO:0045787 positive regulation of cell cycle	6	0.001
GO:0008283 cell proliferation	11	0.001
GO:0045648 positive regulation of erythrocyte differentiation	5	0.001
GO:0043433 negative regulation of sequence-specific DNA binding transcription factor activity	7	0.001
GO:0030858 positive regulation of epithelial cell differentiation	4	0.001
GO:0036302 atrioventricular canal development	4	0.001
GO:0001568 blood vessel development	7	0.001
GO:0023019 signal transduction involved in regulation of gene expression	5	0.001
GO:0045597 positive regulation of cell differentiation	6	0.001
GO:0002062 chondrocyte differentiation	6	0.001
GO:0071158 positive regulation of cell cycle arrest	5	0.001
GO:0043966 histone H3 acetylation	6	0.001
GO:0050679 positive regulation of epithelial cell proliferation	7	0.001
GO:0048844 artery morphogenesis	5	0.001
GO:0009749 response to glucose	7	0.001
GO:0009953 dorsal/ventral pattern formation	6	0.001
GO:0045444 fat cell differentiation	7	0.001
GO:0045662 negative regulation of myoblast differentiation	5	0.001
GO:0060548 negative regulation of cell death	7	0.001
GO:0071347 cellular response to interleukin-1	7	0.001
GO:0030099 myeloid cell differentiation	5	0.002
GO:0042752 regulation of circadian rhythm	6	0.002
GO:0045766 positive regulation of angiogenesis	8	0.002
GO:0045603 positive regulation of endothelial cell differentiation	4	0.002
GO:0048667 cell morphogenesis involved in neuron differentiation	4	0.002
GO:0071222 cellular response to lipopolysaccharide	10	0.002
GO:0071542 dopaminergic neuron differentiation	5	0.002
GO:0032570 response to progesterone	5	0.002
GO:0045668 negative regulation of osteoblast differentiation	6	0.002
GO:1901215 negative regulation of neuron death	6	0.002
GO:0051569 regulation of histone H3-K4 methylation	4	0.002
GO:0008015 blood circulation	4	0.002
GO:0006978 DNA damage response, signal transduction by p53 class mediator resulting in transcription of p21 class mediator	4	0.002
GO:0060391 positive regulation of SMAD protein import into nucleus	4	0.002
GO:0050821 protein stabilization	8	0.003
GO:0042771 intrinsic apoptotic signaling pathway in response to DNA damage by p53 class mediator	5	0.003
GO:0007389 pattern specification process	6	0.003
GO:0043967 histone H4 acetylation	5	0.003
GO:0000060 protein import into nucleus, translocation	5	0.003
GO:2000678 negative regulation of transcription regulatory region DNA binding	4	0.003
GO:0031065 positive regulation of histone deacetylation	4	0.003

GO:0042060 wound healing	7	0.003
GO:0031016 pancreas development	5	0.003
GO:0071345 cellular response to cytokine stimulus	5	0.003
GO:0071560 cellular response to transforming growth factor beta stimulus	6	0.003
GO:0048863 stem cell differentiation	5	0.004
GO:0008544 epidermis development	6	0.004
GO:0002064 epithelial cell development	4	0.004
GO:0043586 tongue development	4	0.004
GO:0003256 regulation of transcription from RNA polymerase II promoter involved in myocardial precursor cell differentiation	3	0.004
GO:0010871 negative regulation of receptor biosynthetic process	3	0.004
GO:0007517 muscle organ development	6	0.004
GO:0042475 odontogenesis of dentin-containing tooth	6	0.004
GO:0031069 hair follicle morphogenesis	5	0.004
GO:0003007 heart morphogenesis	6	0.004
GO:0071300 cellular response to retinoic acid	6	0.004
GO:0010033 response to organic substance	6	0.004
GO:0007369 gastrulation	5	0.004
GO:0045786 negative regulation of cell cycle	5	0.004
GO:0061029 eyelid development in camera-type eye	4	0.004
GO:0035909 aorta morphogenesis	4	0.004
GO:0007346 regulation of mitotic cell cycle	5	0.005
GO:0002053 positive regulation of mesenchymal cell proliferation	5	0.005
GO:0045747 positive regulation of Notch signaling pathway	5	0.005
GO:0030501 positive regulation of bone mineralization	5	0.005
GO:0055007 cardiac muscle cell differentiation	5	0.005
GO:0071456 cellular response to hypoxia	7	0.005
GO:0035729 cellular response to hepatocyte growth factor stimulus	4	0.005
GO:0032496 response to lipopolysaccharide	9	0.006
GO:0051091 positive regulation of sequence-specific DNA binding transcription factor activity	7	0.006
GO:0019827 stem cell population maintenance	6	0.006
GO:0030901 midbrain development	5	0.006
GO:0048568 embryonic organ development	5	0.006
GO:0060038 cardiac muscle cell proliferation	4	0.006
GO:0030224 monocyte differentiation	4	0.006
GO:0035994 response to muscle stretch	4	0.006
GO:0014902 myotube differentiation	4	0.006
GO:0003222 ventricular trabecula myocardium morphogenesis	4	0.006
GO:0050678 regulation of epithelial cell proliferation	4	0.006
GO:0045670 regulation of osteoclast differentiation	4	0.006
GO:0003266 regulation of secondary heart field cardioblast proliferation	3	0.006
GO:0032909 regulation of transforming growth factor beta2 production	3	0.006
GO:0060364 frontal suture morphogenesis	3	0.006
GO:0031018 endocrine pancreas development	5	0.007
GO:0008584 male gonad development	7	0.007
GO:0060037 pharyngeal system development	4	0.007
GO:0001709 cell fate determination	4	0.007
GO:0045598 regulation of fat cell differentiation	4	0.007
GO:0048565 digestive tract development	5	0.007
GO:0009791 post-embryonic development	7	0.007
GO:0043627 response to estrogen	6	0.007
GO:0030220 platelet formation	4	0.008
GO:0060065 uterus development	4	0.008
GO:0048706 embryonic skeletal system development	5	0.008
GO:0030539 male genitalia development	4	0.009
GO:0030318 melanocyte differentiation	4	0.009
GO:0048661 positive regulation of smooth muscle cell proliferation	6	0.01
GO:0034644 cellular response to UV	5	0.01
GO:0001714 endodermal cell fate specification	3	0.01

GO:0070345 negative regulation of fat cell proliferation	3	0.01
GO:0003211 cardiac ventricle formation	3	0.01
GO:0038061 NIK/NF-kappaB signaling	3	0.01
GO:0010742 macrophage derived foam cell differentiation	3	0.01
GO:0001755 neural crest cell migration	5	0.012
GO:0043011 myeloid dendritic cell differentiation	4	0.012
GO:0031668 cellular response to extracellular stimulus	4	0.012
GO:0030216 keratinocyte differentiation	6	0.012
GO:0006954 inflammatory response	11	0.014
GO:0060045 positive regulation of cardiac muscle cell proliferation	4	0.014
GO:0035050 embryonic heart tube development	4	0.014
GO:0001708 cell fate specification	4	0.014
GO:0021915 neural tube development	5	0.014
GO:0007283 spermatogenesis	12	0.014
GO:0043353 enucleate erythrocyte differentiation	3	0.014
GO:0042634 regulation of hair cycle	3	0.014
GO:0044336 canonical Wnt signaling pathway involved in negative regulation of apoptotic process	3	0.014
GO:1990314 cellular response to insulin-like growth factor stimulus	3	0.014
GO:0071559 response to transforming growth factor beta	3	0.014
GO:0048505 regulation of timing of cell differentiation	3	0.014
GO:0014842 regulation of skeletal muscle satellite cell proliferation	3	0.014
GO:0072602 interleukin-4 secretion	3	0.014
GO:0030902 hindbrain development	4	0.015
GO:0071375 cellular response to peptide hormone stimulus	4	0.015
GO:0048646 anatomical structure formation involved in morphogenesis	4	0.015
GO:0009987 cellular process	4	0.015
GO:0048705 skeletal system morphogenesis	5	0.017
GO:0097150 neuronal stem cell population maintenance	4	0.017
GO:0006974 cellular response to DNA damage stimulus	12	0.017
GO:0048704 embryonic skeletal system morphogenesis	5	0.018
GO:0048538 thymus development	5	0.018
GO:0042421 norepinephrine biosynthetic process	3	0.019
GO:0060261 positive regulation of transcription initiation from RNA polymerase II promoter	3	0.019
GO:0032808 lacrimal gland development	3	0.019
GO:0071599 otic vesicle development	3	0.019
GO:0002467 germinal center formation	3	0.019
GO:0009913 epidermal cell differentiation	3	0.019
GO:0034616 response to laminar fluid shear stress	3	0.019
GO:0010944 negative regulation of transcription by competitive promoter binding	3	0.019
GO:0009629 response to gravity	3	0.019
GO:0071316 cellular response to nicotine	3	0.019
GO:0003179 heart valve morphogenesis	3	0.019
GO:1901522 positive regulation of transcription from RNA polymerase II promoter involved in cellular response to chemical stimulus	3	0.019
GO:0007399 nervous system development	11	0.025
GO:0097421 liver regeneration	4	0.025
GO:0045930 negative regulation of mitotic cell cycle	4	0.025
GO:0014807 regulation of somitogenesis	3	0.025
GO:0003139 secondary heart field specification	3	0.025
GO:0032355 response to estradiol	6	0.025
GO:0030335 positive regulation of cell migration	8	0.027
GO:0007569 cell aging	4	0.027
GO:0042517 positive regulation of tyrosine phosphorylation of Stat3 protein	4	0.027
GO:0021984 adenohypophysis development	3	0.031
GO:0003208 cardiac ventricle morphogenesis	3	0.031
GO:0060290 transdifferentiation	3	0.031
GO:0061549 sympathetic ganglion development	3	0.031
GO:0003309 type B pancreatic cell differentiation	3	0.031

GO:0048146 positive regulation of fibroblast proliferation	5	0.031
GO:0016569 covalent chromatin modification	9	0.031
GO:0048839 inner ear development	5	0.032
GO:0006461 protein complex assembly	5	0.032
GO:0060349 bone morphogenesis	4	0.032
GO:0071392 cellular response to estradiol stimulus	4	0.035
GO:0070166 enamel mineralization	3	0.037
GO:0048709 oligodendrocyte differentiation	4	0.037
GO:0000165 MAPK cascade	5	0.037
GO:0009617 response to bacterium	4	0.04
GO:0042551 neuron maturation	3	0.044
GO:0031571 mitotic G1 DNA damage checkpoint	3	0.044
GO:0048708 astrocyte differentiation	3	0.044
GO:0061180 mammary gland epithelium development	3	0.044
GO:0048617 embryonic foregut morphogenesis	3	0.044
mmu05202:Transcriptional misregulation in cancer	27	0
mmu05166:HTLV-I infection	30	0
mmu05161:Hepatitis B	24	0
mmu05200:Pathways in cancer	33	0
mmu04350:TGF-beta signaling pathway	15	0
mmu05212:Pancreatic cancer	12	0
mmu05203:Viral carcinogenesis	19	0
mmu05220:Chronic myeloid leukemia	12	0
mmu04550:Signaling pathways regulating pluripotency of stem cells	15	0
mmu04110:Cell cycle	14	0
mmu04380:Osteoclast differentiation	14	0
mmu05215:Prostate cancer	12	0
mmu05321:Inflammatory bowel disease (IBD)	10	0
mmu04919:Thyroid hormone signaling pathway	12	0
mmu05210:Colorectal cancer	9	0
mmu05221:Acute myeloid leukemia	8	0
mmu04024:cAMP signaling pathway	13	0
mmu04310:Wnt signaling pathway	11	0
mmu05211:Renal cell carcinoma	8	0
mmu04068:FoxO signaling pathway	10	0
mmu05169:Epstein-Barr virus infection	10	0.001
mmu04668:TNF signaling pathway	9	0.001
mmu05222:Small cell lung cancer	8	0.001
mmu04390:Hippo signaling pathway	10	0.001
mmu05219:Bladder cancer	6	0.001
mmu05205:Proteoglycans in cancer	11	0.002
mmu04917:Prolactin signaling pathway	7	0.002
mmu04330:Notch signaling pathway	6	0.002
mmu05168:Herpes simplex infection	11	0.002
mmu04931:Insulin resistance	8	0.003
mmu05140:Leishmaniasis	6	0.007
mmu04915:Estrogen signaling pathway	7	0.008
mmu05218:Melanoma	6	0.009
mmu05162:Measles	8	0.009
mmu04662:B cell receptor signaling pathway	6	0.009
mmu05142:Chagas disease (American trypanosomiasis)	7	0.009
mmu05152:Tuberculosis	9	0.009
mmu04520:Adherens junction	6	0.009
mmu05133:Pertussis	6	0.01
mmu05206:MicroRNAs in cancer	11	0.013
mmu05030:Cocaine addiction	5	0.013
mmu04320:Dorso-ventral axis formation	4	0.013
mmu05016:Huntington's disease	9	0.015
mmu05216:Thyroid cancer	4	0.019
mmu05223:Non-small cell lung cancer	5	0.019

mmu04010:MAPK signaling pathway	10	0.019
mmu05164:Influenza A	8	0.022
mmu04710:Circadian rhythm	4	0.022
mmu05160:Hepatitis C	7	0.026
mmu04916:Melanogenesis	6	0.027
mmu04922:Glucagon signaling pathway	6	0.027
mmu04620:Toll-like receptor signaling pathway	6	0.028
mmu04660:T cell receptor signaling pathway	6	0.028
mmu04066:HIF-1 signaling pathway	6	0.029
mmu05145:Toxoplasmosis	6	0.031
mmu04630:Jak-STAT signaling pathway	7	0.031

Table S24: Enriched GO terms and KEGG pathways with adjusted p-values < 0.05 for the TFs involved in the CD8 T-cell fate process.

Enriched terms	count	adj. p-values
GO:0006351 transcription, DNA-templated	164	0
GO:0006355 regulation of transcription, DNA-templated	171	0
GO:0045944 positive regulation of transcription from RNA polymerase II promoter	133	0
GO:0045893 positive regulation of transcription, DNA-templated	102	0
GO:0000122 negative regulation of transcription from RNA polymerase II promoter	100	0
GO:0045892 negative regulation of transcription, DNA-templated	65	0
GO:0006357 regulation of transcription from RNA polymerase II promoter	51	0
GO:0010628 positive regulation of gene expression	40	0
GO:0007275 multicellular organism development	55	0
GO:0008285 negative regulation of cell proliferation	35	0
GO:0030154 cell differentiation	46	0
GO:0001701 in utero embryonic development	28	0
GO:0010629 negative regulation of gene expression	26	0
GO:0003151 outflow tract morphogenesis	15	0
GO:0043066 negative regulation of apoptotic process	34	0
GO:0045165 cell fate commitment	16	0
GO:0008284 positive regulation of cell proliferation	33	0
GO:0007507 heart development	24	0
GO:0006366 transcription from RNA polymerase II promoter	19	0
GO:1902895 positive regulation of pri-miRNA transcription from RNA polymerase II promoter	11	0
GO:0035914 skeletal muscle cell differentiation	14	0
GO:0010468 regulation of gene expression	25	0
GO:0030182 neuron differentiation	18	0
GO:0042127 regulation of cell proliferation	21	0
GO:0042493 response to drug	23	0
GO:0043401 steroid hormone mediated signaling pathway	12	0
GO:0007179 transforming growth factor beta receptor signaling pathway	13	0
GO:0001889 liver development	14	0
GO:0048663 neuron fate commitment	10	0
GO:0048511 rhythmic process	15	0
GO:0060021 palate development	13	0
GO:0021983 pituitary gland development	9	0
GO:0034097 response to cytokine	12	0
GO:0045666 positive regulation of neuron differentiation	13	0
GO:0048469 cell maturation	10	0
GO:0043388 positive regulation of DNA binding	9	0
GO:0007623 circadian rhythm	13	0
GO:0035116 embryonic hindlimb morphogenesis	9	0
GO:0010718 positive regulation of epithelial to mesenchymal transition	9	0
GO:0045669 positive regulation of osteoblast differentiation	11	0
GO:0060412 ventricular septum morphogenesis	9	0
GO:0045665 negative regulation of neuron differentiation	11	0

GO:0007219 Notch signaling pathway	13	0
GO:0007492 endoderm development	9	0
GO:0090090 negative regulation of canonical Wnt signaling pathway	12	0
GO:0030217 T cell differentiation	9	0
GO:0051216 cartilage development	11	0
GO:0042789 mRNA transcription from RNA polymerase II promoter	7	0
GO:0048596 embryonic camera-type eye morphogenesis	7	0
GO:0009887 organ morphogenesis	12	0
GO:0030097 hemopoiesis	11	0
GO:0030900 forebrain development	11	0
GO:0060216 definitive hemopoiesis	7	0
GO:0035019 somatic stem cell population maintenance	9	0
GO:0043525 positive regulation of neuron apoptotic process	10	0
GO:0050680 negative regulation of epithelial cell proliferation	10	0
GO:0030324 lung development	12	0
GO:0071407 cellular response to organic cyclic compound	10	0
GO:0035115 embryonic forelimb morphogenesis	8	0
GO:0030513 positive regulation of BMP signaling pathway	8	0
GO:0001822 kidney development	12	0
GO:0045444 fat cell differentiation	10	0
GO:0048701 embryonic cranial skeleton morphogenesis	8	0
GO:0003148 outflow tract septum morphogenesis	7	0
GO:0003198 epithelial to mesenchymal transition involved in endocardial cushion formation	6	0
GO:0001501 skeletal system development	11	0
GO:0002052 positive regulation of neuroblast proliferation	7	0
GO:0009952 anterior/posterior pattern specification	11	0
GO:0008584 male gonad development	11	0
GO:0009612 response to mechanical stimulus	9	0
GO:0030509 BMP signaling pathway	10	0
GO:0043065 positive regulation of apoptotic process	17	0
GO:0001892 embryonic placenta development	7	0
GO:0042733 embryonic digit morphogenesis	9	0
GO:0045599 negative regulation of fat cell differentiation	8	0
GO:0001657 ureteric bud development	8	0
GO:0001658 branching involved in ureteric bud morphogenesis	8	0
GO:0030218 erythrocyte differentiation	8	0
GO:0001503 ossification	10	0
GO:0001666 response to hypoxia	13	0
GO:0050767 regulation of neurogenesis	7	0
GO:0001837 epithelial to mesenchymal transition	7	0
GO:0042472 inner ear morphogenesis	9	0
GO:0007183 SMAD protein complex assembly	5	0
GO:0031016 pancreas development	7	0
GO:0017015 regulation of transforming growth factor beta receptor signaling pathway	6	0
GO:0060395 SMAD protein signal transduction	9	0
GO:0009749 response to glucose	9	0
GO:0051091 positive regulation of sequence-specific DNA binding transcription factor activity	10	0
GO:0007399 nervous system development	17	0
GO:0060548 negative regulation of cell death	9	0
GO:0007568 aging	12	0
GO:0032526 response to retinoic acid	8	0
GO:0030279 negative regulation of ossification	6	0
GO:0035162 embryonic hemopoiesis	6	0
GO:0048662 negative regulation of smooth muscle cell proliferation	7	0
GO:0030879 mammary gland development	7	0
GO:0001649 osteoblast differentiation	10	0
GO:0032332 positive regulation of chondrocyte differentiation	6	0

GO:0045595 regulation of cell differentiation	7	0
GO:0045787 positive regulation of cell cycle	7	0
GO:0001947 heart looping	8	0
GO:0031018 endocrine pancreas development	7	0
GO:0030326 embryonic limb morphogenesis	8	0
GO:0071158 positive regulation of cell cycle arrest	6	0
GO:0009880 embryonic pattern specification	6	0
GO:0030902 hindbrain development	6	0
GO:0048844 artery morphogenesis	6	0
GO:0006978 DNA damage response, signal transduction by p53 class mediator resulting in transcription of p21 class mediator	5	0
GO:0030308 negative regulation of cell growth	10	0
GO:0043524 negative regulation of neuron apoptotic process	11	0
GO:0009954 proximal/distal pattern formation	6	0
GO:0045662 negative regulation of myoblast differentiation	6	0
GO:0001568 blood vessel development	8	0
GO:0043966 histone H3 acetylation	7	0
GO:0002051 osteoblast fate commitment	4	0
GO:0051098 regulation of binding	4	0
GO:0051591 response to cAMP	7	0
GO:0006338 chromatin remodeling	8	0
GO:0043967 histone H4 acetylation	6	0
GO:0016569 covalent chromatin modification	13	0
GO:2000679 positive regulation of transcription regulatory region DNA binding	5	0
GO:0007050 cell cycle arrest	8	0
GO:0051726 regulation of cell cycle	9	0
GO:0043523 regulation of neuron apoptotic process	6	0
GO:0045596 negative regulation of cell differentiation	9	0
GO:0048863 stem cell differentiation	6	0
GO:0048485 sympathetic nervous system development	5	0
GO:0003203 endocardial cushion morphogenesis	5	0
GO:0070301 cellular response to hydrogen peroxide	7	0
GO:0007519 skeletal muscle tissue development	7	0
GO:0007389 pattern specification process	7	0
GO:0071773 cellular response to BMP stimulus	6	0
GO:0031069 hair follicle morphogenesis	6	0
GO:0048589 developmental growth	6	0
GO:0050728 negative regulation of inflammatory response	8	0.001
GO:0007417 central nervous system development	8	0.001
GO:0060070 canonical Wnt signaling pathway	8	0.001
GO:0007346 regulation of mitotic cell cycle	6	0.001
GO:0002053 positive regulation of mesenchymal cell proliferation	6	0.001
GO:0042981 regulation of apoptotic process	11	0.001
GO:2000020 positive regulation of male gonad development	4	0.001
GO:2000144 positive regulation of DNA-templated transcription, initiation	4	0.001
GO:0030219 megakaryocyte differentiation	4	0.001
GO:0001764 neuron migration	9	0.001
GO:0033077 T cell differentiation in thymus	6	0.001
GO:0071363 cellular response to growth factor stimulus	7	0.001
GO:0048568 embryonic organ development	6	0.001
GO:0071222 cellular response to lipopolysaccharide	11	0.001
GO:0030858 positive regulation of epithelial cell differentiation	4	0.001
GO:0036302 atrioventricular canal development	4	0.001
GO:0048565 digestive tract development	6	0.001
GO:0001570 vasculogenesis	7	0.001
GO:0043433 negative regulation of sequence-specific DNA binding transcription factor activity	7	0.001
GO:0060045 positive regulation of cardiac muscle cell proliferation	5	0.001
GO:0023019 signal transduction involved in regulation of gene expression	5	0.001
GO:0008283 cell proliferation	11	0.001

GO:0019827 stem cell population maintenance	7	0.001
GO:0060290 transdifferentiation	4	0.001
GO:0071375 cellular response to peptide hormone stimulus	5	0.002
GO:0045597 positive regulation of cell differentiation	6	0.002
GO:0002062 chondrocyte differentiation	6	0.002
GO:0097150 neuronal stem cell population maintenance	5	0.002
GO:0043627 response to estrogen	7	0.002
GO:0050679 positive regulation of epithelial cell proliferation	7	0.002
GO:0007498 mesoderm development	5	0.002
GO:0009953 dorsal/ventral pattern formation	6	0.002
GO:0030099 myeloid cell differentiation	5	0.002
GO:0030325 adrenal gland development	5	0.002
GO:0009791 post-embryonic development	8	0.002
GO:0021915 neural tube development	6	0.002
GO:0045603 positive regulation of endothelial cell differentiation	4	0.002
GO:0048667 cell morphogenesis involved in neuron differentiation	4	0.002
GO:0042752 regulation of circadian rhythm	6	0.003
GO:0060041 retina development in camera-type eye	7	0.003
GO:0051569 regulation of histone H3-K4 methylation	4	0.003
GO:0060391 positive regulation of SMAD protein import into nucleus	4	0.003
GO:0008015 blood circulation	4	0.003
GO:0048705 skeletal system morphogenesis	6	0.003
GO:0030216 keratinocyte differentiation	7	0.003
GO:0032570 response to progesterone	5	0.003
GO:0007569 cell aging	5	0.003
GO:0048704 embryonic skeletal system morphogenesis	6	0.003
GO:0048538 thymus development	6	0.003
GO:1901215 negative regulation of neuron death	6	0.004
GO:0042771 intrinsic apoptotic signaling pathway in response to DNA damage by p53 class mediator	5	0.004
GO:2000678 negative regulation of transcription regulatory region DNA binding	4	0.004
GO:0060411 cardiac septum morphogenesis	4	0.004
GO:0031065 positive regulation of histone deacetylation	4	0.004
GO:0043923 positive regulation by host of viral transcription	4	0.004
GO:0060349 bone morphogenesis	5	0.004
GO:0009611 response to wounding	6	0.004
GO:0010871 negative regulation of receptor biosynthetic process	3	0.004
GO:0003256 regulation of transcription from RNA polymerase II promoter involved in myocardial precursor cell differentiation	3	0.004
GO:0021986 habenula development	3	0.004
GO:0061138 morphogenesis of a branching epithelium	3	0.004
GO:0050821 protein stabilization	8	0.005
GO:0071345 cellular response to cytokine stimulus	5	0.005
GO:0071392 cellular response to estradiol stimulus	5	0.005
GO:0043586 tongue development	4	0.005
GO:0002064 epithelial cell development	4	0.005
GO:0010332 response to gamma radiation	5	0.005
GO:0048709 oligodendrocyte differentiation	5	0.005
GO:0071560 cellular response to transforming growth factor beta stimulus	6	0.005
GO:0032922 circadian regulation of gene expression	6	0.005
GO:0042060 wound healing	7	0.005
GO:0008544 epidermis development	6	0.005
GO:0016573 histone acetylation	5	0.006
GO:0061029 eyelid development in camera-type eye	4	0.006
GO:0035909 aorta morphogenesis	4	0.006
GO:0042475 odontogenesis of dentin-containing tooth	6	0.006
GO:0007517 muscle organ development	6	0.006
GO:0007369 gastrulation	5	0.006
GO:0003007 heart morphogenesis	6	0.006
GO:0010033 response to organic substance	6	0.007

GO:0071300 cellular response to retinoic acid	6	0.007
GO:0045747 positive regulation of Notch signaling pathway	5	0.007
GO:0055007 cardiac muscle cell differentiation	5	0.007
GO:0030501 positive regulation of bone mineralization	5	0.007
GO:0002089 lens morphogenesis in camera-type eye	4	0.007
GO:0003281 ventricular septum development	5	0.007
GO:0060364 frontal suture morphogenesis	3	0.008
GO:0060018 astrocyte fate commitment	3	0.008
GO:0021559 trigeminal nerve development	3	0.008
GO:0046533 negative regulation of photoreceptor cell differentiation	3	0.008
GO:0003266 regulation of secondary heart field cardioblast proliferation	3	0.008
GO:0030224 monocyte differentiation	4	0.008
GO:0060038 cardiac muscle cell proliferation	4	0.008
GO:0014902 myotube differentiation	4	0.008
GO:0003222 ventricular trabecula myocardium morphogenesis	4	0.008
GO:0035994 response to muscle stretch	4	0.008
GO:0050678 regulation of epithelial cell proliferation	4	0.008
GO:0006915 apoptotic process	16	0.008
GO:0030901 midbrain development	5	0.009
GO:0006954 inflammatory response	12	0.009
GO:0002076 osteoblast development	4	0.009
GO:0001709 cell fate determination	4	0.009
GO:0060037 pharyngeal system development	4	0.009
GO:0045598 regulation of fat cell differentiation	4	0.009
GO:0032496 response to lipopolysaccharide	9	0.011
GO:0060065 uterus development	4	0.011
GO:0030220 platelet formation	4	0.011
GO:0048706 embryonic skeletal system development	5	0.012
GO:0070345 negative regulation of fat cell proliferation	3	0.012
GO:0003211 cardiac ventricle formation	3	0.012
GO:0010742 macrophage derived foam cell differentiation	3	0.012
GO:0001714 endodermal cell fate specification	3	0.012
GO:0061419 positive regulation of transcription from RNA polymerase II promoter in response to hypoxia	3	0.012
GO:0038061 NIK/NF-kappaB signaling	3	0.012
GO:0030539 male genitalia development	4	0.012
GO:0071260 cellular response to mechanical stimulus	6	0.014
GO:0034644 cellular response to UV	5	0.014
GO:0045648 positive regulation of erythrocyte differentiation	4	0.014
GO:0071347 cellular response to interleukin-1	6	0.015
GO:0043011 myeloid dendritic cell differentiation	4	0.016
GO:0031668 cellular response to extracellular stimulus	4	0.016
GO:0010942 positive regulation of cell death	5	0.016
GO:0001755 neural crest cell migration	5	0.018
GO:1990314 cellular response to insulin-like growth factor stimulus	3	0.018
GO:0065004 protein-DNA complex assembly	3	0.018
GO:0072602 interleukin-4 secretion	3	0.018
GO:0014842 regulation of skeletal muscle satellite cell proliferation	3	0.018
GO:0048505 regulation of timing of cell differentiation	3	0.018
GO:0043353 enucleate erythrocyte differentiation	3	0.018
GO:0001708 cell fate specification	4	0.018
GO:0035050 embryonic heart tube development	4	0.018
GO:0090190 positive regulation of branching involved in ureteric bud morphogenesis	4	0.018
GO:0032024 positive regulation of insulin secretion	5	0.019
GO:0048646 anatomical structure formation involved in morphogenesis	4	0.02
GO:0042474 middle ear morphogenesis	4	0.02
GO:0009987 cellular process	4	0.02
GO:0071277 cellular response to calcium ion	5	0.023
GO:0010944 negative regulation of transcription by competitive promoter binding	3	0.024

GO:0071599 otic vesicle development	3	0.024
GO:0042421 norepinephrine biosynthetic process	3	0.024
GO:0034616 response to laminar fluid shear stress	3	0.024
GO:0009913 epidermal cell differentiation	3	0.024
GO:0003180 aortic valve morphogenesis	3	0.024
GO:0060261 positive regulation of transcription initiation from RNA polymerase II promoter	3	0.024
GO:0032808 lacrimal gland development	3	0.024
GO:1901522 positive regulation of transcription from RNA polymerase II promoter involved in cellular response to chemical stimulus	3	0.024
GO:0009629 response to gravity	3	0.024
GO:0060536 cartilage morphogenesis	3	0.024
GO:0071316 cellular response to nicotine	3	0.024
GO:0002467 germinal center formation	3	0.024
GO:0031100 organ regeneration	5	0.028
GO:0014807 regulation of somitogenesis	3	0.031
GO:0003139 secondary heart field specification	3	0.031
GO:0045084 positive regulation of interleukin-12 biosynthetic process	3	0.031
GO:0060136 embryonic process involved in female pregnancy	3	0.031
GO:0045930 negative regulation of mitotic cell cycle	4	0.034
GO:0097421 liver regeneration	4	0.034
GO:0071542 dopaminergic neuron differentiation	4	0.034
GO:0009267 cellular response to starvation	5	0.035
GO:0042517 positive regulation of tyrosine phosphorylation of Stat3 protein	4	0.037
GO:0003309 type B pancreatic cell differentiation	3	0.039
GO:0061549 sympathetic ganglion development	3	0.039
GO:0021984 adenohypophysis development	3	0.039
GO:0032355 response to estradiol	6	0.039
GO:0071456 cellular response to hypoxia	6	0.042
GO:0000060 protein import into nucleus, translocation	4	0.044
GO:0007249 I-kappaB kinase/NF-kappaB signaling	4	0.044
GO:0022008 neurogenesis	5	0.045
GO:0048146 positive regulation of fibroblast proliferation	5	0.045
GO:0048730 epidermis morphogenesis	3	0.047
GO:0030851 granulocyte differentiation	3	0.047
GO:0070166 enamel mineralization	3	0.047
GO:0042118 endothelial cell activation	3	0.047
GO:0048839 inner ear development	5	0.047
GO:0006461 protein complex assembly	5	0.047
GO:0030335 positive regulation of cell migration	8	0.048
mmu05202:Transcriptional misregulation in cancer	27	0
mmu05166:HTLV-I infection	32	0
mmu05161:Hepatitis B	24	0
mmu05200:Pathways in cancer	31	0
mmu04350:TGF-beta signaling pathway	15	0
mmu05203:Viral carcinogenesis	21	0
mmu05212:Pancreatic cancer	12	0
mmu05215:Prostate cancer	13	0
mmu05220:Chronic myeloid leukemia	12	0
mmu04550:Signaling pathways regulating pluripotency of stem cells	15	0
mmu04110:Cell cycle	14	0
mmu04668:TNF signaling pathway	12	0
mmu04380:Osteoclast differentiation	12	0
mmu05321:Inflammatory bowel disease (IBD)	9	0
mmu05210:Colorectal cancer	9	0
mmu04919:Thyroid hormone signaling pathway	11	0
mmu05221:Acute myeloid leukemia	8	0
mmu05169:Epstein-Barr virus infection	11	0
mmu05222:Small cell lung cancer	9	0
mmu05030:Cocaine addiction	7	0

mmu04917:Prolactin signaling pathway	8	0
mmu04915:Estrogen signaling pathway	9	0
mmu04024:cAMP signaling pathway	12	0.001
mmu04068:FoxO signaling pathway	10	0.001
mmu04310:Wnt signaling pathway	10	0.001
mmu04390:Hippo signaling pathway	10	0.001
mmu05219:Bladder cancer	6	0.001
mmu04520:Adherens junction	7	0.002
mmu05206:MicroRNAs in cancer	13	0.002
mmu04922:Glucagon signaling pathway	8	0.002
mmu04330:Notch signaling pathway	6	0.002
mmu05133:Pertussis	7	0.002
mmu05162:Measles	9	0.002
mmu04010:MAPK signaling pathway	12	0.003
mmu04931:Insulin resistance	8	0.003
mmu05140:Leishmaniasis	6	0.007
mmu05031:Amphetamine addiction	6	0.008
mmu05211:Renal cell carcinoma	6	0.008
mmu05164:Influenza A	9	0.009
mmu05160:Hepatitis C	8	0.009
mmu05152:Tuberculosis	9	0.01
mmu05142:Chagas disease (American trypanosomiasis)	7	0.01
mmu04320:Dorso-ventral axis formation	4	0.014
mmu04950:Maturity onset diabetes of the young	4	0.018
mmu05016:Huntington's disease	9	0.018
mmu05223:Non-small cell lung cancer	5	0.02
mmu05205:Proteoglycans in cancer	9	0.02
mmu05216:Thyroid cancer	4	0.021
mmu05168:Herpes simplex infection	9	0.022
mmu04620:Toll-like receptor signaling pathway	6	0.034
mmu04630:Jak-STAT signaling pathway	7	0.039
mmu05145:Toxoplasmosis	6	0.039
mmu04662:B cell receptor signaling pathway	5	0.039

Table S25: Enriched GO terms and KEGG pathways with adjusted p-values < 0.05 for the TFs involved in the B-cell fate process.

Enriched terms	count	adj. p-values
GO:0006351 transcription, DNA-templated	155	0
GO:0006355 regulation of transcription, DNA-templated	159	0
GO:0045944 positive regulation of transcription from RNA polymerase II promoter	127	0
GO:0045893 positive regulation of transcription, DNA-templated	100	0
GO:0000122 negative regulation of transcription from RNA polymerase II promoter	93	0
GO:0045892 negative regulation of transcription, DNA-templated	57	0
GO:0006357 regulation of transcription from RNA polymerase II promoter	44	0
GO:0010628 positive regulation of gene expression	39	0
GO:0008285 negative regulation of cell proliferation	32	0
GO:0030154 cell differentiation	42	0
GO:1902895 positive regulation of pri-miRNA transcription from RNA polymerase II promoter	12	0
GO:0010468 regulation of gene expression	26	0
GO:0007275 multicellular organism development	43	0
GO:0007507 heart development	24	0
GO:0006366 transcription from RNA polymerase II promoter	19	0
GO:0043066 negative regulation of apoptotic process	32	0
GO:0042127 regulation of cell proliferation	22	0
GO:0030182 neuron differentiation	18	0
GO:0008284 positive regulation of cell proliferation	31	0
GO:0043401 steroid hormone mediated signaling pathway	13	0

GO:0048511 rhythmic process	17	0
GO:0010629 negative regulation of gene expression	22	0
GO:0003151 outflow tract morphogenesis	13	0
GO:0001701 in utero embryonic development	23	0
GO:0042493 response to drug	24	0
GO:0007179 transforming growth factor beta receptor signaling pathway	14	0
GO:0034097 response to cytokine	14	0
GO:0045165 cell fate commitment	13	0
GO:0001889 liver development	14	0
GO:0007623 circadian rhythm	14	0
GO:0001666 response to hypoxia	17	0
GO:0035914 skeletal muscle cell differentiation	11	0
GO:0060412 ventricular septum morphogenesis	9	0
GO:0045666 positive regulation of neuron differentiation	12	0
GO:0030217 T cell differentiation	9	0
GO:0001892 embryonic placenta development	8	0
GO:0048596 embryonic camera-type eye morphogenesis	7	0
GO:0048469 cell maturation	9	0
GO:0035019 somatic stem cell population maintenance	9	0
GO:0060216 definitive hemopoiesis	7	0
GO:0045669 positive regulation of osteoblast differentiation	10	0
GO:0030218 erythrocyte differentiation	9	0
GO:0048663 neuron fate commitment	8	0
GO:0010718 positive regulation of epithelial to mesenchymal transition	8	0
GO:0007219 Notch signaling pathway	12	0
GO:0035162 embryonic hemopoiesis	7	0
GO:0060395 SMAD protein signal transduction	10	0
GO:0009749 response to glucose	10	0
GO:0001501 skeletal system development	11	0
GO:0007492 endoderm development	8	0
GO:0007568 aging	13	0
GO:0060021 palate development	10	0
GO:0030097 hemopoiesis	10	0
GO:0009612 response to mechanical stimulus	9	0
GO:0030219 megakaryocyte differentiation	5	0
GO:2000144 positive regulation of DNA-templated transcription, initiation	5	0
GO:0043525 positive regulation of neuron apoptotic process	9	0
GO:0021983 pituitary gland development	7	0
GO:0001657 ureteric bud development	8	0
GO:0045599 negative regulation of fat cell differentiation	8	0
GO:0050680 negative regulation of epithelial cell proliferation	9	0
GO:0048485 sympathetic nervous system development	6	0
GO:0001822 kidney development	11	0
GO:0043388 positive regulation of DNA binding	7	0
GO:0007183 SMAD protein complex assembly	5	0
GO:0071407 cellular response to organic cyclic compound	9	0
GO:0090090 negative regulation of canonical Wnt signaling pathway	10	0
GO:0017015 regulation of transforming growth factor beta receptor signaling pathway	6	0
GO:0009887 organ morphogenesis	10	0
GO:0051216 cartilage development	9	0
GO:0030279 negative regulation of ossification	6	0
GO:0048701 embryonic cranial skeleton morphogenesis	7	0
GO:0071560 cellular response to transforming growth factor beta stimulus	8	0
GO:0032332 positive regulation of chondrocyte differentiation	6	0
GO:0030509 BMP signaling pathway	9	0
GO:0001947 heart looping	8	0
GO:0031018 endocrine pancreas development	7	0
GO:0030902 hindbrain development	6	0
GO:0009880 embryonic pattern specification	6	0

GO:0030324 lung development	10	0
GO:0043524 negative regulation of neuron apoptotic process	11	0
GO:0030308 negative regulation of cell growth	10	0
GO:0002052 positive regulation of neuroblast proliferation	6	0
GO:0043433 negative regulation of sequence-specific DNA binding transcription factor activity	8	0
GO:0043065 positive regulation of apoptotic process	15	0
GO:0001568 blood vessel development	8	0
GO:0001658 branching involved in ureteric bud morphogenesis	7	0
GO:0051098 regulation of binding	4	0
GO:0038061 NIK/NF-kappaB signaling	4	0
GO:0051591 response to cAMP	7	0
GO:0008283 cell proliferation	12	0
GO:0016569 covalent chromatin modification	13	0
GO:0001837 epithelial to mesenchymal transition	6	0
GO:0006338 chromatin remodeling	8	0
GO:0009952 anterior/posterior pattern specification	9	0
GO:0051726 regulation of cell cycle	9	0
GO:0007249 I-kappaB kinase/NF-kappaB signaling	6	0
GO:0007050 cell cycle arrest	8	0
GO:0001649 osteoblast differentiation	9	0
GO:0035116 embryonic hindlimb morphogenesis	6	0
GO:0043523 regulation of neuron apoptotic process	6	0
GO:0032526 response to retinoic acid	7	0
GO:0042789 mRNA transcription from RNA polymerase II promoter	5	0
GO:0007389 pattern specification process	7	0
GO:0007519 skeletal muscle tissue development	7	0
GO:0048589 developmental growth	6	0
GO:0071773 cellular response to BMP stimulus	6	0
GO:0042981 regulation of apoptotic process	11	0
GO:0035115 embryonic forelimb morphogenesis	6	0
GO:0007417 central nervous system development	8	0
GO:0032922 circadian regulation of gene expression	7	0.001
GO:0030220 platelet formation	5	0.001
GO:0002467 germinal center formation	4	0.001
GO:0030879 mammary gland development	6	0.001
GO:0045595 regulation of cell differentiation	6	0.001
GO:0001503 ossification	8	0.001
GO:0045787 positive regulation of cell cycle	6	0.001
GO:0042733 embryonic digit morphogenesis	7	0.001
GO:0045648 positive regulation of erythrocyte differentiation	5	0.001
GO:0036302 atrioventricular canal development	4	0.001
GO:0030858 positive regulation of epithelial cell differentiation	4	0.001
GO:0048565 digestive tract development	6	0.001
GO:0032355 response to estradiol	8	0.001
GO:0071456 cellular response to hypoxia	8	0.001
GO:0007399 nervous system development	14	0.001
GO:0002062 chondrocyte differentiation	6	0.001
GO:0051091 positive regulation of sequence-specific DNA binding transcription factor activity	8	0.001
GO:0043627 response to estrogen	7	0.002
GO:0007265 Ras protein signal transduction	6	0.002
GO:0043966 histone H3 acetylation	6	0.002
GO:0050679 positive regulation of epithelial cell proliferation	7	0.002
GO:0045665 negative regulation of neuron differentiation	7	0.002
GO:0048844 artery morphogenesis	5	0.002
GO:0030851 granulocyte differentiation	4	0.002
GO:0008584 male gonad development	8	0.002
GO:0009954 proximal/distal pattern formation	5	0.002
GO:0045596 negative regulation of cell differentiation	8	0.002

GO:0071347 cellular response to interleukin-1	7	0.002
GO:0042752 regulation of circadian rhythm	6	0.002
GO:0071542 dopaminergic neuron differentiation	5	0.003
GO:0045930 negative regulation of mitotic cell cycle	5	0.003
GO:0045722 positive regulation of gluconeogenesis	4	0.003
GO:0060391 positive regulation of SMAD protein import into nucleus	4	0.003
GO:0006978 DNA damage response, signal transduction by p53 class mediator resulting in transcription of p21 class mediator	4	0.003
GO:0008015 blood circulation	4	0.003
GO:0007569 cell aging	5	0.003
GO:0032570 response to progesterone	5	0.003
GO:0050728 negative regulation of inflammatory response	7	0.003
GO:0001764 neuron migration	8	0.003
GO:1901215 negative regulation of neuron death	6	0.003
GO:0042771 intrinsic apoptotic signaling pathway in response to DNA damage by p53 class mediator	5	0.003
GO:0050767 regulation of neurogenesis	5	0.003
GO:0043923 positive regulation by host of viral transcription	4	0.004
GO:0031065 positive regulation of histone deacetylation	4	0.004
GO:0043967 histone H4 acetylation	5	0.004
GO:0000060 protein import into nucleus, translocation	5	0.004
GO:0031016 pancreas development	5	0.004
GO:0010871 negative regulation of receptor biosynthetic process	3	0.004
GO:0002064 epithelial cell development	4	0.005
GO:0043586 tongue development	4	0.005
GO:0042593 glucose homeostasis	8	0.005
GO:0010332 response to gamma radiation	5	0.005
GO:0042475 odontogenesis of dentin-containing tooth	6	0.005
GO:0031069 hair follicle morphogenesis	5	0.005
GO:0061029 eyelid development in camera-type eye	4	0.006
GO:0035909 aorta morphogenesis	4	0.006
GO:0007369 gastrulation	5	0.006
GO:0030513 positive regulation of BMP signaling pathway	5	0.006
GO:0048146 positive regulation of fibroblast proliferation	6	0.006
GO:0055007 cardiac muscle cell differentiation	5	0.006
GO:0030501 positive regulation of bone mineralization	5	0.006
GO:0002053 positive regulation of mesenchymal cell proliferation	5	0.006
GO:2000679 positive regulation of transcription regulatory region DNA binding	4	0.007
GO:0035729 cellular response to hepatocyte growth factor stimulus	4	0.007
GO:0002089 lens morphogenesis in camera-type eye	4	0.007
GO:0048662 negative regulation of smooth muscle cell proliferation	5	0.007
GO:0006954 inflammatory response	12	0.007
GO:0001570 vasculogenesis	6	0.007
GO:0033077 T cell differentiation in thymus	5	0.007
GO:0045670 regulation of osteoclast differentiation	4	0.008
GO:0030224 monocyte differentiation	4	0.008
GO:0035994 response to muscle stretch	4	0.008
GO:0032909 regulation of transforming growth factor beta2 production	3	0.008
GO:0019827 stem cell population maintenance	6	0.008
GO:0032496 response to lipopolysaccharide	9	0.009
GO:0042472 inner ear morphogenesis	6	0.009
GO:0001709 cell fate determination	4	0.009
GO:0045598 regulation of fat cell differentiation	4	0.009
GO:0035855 megakaryocyte development	4	0.01
GO:0060065 uterus development	4	0.01
GO:0030198 extracellular matrix organization	7	0.011
GO:0030539 male genitalia development	4	0.012
GO:0030318 melanocyte differentiation	4	0.012
GO:0071222 cellular response to lipopolysaccharide	9	0.012
GO:0071260 cellular response to mechanical stimulus	6	0.012

GO:0001714 endodermal cell fate specification	3	0.012
GO:0061419 positive regulation of transcription from RNA polymerase II promoter in response to hypoxia	3	0.012
GO:0002051 osteoblast fate commitment	3	0.012
GO:0070345 negative regulation of fat cell proliferation	3	0.012
GO:0003211 cardiac ventricle formation	3	0.012
GO:0010424 DNA methylation on cytosine within a CG sequence	3	0.012
GO:0010742 macrophage derived foam cell differentiation	3	0.012
GO:0034644 cellular response to UV	5	0.013
GO:0045597 positive regulation of cell differentiation	5	0.013
GO:0048661 positive regulation of smooth muscle cell proliferation	6	0.013
GO:0060548 negative regulation of cell death	6	0.013
GO:0001829 trophectodermal cell differentiation	4	0.013
GO:0050796 regulation of insulin secretion	5	0.014
GO:0045766 positive regulation of angiogenesis	7	0.014
GO:0010942 positive regulation of cell death	5	0.015
GO:0043011 myeloid dendritic cell differentiation	4	0.015
GO:0031668 cellular response to extracellular stimulus	4	0.015
GO:0023019 signal transduction involved in regulation of gene expression	4	0.017
GO:0035050 embryonic heart tube development	4	0.017
GO:0042634 regulation of hair cycle	3	0.017
GO:0044336 canonical Wnt signaling pathway involved in negative regulation of apoptotic process	3	0.017
GO:0065004 protein-DNA complex assembly	3	0.017
GO:0048505 regulation of timing of cell differentiation	3	0.017
GO:1990314 cellular response to insulin-like growth factor stimulus	3	0.017
GO:0043353 enucleate erythrocyte differentiation	3	0.017
GO:0030900 forebrain development	6	0.018
GO:0021915 neural tube development	5	0.018
GO:0050821 protein stabilization	7	0.018
GO:0009987 cellular process	4	0.019
GO:0048646 anatomical structure formation involved in morphogenesis	4	0.019
GO:0003148 outflow tract septum morphogenesis	4	0.019
GO:0032869 cellular response to insulin stimulus	6	0.019
GO:0060070 canonical Wnt signaling pathway	6	0.02
GO:0048705 skeletal system morphogenesis	5	0.021
GO:0032808 lacrimal gland development	3	0.022
GO:0034616 response to laminar fluid shear stress	3	0.022
GO:1901522 positive regulation of transcription from RNA polymerase II promoter involved in cellular response to chemical stimulus	3	0.022
GO:0060261 positive regulation of transcription initiation from RNA polymerase II promoter	3	0.022
GO:0003180 aortic valve morphogenesis	3	0.022
GO:0071599 otic vesicle development	3	0.022
GO:2000020 positive regulation of male gonad development	3	0.022
GO:0003179 heart valve morphogenesis	3	0.022
GO:0010944 negative regulation of transcription by competitive promoter binding	3	0.022
GO:0007283 spermatogenesis	12	0.022
GO:0048538 thymus development	5	0.023
GO:0048704 embryonic skeletal system morphogenesis	5	0.023
GO:0001944 vasculature development	4	0.023
GO:0045662 negative regulation of myoblast differentiation	4	0.023
GO:0007498 mesoderm development	4	0.023
GO:0045668 negative regulation of osteoblast differentiation	5	0.024
GO:0031100 organ regeneration	5	0.024
GO:0042060 wound healing	6	0.024
GO:0030099 myeloid cell differentiation	4	0.025
GO:0030325 adrenal gland development	4	0.025
GO:0070301 cellular response to hydrogen peroxide	5	0.025
GO:0009611 response to wounding	5	0.027

GO:0007406 negative regulation of neuroblast proliferation	3	0.028
GO:0010216 maintenance of DNA methylation	3	0.028
GO:0060136 embryonic process involved in female pregnancy	3	0.028
GO:0045084 positive regulation of interleukin-12 biosynthetic process	3	0.028
GO:0097421 liver regeneration	4	0.03
GO:0032091 negative regulation of protein binding	5	0.031
GO:0060290 transdifferentiation	3	0.036
GO:0045664 regulation of neuron differentiation	4	0.036
GO:1990090 cellular response to nerve growth factor stimulus	4	0.036
GO:0006915 apoptotic process	14	0.036
GO:0048147 negative regulation of fibroblast proliferation	4	0.039
GO:0010033 response to organic substance	5	0.039
GO:0006461 protein complex assembly	5	0.04
GO:0048839 inner ear development	5	0.04
GO:0019216 regulation of lipid metabolic process	4	0.042
GO:0071345 cellular response to cytokine stimulus	4	0.042
GO:0006346 methylation-dependent chromatin silencing	3	0.043
GO:0042118 endothelial cell activation	3	0.043
GO:0071356 cellular response to tumor necrosis factor	6	0.044
GO:0048863 stem cell differentiation	4	0.045
GO:0000165 MAPK cascade	5	0.046
GO:0016573 histone acetylation	4	0.048
GO:0001707 mesoderm formation	4	0.048
GO:0009617 response to bacterium	4	0.048
mmu05202:Transcriptional misregulation in cancer	25	0
mmu05200:Pathways in cancer	34	0
mmu05166:HTLV-I infection	29	0
mmu05161:Hepatitis B	23	0
mmu04350:TGF-beta signaling pathway	17	0
mmu04380:Osteoclast differentiation	17	0
mmu05212:Pancreatic cancer	13	0
mmu05215:Prostate cancer	13	0
mmu05220:Chronic myeloid leukemia	12	0
mmu05321:Inflammatory bowel disease (IBD)	11	0
mmu04110:Cell cycle	14	0
mmu05203:Viral carcinogenesis	18	0
mmu04550:Signaling pathways regulating pluripotency of stem cells	14	0
mmu05210:Colorectal cancer	9	0
mmu05169:Epstein-Barr virus infection	12	0
mmu04919:Thyroid hormone signaling pathway	11	0
mmu05221:Acute myeloid leukemia	8	0
mmu05222:Small cell lung cancer	9	0
mmu04668:TNF signaling pathway	10	0
mmu04024:cAMP signaling pathway	13	0
mmu04310:Wnt signaling pathway	11	0
mmu05152:Tuberculosis	12	0
mmu05211:Renal cell carcinoma	8	0
mmu04390:Hippo signaling pathway	11	0
mmu04068:FoxO signaling pathway	10	0.001
mmu05206:MicroRNAs in cancer	14	0.001
mmu04931:Insulin resistance	9	0.001
mmu05168:Herpes simplex infection	12	0.001
mmu04010:MAPK signaling pathway	13	0.001
mmu05219:Bladder cancer	6	0.001
mmu04662:B cell receptor signaling pathway	7	0.002
mmu04950:Maturity onset diabetes of the young	5	0.002
mmu04520:Adherens junction	7	0.002
mmu04917:Prolactin signaling pathway	7	0.002
mmu05142:Chagas disease (American trypanosomiasis)	8	0.003
mmu05133:Pertussis	7	0.003

mmu05205:Proteoglycans in cancer	11	0.003
mmu05160:Hepatitis C	9	0.003
mmu05140:Leishmaniasis	6	0.008
mmu04915:Estrogen signaling pathway	7	0.01
mmu04620:Toll-like receptor signaling pathway	7	0.011
mmu04660:T cell receptor signaling pathway	7	0.011
mmu05164:Influenza A	9	0.011
mmu05218:Melanoma	6	0.011
mmu05145:Toxoplasmosis	7	0.012
mmu04330:Notch signaling pathway	5	0.016
mmu05030:Cocaine addiction	5	0.016
mmu05016:Huntington's disease	9	0.023
mmu05216:Thyroid cancer	4	0.023
mmu05223:Non-small cell lung cancer	5	0.023
mmu04710:Circadian rhythm	4	0.026
mmu05340:Primary immunodeficiency	4	0.033
mmu05162:Measles	7	0.036
mmu04916:Melanogenesis	6	0.036
mmu04922:Glucagon signaling pathway	6	0.036
mmu04066:HIF-1 signaling pathway	6	0.039
mmu04630:Jak-STAT signaling pathway	7	0.044
mmu04920:Adipocytokine signaling pathway	5	0.047

Table S26: Enriched GO terms and KEGG pathways with adjusted p-values < 0.05 for the TFs involved in the erythrocyte cell fate process.

Enriched terms	count	adj. p-values
GO:0045944 positive regulation of transcription from RNA polymerase II promoter	91	0
GO:0006351 transcription, DNA-templated	105	0
GO:0006355 regulation of transcription, DNA-templated	109	0
GO:0045893 positive regulation of transcription, DNA-templated	63	0
GO:0000122 negative regulation of transcription from RNA polymerase II promoter	66	0
GO:0006357 regulation of transcription from RNA polymerase II promoter	40	0
GO:0045892 negative regulation of transcription, DNA-templated	42	0
GO:0010628 positive regulation of gene expression	31	0
GO:0043066 negative regulation of apoptotic process	30	0
GO:0043065 positive regulation of apoptotic process	23	0
GO:0006366 transcription from RNA polymerase II promoter	16	0
GO:0034097 response to cytokine	13	0
GO:0042127 regulation of cell proliferation	18	0
GO:1902895 positive regulation of pri-miRNA transcription from RNA polymerase II promoter	9	0
GO:0008284 positive regulation of cell proliferation	24	0
GO:0001701 in utero embryonic development	18	0
GO:0007507 heart development	17	0
GO:0010629 negative regulation of gene expression	17	0
GO:0010468 regulation of gene expression	18	0
GO:0060548 negative regulation of cell death	11	0
GO:0070301 cellular response to hydrogen peroxide	10	0
GO:0048511 rhythmic process	12	0
GO:0008285 negative regulation of cell proliferation	18	0
GO:0043401 steroid hormone mediated signaling pathway	9	0
GO:0042493 response to drug	16	0
GO:0007275 multicellular organism development	26	0
GO:0045165 cell fate commitment	9	0
GO:2000378 negative regulation of reactive oxygen species metabolic process	7	0
GO:0071407 cellular response to organic cyclic compound	9	0
GO:0007179 transforming growth factor beta receptor signaling pathway	9	0
GO:0050767 regulation of neurogenesis	7	0

GO:0007050 cell cycle arrest	9	0
GO:0030154 cell differentiation	22	0
GO:0035914 skeletal muscle cell differentiation	8	0
GO:0010718 positive regulation of epithelial to mesenchymal transition	7	0
GO:0006915 apoptotic process	18	0
GO:0043525 positive regulation of neuron apoptotic process	8	0
GO:0009987 cellular process	6	0
GO:0071456 cellular response to hypoxia	9	0
GO:0001658 branching involved in ureteric bud morphogenesis	7	0
GO:0045722 positive regulation of gluconeogenesis	5	0
GO:0006978 DNA damage response, signal transduction by p53 class mediator resulting in transcription of p21 class mediator	5	0
GO:0071347 cellular response to interleukin-1	8	0
GO:0051216 cartilage development	8	0
GO:0030900 forebrain development	8	0
GO:1901215 negative regulation of neuron death	7	0
GO:0030308 negative regulation of cell growth	9	0
GO:0002051 osteoblast fate commitment	4	0
GO:0038061 NIK/NF-kappaB signaling	4	0
GO:0001889 liver development	8	0
GO:0003203 endocardial cushion morphogenesis	5	0
GO:0060412 ventricular septum morphogenesis	6	0
GO:0048662 negative regulation of smooth muscle cell proliferation	6	0
GO:0032355 response to estradiol	8	0
GO:0045669 positive regulation of osteoblast differentiation	7	0
GO:0030217 T cell differentiation	6	0
GO:0001501 skeletal system development	8	0
GO:0051726 regulation of cell cycle	8	0.001
GO:0048706 embryonic skeletal system development	6	0.001
GO:0032332 positive regulation of chondrocyte differentiation	5	0.001
GO:0050679 positive regulation of epithelial cell proliferation	7	0.001
GO:0035019 somatic stem cell population maintenance	6	0.001
GO:0034644 cellular response to UV	6	0.001
GO:0030856 regulation of epithelial cell differentiation	4	0.001
GO:0030858 positive regulation of epithelial cell differentiation	4	0.001
GO:0001708 cell fate specification	5	0.001
GO:0006338 chromatin remodeling	7	0.001
GO:0001657 ureteric bud development	6	0.001
GO:0045599 negative regulation of fat cell differentiation	6	0.001
GO:0071375 cellular response to peptide hormone stimulus	5	0.001
GO:0001764 neuron migration	8	0.001
GO:0030324 lung development	8	0.001
GO:0060070 canonical Wnt signaling pathway	7	0.001
GO:0003170 heart valve development	4	0.001
GO:0042593 glucose homeostasis	8	0.001
GO:0001892 embryonic placenta development	5	0.001
GO:0048708 astrocyte differentiation	4	0.002
GO:0001666 response to hypoxia	9	0.002
GO:0042771 intrinsic apoptotic signaling pathway in response to DNA damage by p53 class mediator	5	0.002
GO:0048663 neuron fate commitment	5	0.002
GO:1902894 negative regulation of pri-miRNA transcription from RNA polymerase II promoter	4	0.002
GO:0090090 negative regulation of canonical Wnt signaling pathway	7	0.002
GO:0007517 muscle organ development	6	0.002
GO:0009612 response to mechanical stimulus	6	0.002
GO:0035116 embryonic hindlimb morphogenesis	5	0.002
GO:0043523 regulation of neuron apoptotic process	5	0.002
GO:0043518 negative regulation of DNA damage response, signal transduction by p53 class mediator	4	0.002

GO:0010832 negative regulation of myotube differentiation	4	0.002
GO:0043923 positive regulation by host of viral transcription	4	0.002
GO:0006461 protein complex assembly	6	0.003
GO:0016573 histone acetylation	5	0.003
GO:0051091 positive regulation of sequence-specific DNA binding transcription factor activity	7	0.003
GO:0035115 embryonic forelimb morphogenesis	5	0.003
GO:0071222 cellular response to lipopolysaccharide	9	0.003
GO:0001570 vasculogenesis	6	0.003
GO:0009887 organ morphogenesis	7	0.003
GO:0043433 negative regulation of sequence-specific DNA binding transcription factor activity	6	0.003
GO:0007346 regulation of mitotic cell cycle	5	0.003
GO:0002053 positive regulation of mesenchymal cell proliferation	5	0.003
GO:0009952 anterior/posterior pattern specification	7	0.003
GO:0032868 response to insulin	6	0.003
GO:0001568 blood vessel development	6	0.003
GO:0045667 regulation of osteoblast differentiation	4	0.003
GO:0043124 negative regulation of I-kappaB kinase/NF-kappaB signaling	5	0.004
GO:0008283 cell proliferation	9	0.004
GO:0006974 cellular response to DNA damage stimulus	12	0.004
GO:0060395 SMAD protein signal transduction	6	0.004
GO:0007568 aging	8	0.005
GO:0048485 sympathetic nervous system development	4	0.005
GO:0060038 cardiac muscle cell proliferation	4	0.005
GO:0045670 regulation of osteoclast differentiation	4	0.005
GO:0035994 response to muscle stretch	4	0.005
GO:0048596 embryonic camera-type eye morphogenesis	4	0.005
GO:0045444 fat cell differentiation	6	0.005
GO:0030182 neuron differentiation	7	0.006
GO:0048469 cell maturation	5	0.006
GO:0050821 protein stabilization	7	0.006
GO:0032909 regulation of transforming growth factor beta2 production	3	0.006
GO:1900212 negative regulation of mesenchymal cell apoptotic process involved in metanephros development	3	0.006
GO:0072289 metanephric nephron tubule formation	3	0.006
GO:0060065 uterus development	4	0.006
GO:0030514 negative regulation of BMP signaling pathway	5	0.006
GO:0001822 kidney development	7	0.006
GO:0030509 BMP signaling pathway	6	0.007
GO:0050728 negative regulation of inflammatory response	6	0.007
GO:0032869 cellular response to insulin stimulus	6	0.007
GO:0045600 positive regulation of fat cell differentiation	5	0.007
GO:0051591 response to cAMP	5	0.008
GO:0045736 negative regulation of cyclin-dependent protein serine/threonine kinase activity	4	0.009
GO:0061419 positive regulation of transcription from RNA polymerase II promoter in response to hypoxia	3	0.009
GO:0010742 macrophage derived foam cell differentiation	3	0.009
GO:0001922 B-1 B cell homeostasis	3	0.009
GO:0008630 intrinsic apoptotic signaling pathway in response to DNA damage	5	0.01
GO:0003151 outflow tract morphogenesis	5	0.01
GO:0071158 positive regulation of cell cycle arrest	4	0.011
GO:0030902 hindbrain development	4	0.011
GO:0009880 embryonic pattern specification	4	0.011
GO:0001541 ovarian follicle development	5	0.012
GO:0044336 canonical Wnt signaling pathway involved in negative regulation of apoptotic process	3	0.013
GO:1901985 positive regulation of protein acetylation	3	0.013
GO:0009954 proximal/distal pattern formation	4	0.014

GO:0045662 negative regulation of myoblast differentiation	4	0.014
GO:0002052 positive regulation of neuroblast proliferation	4	0.014
GO:0030878 thyroid gland development	4	0.014
GO:1901216 positive regulation of neuron death	4	0.015
GO:0071560 cellular response to transforming growth factor beta stimulus	5	0.015
GO:0043524 negative regulation of neuron apoptotic process	7	0.016
GO:0008544 epidermis development	5	0.016
GO:0007623 circadian rhythm	6	0.016
GO:0042475 odontogenesis of dentin-containing tooth	5	0.017
GO:2000144 positive regulation of DNA-templated transcription, initiation	3	0.017
GO:0061072 iris morphogenesis	3	0.017
GO:1901522 positive regulation of transcription from RNA polymerase II promoter involved in cellular response to chemical stimulus	3	0.017
GO:0071356 cellular response to tumor necrosis factor	6	0.017
GO:0048146 positive regulation of fibroblast proliferation	5	0.018
GO:0071300 cellular response to retinoic acid	5	0.018
GO:0008584 male gonad development	6	0.019
GO:0021983 pituitary gland development	4	0.019
GO:0045596 negative regulation of cell differentiation	6	0.02
GO:0043154 negative regulation of cysteine-type endopeptidase activity involved in apoptotic process	5	0.02
GO:0001837 epithelial to mesenchymal transition	4	0.021
GO:2000288 positive regulation of myoblast proliferation	3	0.021
GO:0006473 protein acetylation	3	0.021
GO:0007183 SMAD protein complex assembly	3	0.021
GO:0007249 I-kappaB kinase/NF-kappaB signaling	4	0.022
GO:0043388 positive regulation of DNA binding	4	0.022
GO:0035264 multicellular organism growth	6	0.024
GO:0019827 stem cell population maintenance	5	0.024
GO:0071345 cellular response to cytokine stimulus	4	0.024
GO:0045766 positive regulation of angiogenesis	6	0.024
GO:0050680 negative regulation of epithelial cell proliferation	5	0.024
GO:0010332 response to gamma radiation	4	0.025
GO:0048863 stem cell differentiation	4	0.025
GO:0048709 oligodendrocyte differentiation	4	0.025
GO:0006977 DNA damage response, signal transduction by p53 class mediator resulting in cell cycle arrest	3	0.025
GO:0003208 cardiac ventricle morphogenesis	3	0.025
GO:1902166 negative regulation of intrinsic apoptotic signaling pathway in response to DNA damage by p53 class mediator	3	0.025
GO:0031069 hair follicle morphogenesis	4	0.027
GO:0042177 negative regulation of protein catabolic process	4	0.027
GO:0043627 response to estrogen	5	0.027
GO:0030334 regulation of cell migration	5	0.03
GO:2000773 negative regulation of cellular senescence	3	0.03
GO:0042118 endothelial cell activation	3	0.03
GO:0055007 cardiac muscle cell differentiation	4	0.031
GO:0045747 positive regulation of Notch signaling pathway	4	0.031
GO:0007283 spermatogenesis	10	0.031
GO:0003281 ventricular septum development	4	0.033
GO:0030879 mammary gland development	4	0.033
GO:0051276 chromosome organization	4	0.033
GO:0048661 positive regulation of smooth muscle cell proliferation	5	0.033
GO:0045595 regulation of cell differentiation	4	0.035
GO:2001235 positive regulation of apoptotic signaling pathway	4	0.035
GO:0048701 embryonic cranial skeleton morphogenesis	4	0.035
GO:0033077 T cell differentiation in thymus	4	0.035
GO:0060644 mammary gland epithelial cell differentiation	3	0.035
GO:0032495 response to muramyl dipeptide	3	0.035
GO:0031571 mitotic G1 DNA damage checkpoint	3	0.035

GO:0032496 response to lipopolysaccharide	7	0.036
GO:0042981 regulation of apoptotic process	7	0.037
GO:0007492 endoderm development	4	0.037
GO:0045787 positive regulation of cell cycle	4	0.037
GO:0030335 positive regulation of cell migration	7	0.04
GO:0060740 prostate gland epithelium morphogenesis	3	0.041
GO:0001823 mesonephros development	3	0.041
GO:0003198 epithelial to mesenchymal transition involved in endocardial cushion formation	3	0.047
GO:0070306 lens fiber cell differentiation	3	0.047
GO:0021527 spinal cord association neuron differentiation	3	0.047
GO:0060397 JAK-STAT cascade involved in growth hormone signaling pathway	3	0.047
GO:0060411 cardiac septum morphogenesis	3	0.047
mmu05202:Transcriptional misregulation in cancer	21	0
mmu05200:Pathways in cancer	26	0
mmu05166:HTLV-I infection	22	0
mmu05161:Hepatitis B	16	0
mmu05206:MicroRNAs in cancer	20	0
mmu04350:TGF-beta signaling pathway	11	0
mmu04919:Thyroid hormone signaling pathway	12	0
mmu05215:Prostate cancer	11	0
mmu05220:Chronic myeloid leukemia	10	0
mmu04550:Signaling pathways regulating pluripotency of stem cells	12	0
mmu05210:Colorectal cancer	9	0
mmu05212:Pancreatic cancer	9	0
mmu04110:Cell cycle	11	0
mmu04917:Prolactin signaling pathway	9	0
mmu05168:Herpes simplex infection	13	0
mmu05221:Acute myeloid leukemia	8	0
mmu04380:Osteoclast differentiation	10	0
mmu04068:FoxO signaling pathway	10	0
mmu05216:Thyroid cancer	6	0
mmu05169:Epstein-Barr virus infection	10	0
mmu04310:Wnt signaling pathway	10	0
mmu04390:Hippo signaling pathway	10	0
mmu05321:Inflammatory bowel disease (IBD)	7	0
mmu04010:MAPK signaling pathway	12	0
mmu05162:Measles	9	0
mmu04520:Adherens junction	7	0
mmu05203:Viral carcinogenesis	11	0.001
mmu05222:Small cell lung cancer	7	0.001
mmu05160:Hepatitis C	8	0.002
mmu04668:TNF signaling pathway	7	0.003
mmu04722:Neurotrophin signaling pathway	7	0.006
mmu05164:Influenza A	8	0.007
mmu05213:Endometrial cancer	5	0.007
mmu04064:NF-kappa B signaling pathway	6	0.011
mmu04210:Apoptosis	5	0.011
mmu05140:Leishmaniasis	5	0.012
mmu04620:Toll-like receptor signaling pathway	6	0.012
mmu04024:cAMP signaling pathway	8	0.012
mmu04066:HIF-1 signaling pathway	6	0.012
mmu04630:Jak-STAT signaling pathway	7	0.012
mmu05142:Chagas disease (American trypanosomiasis)	6	0.012
mmu04115:p53 signaling pathway	5	0.013
mmu05205:Proteoglycans in cancer	8	0.014
mmu05211:Renal cell carcinoma	5	0.014
mmu04931:Insulin resistance	6	0.014
mmu04662:B cell receptor signaling pathway	5	0.015
mmu04920:Adipocytokine signaling pathway	5	0.016

mmu05219:Bladder cancer	4	0.021
mmu05152:Tuberculosis	7	0.023
mmu05016:Huntington's disease	7	0.038
mmu05217:Basal cell carcinoma	4	0.041
mmu04915:Estrogen signaling pathway	5	0.041
mmu04922:Glucagon signaling pathway	5	0.043
mmu04660:T cell receptor signaling pathway	5	0.044
mmu04621:NOD-like receptor signaling pathway	4	0.044
mmu05145:Toxoplasmosis	5	0.047
mmu04932:Non-alcoholic fatty liver disease (NAFLD)	6	0.048

Table S27: Enriched GO terms and KEGG pathways with adjusted p-values < 0.05 for the TFs involved in the granulocyte cell fate process.

Enriched terms	count	adj. p-values
GO:0006351 transcription, DNA-templated	130	0
GO:0006355 regulation of transcription, DNA-templated	133	0
GO:0045893 positive regulation of transcription, DNA-templated	83	0
GO:0045944 positive regulation of transcription from RNA polymerase II promoter	94	0
GO:0000122 negative regulation of transcription from RNA polymerase II promoter	81	0
GO:0006357 regulation of transcription from RNA polymerase II promoter	51	0
GO:0045892 negative regulation of transcription, DNA-templated	53	0
GO:0010628 positive regulation of gene expression	29	0
GO:0006366 transcription from RNA polymerase II promoter	19	0
GO:0007275 multicellular organism development	40	0
GO:0010629 negative regulation of gene expression	21	0
GO:0008285 negative regulation of cell proliferation	23	0
GO:0030154 cell differentiation	30	0
GO:0048511 rhythmic process	15	0
GO:0043433 negative regulation of sequence-specific DNA binding transcription factor activity	12	0
GO:0001889 liver development	13	0
GO:0010468 regulation of gene expression	19	0
GO:0007623 circadian rhythm	13	0
GO:0043066 negative regulation of apoptotic process	24	0
GO:1902895 positive regulation of pri-miRNA transcription from RNA polymerase II promoter	8	0
GO:0043401 steroid hormone mediated signaling pathway	10	0
GO:0030182 neuron differentiation	13	0
GO:0008284 positive regulation of cell proliferation	22	0
GO:0042127 regulation of cell proliferation	15	0
GO:0035914 skeletal muscle cell differentiation	9	0
GO:0001701 in utero embryonic development	16	0
GO:0032922 circadian regulation of gene expression	9	0
GO:0042493 response to drug	16	0
GO:0043392 negative regulation of DNA binding	7	0
GO:0045665 negative regulation of neuron differentiation	9	0
GO:0009749 response to glucose	9	0
GO:0060216 definitive hemopoiesis	6	0
GO:0009952 anterior/posterior pattern specification	10	0
GO:0010718 positive regulation of epithelial to mesenchymal transition	7	0
GO:0034097 response to cytokine	9	0
GO:0043065 positive regulation of apoptotic process	15	0
GO:0042593 glucose homeostasis	10	0
GO:0071158 positive regulation of cell cycle arrest	6	0
GO:0045599 negative regulation of fat cell differentiation	7	0
GO:0007179 transforming growth factor beta receptor signaling pathway	8	0
GO:0071356 cellular response to tumor necrosis factor	9	0
GO:0008584 male gonad development	9	0

GO:0003198 epithelial to mesenchymal transition involved in endocardial cushion formation	5	0
GO:0071222 cellular response to lipopolysaccharide	11	0
GO:0030509 BMP signaling pathway	8	0
GO:0071345 cellular response to cytokine stimulus	6	0
GO:0007219 Notch signaling pathway	9	0
GO:0042789 mRNA transcription from RNA polymerase II promoter	5	0
GO:0008283 cell proliferation	11	0
GO:0007568 aging	10	0
GO:0007517 muscle organ development	7	0
GO:0043353 enucleate erythrocyte differentiation	4	0
GO:0043124 negative regulation of I-kappaB kinase/NF-kappaB signaling	6	0
GO:0007492 endoderm development	6	0
GO:0045165 cell fate commitment	7	0.001
GO:0048469 cell maturation	6	0.001
GO:0023019 signal transduction involved in regulation of gene expression	5	0.001
GO:0006915 apoptotic process	16	0.001
GO:0007507 heart development	11	0.001
GO:0045444 fat cell differentiation	7	0.001
GO:0060548 negative regulation of cell death	7	0.001
GO:0007050 cell cycle arrest	7	0.001
GO:0016569 covalent chromatin modification	11	0.001
GO:0030324 lung development	8	0.001
GO:0030308 negative regulation of cell growth	8	0.001
GO:2000188 regulation of cholesterol homeostasis	4	0.001
GO:0030900 forebrain development	7	0.001
GO:0030512 negative regulation of transforming growth factor beta receptor signaling pathway	6	0.002
GO:0007417 central nervous system development	7	0.002
GO:0070301 cellular response to hydrogen peroxide	6	0.002
GO:0042771 intrinsic apoptotic signaling pathway in response to DNA damage by p53 class mediator	5	0.002
GO:0043922 negative regulation by host of viral transcription	4	0.002
GO:0048663 neuron fate commitment	5	0.003
GO:0043523 regulation of neuron apoptotic process	5	0.003
GO:0043923 positive regulation by host of viral transcription	4	0.003
GO:0060411 cardiac septum morphogenesis	4	0.003
GO:0055089 fatty acid homeostasis	4	0.003
GO:0034599 cellular response to oxidative stress	6	0.003
GO:0048863 stem cell differentiation	5	0.003
GO:0060612 adipose tissue development	5	0.003
GO:0090090 negative regulation of canonical Wnt signaling pathway	7	0.003
GO:0071456 cellular response to hypoxia	7	0.003
GO:0045723 positive regulation of fatty acid biosynthetic process	4	0.004
GO:0045669 positive regulation of osteoblast differentiation	6	0.004
GO:0000432 positive regulation of transcription from RNA polymerase II promoter by glucose	3	0.004
GO:0043525 positive regulation of neuron apoptotic process	6	0.004
GO:0048662 negative regulation of smooth muscle cell proliferation	5	0.004
GO:0001568 blood vessel development	6	0.005
GO:0051726 regulation of cell cycle	7	0.005
GO:0042472 inner ear morphogenesis	6	0.005
GO:2000679 positive regulation of transcription regulatory region DNA binding	4	0.005
GO:0001779 natural killer cell differentiation	4	0.005
GO:0001649 osteoblast differentiation	7	0.005
GO:0055088 lipid homeostasis	5	0.006
GO:0030217 T cell differentiation	5	0.006
GO:0071407 cellular response to organic cyclic compound	6	0.006
GO:0071354 cellular response to interleukin-6	4	0.006
GO:0048596 embryonic camera-type eye morphogenesis	4	0.006

GO:0035994 response to muscle stretch	4	0.006
GO:0050679 positive regulation of epithelial cell proliferation	6	0.006
GO:0043010 camera-type eye development	6	0.007
GO:0002076 osteoblast development	4	0.007
GO:0045682 regulation of epidermis development	3	0.007
GO:1900212 negative regulation of mesenchymal cell apoptotic process involved in metanephros development	3	0.007
GO:0048706 embryonic skeletal system development	5	0.007
GO:0071347 cellular response to interleukin-1	6	0.007
GO:0048661 positive regulation of smooth muscle cell proliferation	6	0.007
GO:0060065 uterus development	4	0.008
GO:0017015 regulation of transforming growth factor beta receptor signaling pathway	4	0.008
GO:0043966 histone H3 acetylation	5	0.009
GO:0010942 positive regulation of cell death	5	0.009
GO:0050728 negative regulation of inflammatory response	6	0.01
GO:0032869 cellular response to insulin stimulus	6	0.011
GO:0060956 endocardial cell differentiation	3	0.011
GO:0038061 NIK/NF-kappaB signaling	3	0.011
GO:0031325 positive regulation of cellular metabolic process	3	0.011
GO:0051591 response to cAMP	5	0.011
GO:0001708 cell fate specification	4	0.013
GO:0009987 cellular process	4	0.014
GO:0048646 anatomical structure formation involved in morphogenesis	4	0.014
GO:0071375 cellular response to peptide hormone stimulus	4	0.014
GO:0071499 cellular response to laminar fluid shear stress	3	0.015
GO:0065004 protein-DNA complex assembly	3	0.015
GO:0051574 positive regulation of histone H3-K9 methylation	3	0.015
GO:2001171 positive regulation of ATP biosynthetic process	3	0.015
GO:0031052 chromosome breakage	3	0.015
GO:1901215 negative regulation of neuron death	5	0.015
GO:0045668 negative regulation of osteoblast differentiation	5	0.015
GO:0001756 somitogenesis	5	0.015
GO:0032526 response to retinoic acid	5	0.016
GO:0045662 negative regulation of myoblast differentiation	4	0.017
GO:0030099 myeloid cell differentiation	4	0.019
GO:0009267 cellular response to starvation	5	0.019
GO:0030219 megakaryocyte differentiation	3	0.02
GO:1901522 positive regulation of transcription from RNA polymerase II promoter involved in cellular response to chemical stimulus	3	0.02
GO:0035356 cellular triglyceride homeostasis	3	0.02
GO:0001892 embryonic placenta development	4	0.021
GO:0051091 positive regulation of sequence-specific DNA binding transcription factor activity	6	0.022
GO:0045930 negative regulation of mitotic cell cycle	4	0.023
GO:0071542 dopaminergic neuron differentiation	4	0.023
GO:0043524 negative regulation of neuron apoptotic process	7	0.023
GO:0048146 positive regulation of fibroblast proliferation	5	0.024
GO:0042149 cellular response to glucose starvation	4	0.024
GO:0001958 endochondral ossification	4	0.024
GO:0061314 Notch signaling involved in heart development	3	0.025
GO:0045084 positive regulation of interleukin-12 biosynthetic process	3	0.025
GO:0006473 protein acetylation	3	0.025
GO:2000617 positive regulation of histone H3-K9 acetylation	3	0.025
GO:0001837 epithelial to mesenchymal transition	4	0.026
GO:0050767 regulation of neurogenesis	4	0.026
GO:0030855 epithelial cell differentiation	5	0.027
GO:0043967 histone H4 acetylation	4	0.028
GO:0048147 negative regulation of fibroblast proliferation	4	0.028
GO:0045814 negative regulation of gene expression, epigenetic	3	0.031

GO:0019216 regulation of lipid metabolic process	4	0.031
GO:0071392 cellular response to estradiol stimulus	4	0.031
GO:0010332 response to gamma radiation	4	0.033
GO:0009617 response to bacterium	4	0.035
GO:0016573 histone acetylation	4	0.035
GO:0048589 developmental growth	4	0.035
GO:0001525 angiogenesis	8	0.035
GO:0030851 granulocyte differentiation	3	0.037
GO:0042118 endothelial cell activation	3	0.037
GO:0090009 primitive streak formation	3	0.037
GO:0044321 response to leptin	3	0.037
GO:0048733 sebaceous gland development	3	0.037
GO:0048541 Peyer's patch development	3	0.037
GO:0045786 negative regulation of cell cycle	4	0.037
GO:0007369 gastrulation	4	0.037
GO:0070059 intrinsic apoptotic signaling pathway in response to endoplasmic reticulum stress	4	0.037
GO:0043627 response to estrogen	5	0.038
GO:0007346 regulation of mitotic cell cycle	4	0.04
GO:0060395 SMAD protein signal transduction	5	0.04
GO:0001656 metanephros development	4	0.043
GO:0060412 ventricular septum morphogenesis	4	0.043
GO:0003184 pulmonary valve morphogenesis	3	0.043
GO:0048103 somatic stem cell division	3	0.043
GO:0048708 astrocyte differentiation	3	0.043
GO:1990440 positive regulation of transcription from RNA polymerase II promoter in response to endoplasmic reticulum stress	3	0.043
GO:0009790 embryo development	5	0.043
GO:0006338 chromatin remodeling	5	0.043
GO:0001822 kidney development	6	0.044
GO:0033077 T cell differentiation in thymus	4	0.044
GO:0045787 positive regulation of cell cycle	4	0.047
GO:0001666 response to hypoxia	7	0.047
GO:0008015 blood circulation	3	0.049
GO:0001823 mesonephros development	3	0.049
GO:0006978 DNA damage response, signal transduction by p53 class mediator resulting in transcription of p21 class mediator	3	0.049
GO:0045722 positive regulation of gluconeogenesis	3	0.049
GO:0042640 anagen	3	0.049
mmu05202:Transcriptional misregulation in cancer	23	0
mmu05166:HTLV-I infection	22	0
mmu05161:Hepatitis B	17	0
mmu05200:Pathways in cancer	24	0
mmu04350:TGF-beta signaling pathway	13	0
mmu04550:Signaling pathways regulating pluripotency of stem cells	15	0
mmu05221:Acute myeloid leukemia	9	0
mmu05212:Pancreatic cancer	9	0
mmu05215:Prostate cancer	10	0
mmu05220:Chronic myeloid leukemia	9	0
mmu04917:Prolactin signaling pathway	9	0
mmu05203:Viral carcinogenesis	14	0
mmu05168:Herpes simplex infection	13	0
mmu04919:Thyroid hormone signaling pathway	10	0
mmu04110:Cell cycle	10	0
mmu05206:MicroRNAs in cancer	14	0
mmu05210:Colorectal cancer	7	0.001
mmu04380:Osteoclast differentiation	9	0.001
mmu04068:FoxO signaling pathway	9	0.001
mmu05169:Epstein-Barr virus infection	9	0.001
mmu05133:Pertussis	7	0.001

mmu04310:Wnt signaling pathway	9	0.001
mmu04950:Maturity onset diabetes of the young	5	0.001
mmu04931:Insulin resistance	8	0.001
mmu05216:Thyroid cancer	5	0.002
mmu05222:Small cell lung cancer	7	0.002
mmu04932:Non-alcoholic fatty liver disease (NAFLD)	9	0.002
mmu05321:Inflammatory bowel disease (IBD)	6	0.002
mmu05162:Measles	8	0.004
mmu05160:Hepatitis C	8	0.004
mmu05152:Tuberculosis	9	0.004
mmu04520:Adherens junction	6	0.006
mmu04390:Hippo signaling pathway	8	0.007
mmu04722:Neurotrophin signaling pathway	7	0.01
mmu05223:Non-small cell lung cancer	5	0.014
mmu04922:Glucagon signaling pathway	6	0.02
mmu05140:Leishmaniasis	5	0.02
mmu04620:Toll-like receptor signaling pathway	6	0.021
mmu04630:Jak-STAT signaling pathway	7	0.021
mmu04662:B cell receptor signaling pathway	5	0.026
mmu04668:TNF signaling pathway	6	0.026
mmu04920:Adipocytokine signaling pathway	5	0.028
mmu05219:Bladder cancer	4	0.033
mmu05164:Influenza A	7	0.039
mmu04152:AMPK signaling pathway	6	0.044
mmu05030:Cocaine addiction	4	0.049
mmu04330:Notch signaling pathway	4	0.049

Table S28: Enriched GO terms and KEGG pathways with adjusted p-values < 0.05 for the TFs involved in the monocyte cell fate process.

Enriched terms	count	adj. p-values
GO:0006351 transcription, DNA-templated	53	0
GO:0045944 positive regulation of transcription from RNA polymerase II promoter	46	0
GO:0006355 regulation of transcription, DNA-templated	55	0
GO:0045893 positive regulation of transcription, DNA-templated	35	0
GO:0000122 negative regulation of transcription from RNA polymerase II promoter	34	0
GO:0010628 positive regulation of gene expression	22	0
GO:0006357 regulation of transcription from RNA polymerase II promoter	22	0
GO:0045892 negative regulation of transcription, DNA-templated	20	0
GO:0034097 response to cytokine	10	0
GO:0042493 response to drug	12	0
GO:1902895 positive regulation of pri-miRNA transcription from RNA polymerase II promoter	6	0
GO:0043525 positive regulation of neuron apoptotic process	7	0
GO:0043066 negative regulation of apoptotic process	13	0
GO:0032496 response to lipopolysaccharide	9	0
GO:0006366 transcription from RNA polymerase II promoter	8	0
GO:0051591 response to cAMP	6	0
GO:0003151 outflow tract morphogenesis	6	0
GO:0035914 skeletal muscle cell differentiation	6	0
GO:2000144 positive regulation of DNA-templated transcription, initiation	4	0
GO:0007507 heart development	9	0
GO:0009612 response to mechanical stimulus	6	0
GO:0021983 pituitary gland development	5	0
GO:0008285 negative regulation of cell proliferation	10	0
GO:0048511 rhythmic process	7	0
GO:0007179 transforming growth factor beta receptor signaling pathway	6	0
GO:0071407 cellular response to organic cyclic compound	6	0
GO:0010468 regulation of gene expression	9	0

GO:0001889 liver development	6	0
GO:0007568 aging	7	0.001
GO:0001706 endoderm formation	4	0.001
GO:0035994 response to muscle stretch	4	0.001
GO:0045670 regulation of osteoclast differentiation	4	0.001
GO:0048596 embryonic camera-type eye morphogenesis	4	0.001
GO:0042789 mRNA transcription from RNA polymerase II promoter	4	0.001
GO:0001701 in utero embryonic development	8	0.001
GO:0008284 positive regulation of cell proliferation	10	0.001
GO:0001764 neuron migration	6	0.001
GO:0071222 cellular response to lipopolysaccharide	7	0.002
GO:0030182 neuron differentiation	6	0.002
GO:0023019 signal transduction involved in regulation of gene expression	4	0.002
GO:0030902 hindbrain development	4	0.002
GO:0045669 positive regulation of osteoblast differentiation	5	0.002
GO:0001892 embryonic placenta development	4	0.003
GO:0060395 SMAD protein signal transduction	5	0.003
GO:0038061 NIK/NF-kappaB signaling	3	0.003
GO:0030154 cell differentiation	11	0.003
GO:0048663 neuron fate commitment	4	0.004
GO:0060021 palate development	5	0.004
GO:0010629 negative regulation of gene expression	7	0.004
GO:0007275 multicellular organism development	12	0.006
GO:0007492 endoderm development	4	0.007
GO:0036302 atrioventricular canal development	3	0.007
GO:0007623 circadian rhythm	5	0.008
GO:0009952 anterior/posterior pattern specification	5	0.009
GO:0051726 regulation of cell cycle	5	0.009
GO:0032870 cellular response to hormone stimulus	4	0.01
GO:0045766 positive regulation of angiogenesis	5	0.012
GO:0048643 positive regulation of skeletal muscle tissue development	3	0.012
GO:1990440 positive regulation of transcription from RNA polymerase II promoter in response to endoplasmic reticulum stress	3	0.012
GO:0048617 embryonic foregut morphogenesis	3	0.012
GO:0007219 Notch signaling pathway	5	0.013
GO:0030324 lung development	5	0.013
GO:0071277 cellular response to calcium ion	4	0.013
GO:0042127 regulation of cell proliferation	6	0.014
GO:0001822 kidney development	5	0.014
GO:0003198 epithelial to mesenchymal transition involved in endocardial cushion formation	3	0.015
GO:0032922 circadian regulation of gene expression	4	0.018
GO:0001947 heart looping	4	0.018
GO:0061029 eyelid development in camera-type eye	3	0.019
GO:0030224 monocyte differentiation	3	0.024
GO:0001709 cell fate determination	3	0.027
GO:0060216 definitive hemopoiesis	3	0.027
GO:0045165 cell fate commitment	4	0.027
GO:0043524 negative regulation of neuron apoptotic process	5	0.027
GO:0072520 seminiferous tubule development	3	0.028
GO:0048661 positive regulation of smooth muscle cell proliferation	4	0.034
GO:0043153 entrainment of circadian clock by photoperiod	3	0.034
GO:0060045 positive regulation of cardiac muscle cell proliferation	3	0.039
GO:0035050 embryonic heart tube development	3	0.039
GO:0050728 negative regulation of inflammatory response	4	0.042
GO:0030509 BMP signaling pathway	4	0.042
GO:0060441 epithelial tube branching involved in lung morphogenesis	3	0.042
GO:0009987 cellular process	3	0.042
GO:0060070 canonical Wnt signaling pathway	4	0.043
GO:0009416 response to light stimulus	3	0.044

mmu05161:Hepatitis B	14	0
mmu05166:HTLV-I infection	14	0
mmu05202:Transcriptional misregulation in cancer	10	0
mmu04380:Osteoclast differentiation	9	0
mmu05168:Herpes simplex infection	10	0
mmu05212:Pancreatic cancer	7	0
mmu05203:Viral carcinogenesis	10	0
mmu05200:Pathways in cancer	12	0
mmu05321:Inflammatory bowel disease (IBD)	6	0
mmu04662:B cell receptor signaling pathway	6	0
mmu04010:MAPK signaling pathway	9	0
mmu05169:Epstein-Barr virus infection	7	0.001
mmu05215:Prostate cancer	6	0.001
mmu04310:Wnt signaling pathway	7	0.001
mmu04620:Toll-like receptor signaling pathway	6	0.001
mmu04660:T cell receptor signaling pathway	6	0.001
mmu04022:cGMP-PKG signaling pathway	7	0.001
mmu04668:TNF signaling pathway	6	0.002
mmu05140:Leishmaniasis	5	0.002
mmu05210:Colorectal cancer	5	0.002
mmu05220:Chronic myeloid leukemia	5	0.003
mmu04917:Prolactin signaling pathway	5	0.003
mmu05133:Pertussis	5	0.003
mmu05160:Hepatitis C	6	0.004
mmu05162:Measles	6	0.004
mmu05164:Influenza A	6	0.009
mmu05145:Toxoplasmosis	5	0.01
mmu05030:Cocaine addiction	4	0.01
mmu04722:Neurotrophin signaling pathway	5	0.016
mmu04550:Signaling pathways regulating pluripotency of stem cells	5	0.024
mmu05218:Melanoma	4	0.024
mmu05132:Salmonella infection	4	0.029
mmu04921:Oxytocin signaling pathway	5	0.03
mmu04950:Maturity onset diabetes of the young	3	0.031
mmu05222:Small cell lung cancer	4	0.033
mmu04350:TGF-beta signaling pathway	4	0.034
mmu05152:Tuberculosis	5	0.044
mmu04915:Estrogen signaling pathway	4	0.046

Table S29. Network statistics of the six blood lineages.

Cell	# nodes	# edges
CD4	245	259
CD8	272	293
B-cell	268	290
Eryth	157	179
Granu	199	209
Mono	81	66

Table S30. Genes that belong to the shortest path connecting the influencers in the CD4 T-cell.

Gene	role	out_degree	in_degree
Jun	influencer	4	17
Tnfsf13b	influencer	0	6
Ctnnb1	connector	4	0
Fli1	influencer	3	7
Gja1	influencer	0	6
Smad3	connector	5	0
Mycn	influencer	0	14
Tal1	influencer	2	6
Sp1	connector	15	0
Erg	influencer	2	9
Nfatc1	influencer	1	7
Cdkn2b	influencer	0	12
Met	influencer	0	13
Smad6	influencer	0	9
Cdkn1c	influencer	0	6

Table S31. Genes that belong to the shortest path connecting the influencers in the CD8 T-cell.

Gene	role	out_degree	in_degree
Jun	influencer	3	17
Tnfsf13b	influencer	0	6
Mitf	influencer	0	10
Ctnnb1	connector	4	0
Fli1	influencer	4	7
Gja1	influencer	0	6
Smad3	connector	5	0
Mycn	influencer	0	14
Tal1	influencer	3	6
Sp1	connector	14	0
Bax	influencer	0	13
Sox9	influencer	1	19
Gata3	connector	2	0
Erg	influencer	1	9
Cdkn2b	influencer	0	12
Nfatc1	influencer	0	7
Smad6	influencer	0	9
Cdkn1c	influencer	0	6
Runx2	connector	4	0

Table S32. Genes that belong to the shortest path connecting the influencers in the B-cell.

Gene	role	out_degree	in_degree
Jun	influencer	4	17
Tnfsf13b	influencer	0	6
Trp53	connector	7	0
Mitf	influencer	1	10
Mt1	influencer	0	16
Ctnnb1	connector	5	0
Rela	connector	4	0
Fli1	influencer	4	7
Gja1	influencer	0	6
Mycn	influencer	0	14
Tal1	influencer	3	6
Sp1	connector	16	0
Gata3	influencer	2	6
Erg	influencer	2	9
Nfatc1	influencer	1	7
Smad6	influencer	0	9
Met	influencer	0	13
Cdkn1c	influencer	0	6

Table S33. Genes that belong to the shortest path connecting the influencers in the erythrocytes.

Gene	role	out_degree	in_degree
Egr1	connector	5	0
Tgfb2	influencer	0	6
Trp53	influencer	4	55
Sp1	connector	9	0
Vegfa	influencer	0	24
Bax	influencer	0	13
E2f1	connector	3	0
Id3	influencer	0	10
Lpl	influencer	0	7
Sod2	influencer	0	8

Table S34. Genes that belong to the shortest path connecting the influencers in the granulocytes.

Gene	role	out_degree	in_degree
Egr2	influencer	0	8
Nr0b2	influencer	1	10
Ppard	connector	2	0
Snai2	influencer	0	10
Il12a	influencer	0	8
Gtf2i	connector	2	0
Tgfb1	influencer	0	14
Tgfb2	influencer	0	6
Sp1	connector	12	5
Kit	influencer	0	7
Fasn	influencer	0	7
Id1	influencer	1	6
Myb	connector	2	0
Oprm1	influencer	0	6
Alpl	influencer	0	8
Nfkb1	connector	8	0
Tcf3	connector	3	0
Srebf1	connector	5	0

Table S35. Genes that belong to the shortest path connecting the influencers in the monocytes.

Gene	role	out_degree	in_degree
Tgfb2	influencer	0	6
Sp1	connector	2	0
Pax3	connector	2	0
Nfatc1	influencer	1	7
Met	influencer	0	13

Table S36. Enriched GO terms and KEGG pathways with adjusted p-values < 0.05 and Mus musculus background for the genes and TFs involved in the regulatory pathway of the CD8 T-cell lineage.

Enriched terms	count	adj. p-values
GO:0045893 positive regulation of transcription, DNA-templated	12	0
GO:0045944 positive regulation of transcription from RNA polymerase II promoter	12	0
GO:0008285 negative regulation of cell proliferation	9	0
GO:0006357 regulation of transcription from RNA polymerase II promoter	9	0
GO:0010628 positive regulation of gene expression	9	0
GO:0006355 regulation of transcription, DNA-templated	14	0
GO:0006351 transcription, DNA-templated	13	0
GO:0000122 negative regulation of transcription from RNA polymerase II promoter	9	0
GO:0001501 skeletal system development	5	0
GO:2000144 positive regulation of DNA-templated transcription, initiation	3	0.001
GO:0030219 megakaryocyte differentiation	3	0.001
GO:0010629 negative regulation of gene expression	5	0.007
GO:0001701 in utero embryonic development	5	0.01
GO:0032332 positive regulation of chondrocyte differentiation	3	0.01
GO:1902895 positive regulation of pri-miRNA transcription from RNA polymerase II promoter	3	0.01
GO:0001764 neuron migration	4	0.01
GO:0009987 cellular process	3	0.01
GO:0030182 neuron differentiation	4	0.01
GO:0001822 kidney development	4	0.01
GO:0030316 osteoclast differentiation	3	0.013
GO:0002053 positive regulation of mesenchymal cell proliferation	3	0.021
GO:0030217 T cell differentiation	3	0.024
GO:0048469 cell maturation	3	0.028
GO:0042127 regulation of cell proliferation	4	0.041
GO:0008284 positive regulation of cell proliferation	5	0.047
GO:0042475 odontogenesis of dentin-containing tooth	3	0.047
GO:0042733 embryonic digit morphogenesis	3	0.047
GO:0072182 regulation of nephron tubule epithelial cell differentiation	2	0.047
GO:0043066 negative regulation of apoptotic process	5	0.047
GO:0045669 positive regulation of osteoblast differentiation	3	0.047
GO:0007507 heart development	4	0.047
GO:0043525 positive regulation of neuron apoptotic process	3	0.047
GO:0045892 negative regulation of transcription, DNA-templated	5	0.047
GO:0050680 negative regulation of epithelial cell proliferation	3	0.047
GO:0045165 cell fate commitment	3	0.047
GO:0007179 transforming growth factor beta receptor signaling pathway	3	0.048
GO:0043627 response to estrogen	3	0.048
mmu05166:HTLV-I infection	6	0.007
mmu05210:Colorectal cancer	4	0.007
mmu05202:Transcriptional misregulation in cancer	5	0.007
mmu04350:TGF-beta signaling pathway	4	0.011
mmu05200:Pathways in cancer	6	0.013

Table S37. Enriched GO terms and KEGG pathways with adjusted p-values < 0.05 and Mus musculus background for the genes and TFs involved in the regulatory pathway of the CD4 T-cell lineage.

Enriched terms	count	adj. p-values
GO:0045893 positive regulation of transcription, DNA-templated	8	0
GO:0045944 positive regulation of transcription from RNA polymerase II promoter	9	0
GO:0008285 negative regulation of cell proliferation	6	0.001
GO:0006357 regulation of transcription from RNA polymerase II promoter	6	0.001
GO:0010628 positive regulation of gene expression	6	0.001
GO:0006355 regulation of transcription, DNA-templated	10	0.001
GO:0000122 negative regulation of transcription from RNA polymerase II promoter	7	0.001
GO:0030219 megakaryocyte differentiation	3	0.001
GO:0006351 transcription, DNA-templated	9	0.001
GO:0001889 liver development	4	0.002
GO:1902895 positive regulation of pri-miRNA transcription from RNA polymerase II promoter	3	0.006
GO:0009987 cellular process	3	0.006
GO:0010629 negative regulation of gene expression	4	0.038
GO:0042733 embryonic digit morphogenesis	3	0.041
GO:0001701 in utero embryonic development	4	0.046
GO:0007179 transforming growth factor beta receptor signaling pathway	3	0.046
mmu05202:Transcriptional misregulation in cancer	5	0.009
mmu04350:TGF-beta signaling pathway	4	0.012
mmu05166:HTLV-I infection	5	0.02

Table S38. Enriched GO terms and KEGG pathways with adjusted p-values < 0.05 and Mus musculus background for the genes and TFs involved in the regulatory pathway of the B-cell lineage.

Enriched terms	count	adj. p-values
GO:0045893 positive regulation of transcription, DNA-templated	11	0
GO:0045944 positive regulation of transcription from RNA polymerase II promoter	12	0
GO:0000122 negative regulation of transcription from RNA polymerase II promoter	10	0
GO:0006357 regulation of transcription from RNA polymerase II promoter	8	0
GO:0010628 positive regulation of gene expression	8	0
GO:0006355 regulation of transcription, DNA-templated	13	0
GO:0006351 transcription, DNA-templated	12	0
GO:0010629 negative regulation of gene expression	6	0
GO:2000144 positive regulation of DNA-templated transcription, initiation	3	0.001
GO:0008285 negative regulation of cell proliferation	6	0.001
GO:0034097 response to cytokine	4	0.003
GO:0001889 liver development	4	0.005
GO:0001701 in utero embryonic development	5	0.008
GO:1902895 positive regulation of pri-miRNA transcription from RNA polymerase II promoter	3	0.009
GO:0001764 neuron migration	4	0.009
GO:0009987 cellular process	3	0.009
GO:0042493 response to drug	5	0.009
GO:0030316 osteoclast differentiation	3	0.012
GO:0033077 T cell differentiation in thymus	3	0.021
GO:0042127 regulation of cell proliferation	4	0.039
GO:0043066 negative regulation of apoptotic process	5	0.048
GO:0042733 embryonic digit morphogenesis	3	0.048
GO:0007507 heart development	4	0.048
GO:0045892 negative regulation of transcription, DNA-templated	5	0.048
GO:0072182 regulation of nephron tubule epithelial cell differentiation	2	0.048
GO:0043525 positive regulation of neuron apoptotic process	3	0.048
mmu05202:Transcriptional misregulation in cancer	7	0
mmu05200:Pathways in cancer	6	0.031
mmu05166:HTLV-I infection	5	0.038
mmu04380:Osteoclast differentiation	4	0.038
mmu04310:Wnt signaling pathway	4	0.039
mmu05161:Hepatitis B	4	0.039

Table S39. Enriched GO terms and KEGG pathways with adjusted p-values < 0.05 and Mus musculus background for the genes and TFs involved in the regulatory pathway of the erythrocyte lineage.

Enriched terms	count	adj. p-values
GO:0043525 positive regulation of neuron apoptotic process	4	0.002
GO:0051726 regulation of cell cycle	4	0.005
GO:0010628 positive regulation of gene expression	5	0.005
GO:0001836 release of cytochrome c from mitochondria	3	0.007
GO:0001666 response to hypoxia	4	0.007
GO:0072332 intrinsic apoptotic signaling pathway by p53 class mediator	3	0.007
GO:0042981 regulation of apoptotic process	4	0.007
GO:0048147 negative regulation of fibroblast proliferation	3	0.007
GO:0010332 response to gamma radiation	3	0.007
GO:0001974 blood vessel remodeling	3	0.01
GO:0007507 heart development	4	0.01
GO:0010629 negative regulation of gene expression	4	0.01
GO:0045599 negative regulation of fat cell differentiation	3	0.01
GO:0000122 negative regulation of transcription from RNA polymerase II promoter	5	0.011
GO:0008630 intrinsic apoptotic signaling pathway in response to DNA damage	3	0.011
GO:0009611 response to wounding	3	0.012
GO:0048666 neuron development	3	0.012
GO:0043065 positive regulation of apoptotic process	4	0.014
GO:0042493 response to drug	4	0.014
GO:0008285 negative regulation of cell proliferation	4	0.02
GO:0045944 positive regulation of transcription from RNA polymerase II promoter	5	0.024
GO:0071456 cellular response to hypoxia	3	0.028
GO:0048511 rhythmic process	3	0.041
GO:0001822 kidney development	3	0.041
GO:0043066 negative regulation of apoptotic process	4	0.049
GO:0045893 positive regulation of transcription, DNA-templated	4	0.049
mmu05212:Pancreatic cancer	4	0.003
mmu05166:HTLV-I infection	5	0.006
mmu05161:Hepatitis B	4	0.011
mmu05200:Pathways in cancer	5	0.012
mmu05219:Bladder cancer	3	0.013
mmu05016:Huntington's disease	4	0.014
mmu05210:Colorectal cancer	3	0.022
mmu05220:Chronic myeloid leukemia	3	0.024
mmu05206:MicroRNAs in cancer	4	0.024
mmu04350:TGF-beta signaling pathway	3	0.027

Table S40. Enriched GO terms and KEGG pathways with adjusted p-values < 0.05 and Mus musculus background for the genes and TFs involved in the regulatory pathway of the granulocyte lineage.

Enriched terms	count	adj. p-values
GO:0010628 positive regulation of gene expression	8	0
GO:0000122 negative regulation of transcription from RNA polymerase II promoter	9	0
GO:0045893 positive regulation of transcription, DNA-templated	8	0
GO:0006351 transcription, DNA-templated	11	0
GO:0006355 regulation of transcription, DNA-templated	11	0.001
GO:0009749 response to glucose	4	0.005
GO:0042060 wound healing	4	0.007
GO:0010629 negative regulation of gene expression	5	0.007
GO:0045944 positive regulation of transcription from RNA polymerase II promoter	7	0.014
GO:0032570 response to progesterone	3	0.019
GO:0009314 response to radiation	3	0.019
GO:0001837 epithelial to mesenchymal transition	3	0.019
GO:0097191 extrinsic apoptotic signaling pathway	3	0.031
GO:0016477 cell migration	4	0.031
GO:0032496 response to lipopolysaccharide	4	0.031
GO:0030335 positive regulation of cell migration	4	0.032
GO:0043408 regulation of MAPK cascade	3	0.049
mmu05166:HTLV-I infection	6	0.01
mmu05321:Inflammatory bowel disease (IBD)	4	0.01
mmu05140:Leishmaniasis	4	0.01
mmu04350:TGF-beta signaling pathway	4	0.017
mmu05142:Chagas disease (American trypanosomiasis)	4	0.021
mmu05145:Toxoplasmosis	4	0.021
mmu05146:Amoebiasis	4	0.025
mmu05161:Hepatitis B	4	0.04
mmu04390:Hippo signaling pathway	4	0.04
mmu05144:Malaria	3	0.043
mmu05152:Tuberculosis	4	0.048
mmu05221:Acute myeloid leukemia	3	0.048
